# Characteristics and prognosis of synchronous multiple primary lung cancer after surgical treatment: A systematic review and meta‐analysis of current evidence

**DOI:** 10.1002/cam4.3614

**Published:** 2020-12-10

**Authors:** Hongtao Tie, Jun Luo, Rui Shi, Zhenhan Li, Dan Chen, Qingchen Wu

**Affiliations:** ^1^ Department of Cardiothoracic Surgery The First Affiliated Hospital of Chongqing Medical University Chongqing China; ^2^ Service de Médecine Intensive – Réanimation Hôpital Bicêtre AP‐HP Université Paris‐Saclay Saint‐Aubin France; ^3^ INSERM UMR_S999 LabEx ‐ LERMIT Hôpital Marie‐Lannelongue Le Plessis Robinson France; ^4^ Chongqing Medical University Chongqing China

**Keywords:** prognosis, surgical treatment, synchronous multiple primary lung cancer, systematic review and meta‐analysis

## Abstract

**Background:**

This study aims to quantitatively summary the characteristics of synchronous multiple primary lung cancer (sMPLC), postoperative mortality, long‐term prognosis, and prognostic effects of potential clinical parameters in patients with sMPLC after surgery.

**Methods:**

PubMed and Embase databases were systematically searched to identify studies that explored the prognosis of patients with sMPLC after surgery.

**Results:**

Fifty‐two studies with 3486 participants were included, and clinical characteristics were quantitatively summarized. The pooled proportion of sMPLC in lung cancer was 2.0% (95%CI, 1.6%–2.5%) with an increasing trend over time, and postoperative mortality was 1.4% (95%CI, 0.5%–2.7%) with a decreasing trend over time. The 5‐year survival rate was 44.9% (95%CI, 37.4%–52.6%) and all long‐term survival rates showed increasing trends over time. Poor long‐term prognosis was observed in both limited resection (HR = 1.357, 95%CI, 1.047–1.759, *p* = 0.0210) and pneumonectomy (HR = 2.643, 95%CI, 1.539–4.541, *p* = 0.0004) by comparison of anatomical resection. Other clinical parameters of age, gender, smoking status, FEV1, and lymph node metastasis significantly impacted the long‐term prognosis (all *p* < 0.05).

**Conclusions:**

The proportion of sMPLC in lung cancer and 5‐year survival rate are increasing, while postoperative mortality is decreasing trend over time. Lobectomy should be preferred, while pneumonectomy should be avoided for sMPLC. Age, gender, FEV1, smoking, tumor size, surgical methods, and lymph node status are prognostic factors for sMPLC. Considering the heterogeneity and publication bias, these findings should be treated with caution.

## Graphical abstract

The incidence of sMPLC increases over time, with an estimated incidence of 2.0%. The postoperative mortality decreases, while the long‐term survival rate increases over time.

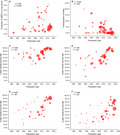

## 1 INTRODUCTION

Synchronous multiple primary lung cancer (sMPLC) is a special type of lung cancer, characterized by at least two primary tumors simultaneously identified in ipsilateral or contralateral lung. SMPLC was first reported by Beyreuther in 1924,^1^ with a great wide range of reported proportion from 0.2% to 6.2% in lung cancer.^2^, ^3^, ^4^, ^5^, ^6^, ^7^, ^8^, ^9^, ^10^, ^11^, ^12^, ^13^, ^14^, ^15^, ^16^, ^17^, ^18^, ^19^, ^20^, ^21^, ^22^, ^23^, ^24^, ^25^, ^26^, ^27^, ^28^, ^29^, ^30^, ^31^, ^32^, ^33^, ^34^, ^35^, ^36^, ^37^, ^38^, ^39^, ^40^, ^41^, ^42^, ^43^, ^44^, ^45^, ^46^, ^47^, ^48^, ^49^, ^50^, ^51^, ^52^, ^53^ With improved imaging techniques, particularly high‐resolution chest computed tomography (CT), and positron emission tomography, increasing number of patients with synchronous multiple lung nodules are being diagnosed,^54^ which of these nodules could be sMPLC or pulmonary metastasis from the lung or other tumor. According to the current conception, surgical resection has been advocated for patients with certain sMPLC, but not pulmonary metastasis for no favorable survival from surgical resection. However, it is not always possible to know the suspicious lung nodules to be sMPLC or pulmonary metastasis preoperatively, and the operative outcome after surgery remains unclear.

With the accumulating concerns on sMPLC, the American Joint Committee on Cancer (AJCC) seventh edition of the cancer tumor‐node‐metastasis (TNM) staging system defined the additional tumor nodules in different location with a disparate stage. In detail, nodules in the same lobe were staged as T3M0, multiple ipsilateral lobes as T4M0, and contralateral lobes as M1A.^55^ However, this classification schema could not reliably predict the prognosis of patients with sMPLC because these it based upon the supposition that multiple lung nodules are intrapulmonary metastases originated from the primary lung cancer, rather than sMPLC.^56^ The prognostic appropriateness of such a categorization necessitates further evaluation. Additionally, special features of tumor size, number of tumors, and histology between tumors in sMPLC were not considered in this staging system.

As reported in the current literature, the overall prognosis of patients with sMPLC after surgery differed broadly from each other, and the prognostic effect of various clinical parameters was inconsistent.^2^, ^3^, ^4^, ^5^, ^6^, ^7^, ^8^, ^9^, ^10^, ^11^, ^12^, ^13^, ^14^, ^15^, ^16^, ^17^, ^18^, ^19^, ^20^, ^21^, ^22^, ^23^, ^24^, ^25^, ^26^, ^27^, ^28^, ^29^, ^30^, ^31^, ^32^, ^33^, ^34^, ^35^, ^36^, ^37^, ^38^, ^39^, ^40^, ^41^, ^42^, ^43^, ^44^, ^45^, ^46^, ^47^, ^48^, ^49^, ^50^, ^51^, ^52^, ^53^ The shortage of proper staging system and lack of statistical overall survival (OS) of sMPLC might ascribe to its rare occurrence. With the increasing concern and accumulating evidence, but little knowledge and considerable controversy about sMPLC, we performed current systematic review and meta‐analysis of current evidence to investigate the characteristics of sMPLC and postoperative mortality. Additionally, the prognosis after surgical treatment and prognostic effects of various potential clinical parameters in patients with sMPLC was explored.

## 2 MATERIALS AND METHODS

The protocol of the current study was registered in an international database of PROSPERO (http://www.crd.york.ac.uk/PROSPERO/), with a registered ID: CRD42018099789. We performed and reported the study following the Preferred Reporting Items for Systematic Reviews and Meta‐Analyses (PRISMA) guidelines and Meta‐analyses and Meta‐analysis of Observational Studies in Epidemiology (MOOSE) guidelines.^57^, ^58^ Since all analyses are based on previous published studies, no ethical approval and patient consent are required.

### 2.1 Literature search

We conducted a systematical literature search in PubMed and Embase (from the inception to Oct 2019). The search strategy was performed by two investigators independently, and the detailed search strategy is in Data S1. In addition to database retrieval, references list of the retrieved studies, and review articles were also screened, and the process was performed repeatedly until no further eligible studies included. Conference abstracts and non‐English studies eligible for inclusion were also included.

### 2.2 Study selection and criteria

The detailed study selection was achieved through two steps. First, we screened the titles and abstracts as the preliminary selection, and then, read the full texts strictly accord to the criteria. Study selection was performed by two investigators independently, and the discrepancies were indicated and solved by another superior investigator. Eligible studies must meet the following criteria: (a) patients with sMPLC; (b) surgery for the treatment of sMPLC; (c) available survival data for sMPLC. When research with the same population has multiple publications, only the completed and recent one was included. Exclusion criteria: (a) only medicine therapy for patients with sMPLC; (b) unavailable prognostic data; (c) patient with intrapulmonary metastasis.

### 2.3 Data extraction and quality assessment

The following information was extracted in a standard extraction form: first author, year of publication, period of participant enrollment, study area, number of patients with sMPLC, number of patients with lung cancer, gender, age, lesions location, number of lesions, identical histology, surgery type, postoperative mortality, and survival rate. Patients’ characteristics (gender, age, FEV1, smoking status, lesions location, number of lesions, histology, surgery type, lymph metastasis, and adjunctive therapy) with corresponding 5‐year survival rate and hazard ratios (HRs) for OS and 95%CIs were extracted as a separate standard form. Survival rate and HRs and 95%CIs from studies concealing results in Kaplan–Meier curves were estimated by software of GetData.Graph.Digitizer.v2.24 (http://getdata‐graph‐digitizer.com/). If some essential data were unavailable, the corresponding authors would be contacted.

The risk of bias in each study was assessed using the modified Newcastle–Ottawa quality scale^59^, ^60^ (Data S1) to fit our included studies, with a maximum of 7 points. The higher scores mean lower risk of bias, and studies were evaluated as high quality (6–7 stars).

### 2.4 Data analysis

In order to present trends over time, bubble charts were applied to describe the distribution between publication year and the corresponding proportion of sMPLC in lung cancer, postoperative mortality rate, and survival rates of sMPLC. Every bubble represents one study, and publication year is for X‐axis, Y‐axis for the corresponding rate, and the area of the bubble for corresponding sample size. Bubble charts were performed with OriginPro 9.1 (OriginLab Corporation). Additionally, correlation relationships between publication years and the rates were analyzed by Spearman's rank correlation in R software.

Events and totals were used to perform the meta‐analysis for the proportion of sMPLC in lung cancer, postoperative mortality, proportions of female, three or more tumors, unilateral lung, and same histology in sMPLC, and means with standard deviations were used for age. Both survival rates and HRs with 95%CIs were used for prognosis analysis. For pooling survival rates, we presumed that no loss to follow up occurred in all included studies, and the events of survival could be calculated by total patients multiplying the corresponding survival rate. All meta‐analyses were performed by using random‐effects models. For proportion, postoperative mortality, and survival rates, meta‐analysis was conducted by Freeman–Tukey double arcsine transformation arcsine^61^ in R software with package meta and metaprop. Log‐transform and R software with package meta and metagen were used for meta‐analysis of HRs with 95%CIs.

Heterogeneity among the studies was assessed by *I* ^2^ statistic, which represents the percentage of variation attributed to heterogeneity instead of chance error.^62^ Subgroup analysis was performed for the proportion of sMPLC, postoperative mortality, and 5‐year OS rate. Sensitivity analysis by omitting one study in each turn was also performed. Predesigned additional analyses for 5‐year survival rate and effect sizes of HRs with 95%CIs were performed to explore the prognostic role of the clinical parameters: Age (older vs. young), gender (female vs. male), smoke status (smoker vs. nonsmoker), FEV1 (high vs. low), numbers of tumors (more than two tumors vs. two tumors), location (unilateral vs. bilateral), surgical methods (anatomical vs. limited vs. pneumonectomy), tumor histology (same vs. different), lymph node metastasis (yes vs. no), and postoperative adjunctive therapy (yes vs. no).

Publication bias was assessed with Funnel plots and Egger's test.^63^ All statistical analyses were performed by using R, version 3.4.0, and two‐tailed *p*‐values less than 0.05 was as statistically significant.

## 3 RESULTS

### 3.1 Characteristics of eligible studies

**FIGURE 1:**
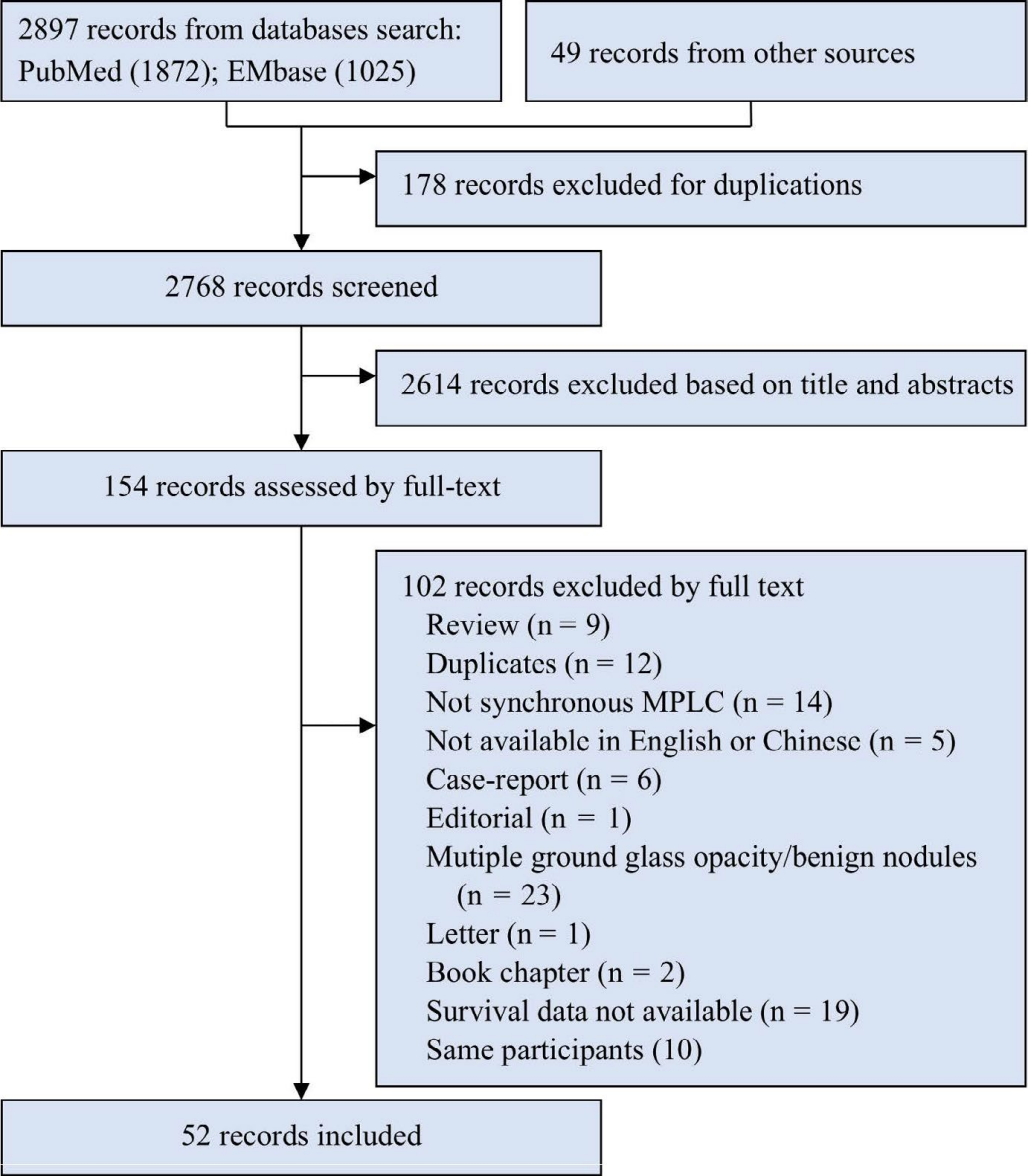
Flow chart of study selection

The flow chart of the detailed selection process is presented in Figure 1 and the basic characteristics of included studies are summarized in Table 1. The literature search yielded 2897 records and 52 studies were included. Tumor location, outcomes of postoperative mortality, survival rates, and HRs with 95%CIs and 5‐year survival rate regarding various clinical parameters displayed in Data S1. All included studies were longitudinal studies. They were published between 1987 and 2018, and sample sizes of sMPLC ranged from 8 to 438, with a total of 3486. For characteristic analysis, 38 studies with 2678 patients reported the gender ratio, 37 studies with 2604 patients reported the ages, 28 studies with 2169 patients reported the proportion of more than two tumors, 44 studies with 2819 patients reported the tumor location, and 44 studies with 3159 patients reported the proportion of identical histology. Most studies were adjudicated to be of high quality with a score of more than 5 (Data S1).

**TABLE 1:** Characteristic of included studies

Study ID	Recruitment time	Area	SMPLC/LC	Female/Male	Age (years)	Three or more tumors	Identical histology
Adebonojo 1997	1984–1995	USA	15/1325	NA	NA	NA	3/15
Andriolo 2012	2000–2010	Italy	13/NA	NA	NA	NA	NA
Angeletti 1995	1980–1993	Italy	18/1960	0/18	62.3 ± 6.84	NA	6/18
Antakli 1995	1966–1994	USA	26/1572	NA	NA	6/26	NA
Aziz 2002	1985–1999	UK	10/892	2/8	54 ± 6.1	NA	7/10
Bae 2012	1990–2008	Korea	19/1852	6/13	60.8 ± 7.69	NA	8/19
Chang 2007	1990–2006	Taiwan	92/1651	40/52	64 (37–83)	54/92	89/92
Chen 2015	2007–2014	China	108/1868	69/34	60 ± 10	14/103	97/103
Cheng 2017	2007–2012	China	45/2138	NA	NA	NA	0/45
De Leyn 2008	1990–2007	Belgium	57/3399	7/29	64.5 (37–80)	NA	18/36
Deschamps 1990	NA	USA	36/NA	NA	NA	NA	NA
Fabian 2011	1996–2009	USA	67/NA	37/30	70.9 ± 8.7	NA	29/67
Feng 2005	1983–2004	China	31/4649	12/19	63 (32–83)	NA	9/31
Finley 2010	1995–2006	USA	175/NA	108/67	69 (42–87)	50/175	146/175
Guo 2017	2010–2015	China	357/25339	248/109	60 (28–80)	88/357	331/357
Hardavella 2018	2013–2017	Greece	8/NA	3//5	NA	NA	NA
Hsu 2016	2006–2012	China	35/564	24/11	65 (49–82)	9/35	NA
Ishikawa2014	1995–2009	Japan	93/2041	57/36	68 (49–84)	22/93	NA
Jung 2011	1995–2008	South Korea	32/4286	10/22	65 (30–77)	3/32	14/32
Kocaturk 2011	2001–2008	Turkey	26/1355	0/26	59.6 (48–77)	2/26	17/26
Li 2011	1999–2009	China	72/NA	18/54	66 (37–79)	NA	33/72
Li 2017	2009–2011	China	36/1948	14/22	59.7 ± 9.6	NA	33/36
Lin 2014	2005–2012	Taiwan	64/1223	46/18	60 (27–81)	11/64	64/64
Liu 2016	1990–2010	China	122/13587	68/54	61.5 ± 7.6	21/122	88/122
Liu 2017	NA	China	438/NA	NA	NA	NA	339/438
lv 2018	2010–2016	China	48/3031	NA	NA	NA	NA
Mun2007	1999–2004	Japan	19/674	13/6	65.8 (52–80)	12/19	10/19
Okada 1998	1985–1996	Japan	28/908	4/24	64.3 ± 7.7	NA	12/28
Peng 2017	2012–2016	China	43/NA	26/17	58 (39–76)	9/43	38/43
Pommier 1996	1974–1994	USA	27/3034	1/26	63 (17–78)	NA	15/27
Rea 2001	1971–1999	Italy	19/NA	NA	NA	NA	13/19
Ribet 1995	1971–1990.	France	24/1980	0/24	57 (40–70)	0/24	14/24
Riquet 2008	1983–2005	France	118/3427	26/92	63.2 ± 10	14/118	60/118
Rosengart 1991	1955–1990	USA	33/NA	NA	63	0/33	16/33
Rostad 2008	1993–2000	Norway	94/2528	40/54	64 (41–83)	28/94	85/94
Shah 2012	1997–2010	USA	47/NA	24/23	66 ± 9	NA	30/47
Takamochi 2012	1996–2008	Japan	31/1047	17/14	65.8 ± 9.0	7/31	13/31
Tanvetyanon 2010	1997–2008	USA	116/2582	69/47	70 (45–86)	NA	80/116
Trousse 2007	1985–2006	France	125/2785	27/98	61.5 ± 9.9	2/125	104 /125
Tsunezuka 2004	1973–2001	Japan	19/1906	NA	70 (51–82)	NA	10/19
Tung 2003	1983–2001	Taiwan	20/1406	9//11	66.4 ± 10.6	3/20	20//20
van Rens 2000	1970–1997	Netherlands	85/3372	9/76	65.8	NA	50/73
Vansteenkiste 2001	1990–1994	Belgium	35/NA	NA	NA	0/35	30/35
Verhagen 1994	1970–1990	Netherlands	15/1287	NA	NA	1/15	11/15
Voltolini 2010	1990–2007	Italy	50/1551	3/40	66.7 ± 6.9	6/43	27/43
Wang 2001	1977–1999	China	12/2384	1//11	55 (32–83)	NA	2//12
Wang 2008	1995–2005	China	16/2832	7//9	64.5 (30–74)	NA	1//16
Wu 1987	1957–1984	China	10/3815	NA	56	1/10	5/10
Xiao 2017	2004–2015	China	52/3527	16/36	60.3 ± 8.61	2/52	20/52
Yu 2013	2001–2011	Taiwan	97/1995	55/42	64.38 ± 12.11	10/97	38/97
Zhang 2016	2010–2014	China	285/NA	159/126	60 (39–78)	51/285	260/285
Zuin 2013	1995–2010	Italy	23/4480	NA	NA	NA	NA

Abbreviations: LC, lung cancer; NA = Not available; SMPLC, synchronous multiple primary lung cancer.

### 3.2 Characteristics of the patients with sMPLC

The detailed pooled effect size of characteristics in patients with sMPLC is summarized in Table 2. Meta‐analysis with random‐effects model showed that, in sMPLC, the female gender ratio was 36.7%; the average age was 63.35 years; the proportion of more than two tumors was 15.4%; the proportion of tumors located in the unilateral lung was 62.4%; the proportion of tumors with identical histology was 63.0%.

**TABLE 2:** Summary Characteristics of the included patients with sMPLC

Clinical data	Studies involved	N involved	Pooled results	I2
ALL‐included sMPLC patients	52	3486	"/>	"/>
**Percentage of sMPLC/LC**	39	2215/122200	0.020 [0.016 to 0.025]	97%
Synchronous MPLC	39	2215		
LC		122200		
Synchronous MPLC from unknown LC	13	1271/NA		
Gender analysis (Percentage of female)	38	1275/2678	0.367 [0.293 to 0.444]	93%
Female	38	1275		
Male		1403		
Not reported	14	808		
Age analysis	37	2604	63.35 [60.65 to 66.05]	0%
Percentage of three or more tumors	27	426/2169	0.154 [0.107 to 0.208]	89%
Three or more tumors	27	426		
Two tumors		1743		
Not reported	25	1284		
Location (Percentage of unilateral)	44	1860/2819	0.624 [0.548 to 0.697]	93%
Same lobe	26	660		
Unilateral different lobes	26	872		
Unilateral	14	328		
Bilateral	43	959		
Not reported	8	634		
Histology (Percentage of same)	44	2295/3159	0.630 [0.547; 0.710]	95%
Same	44	2295		
Different		864		
Not reported	8	294		
Prognosis analysis	52	3432		
Postoperative mortality	42	68/2505	0.014 [0.005 to 0.027]	64%
1‐year survival rate	37	1682	0.868 [0.819 to 0.912]	85%
2‐year survival rate	34	1624	0.717 [0.636 to 0.791]	91%
3‐year survival rate	39	2085	0.629 [0.543 to 0.712]	93%
5‐year survival rate	48	3217	0.449 [0.374 to 0.526]	94%

Abbreviations: LC = lung cancer; sMPLC = synchronous multiple primary lung cancer.

### 3.3 Proportion of sMPLC in lung cancer

**FIGURE 2:**
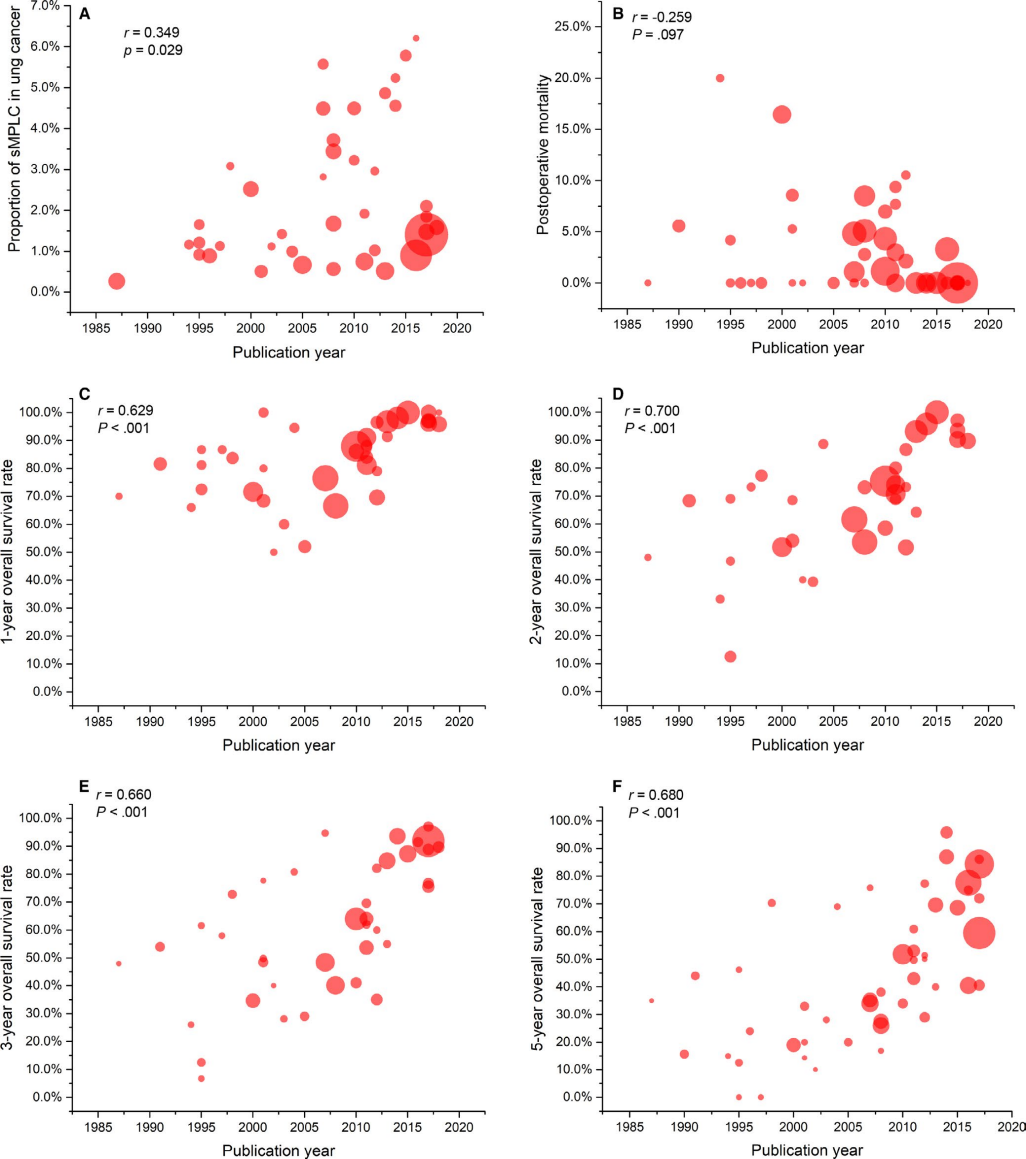
Distributions between publication year and (A) proportion of sMPLC in lung cancer, (B) postoperative mortality rate, (C) 1‐year survival rate, (D) 2‐year survival rate, (E) 3‐year survival rate, (F) 5‐year survival rate. Every bubble represents one study, and publication year is for X‐axis, Y‐axis for the corresponding rate, and the area of the bubble for corresponding sample size. Correlation relationships were analyzed by Spearman's rank correlation. *r* means correlation coefficient and *p* means statistical test

**FIGURE 3:**
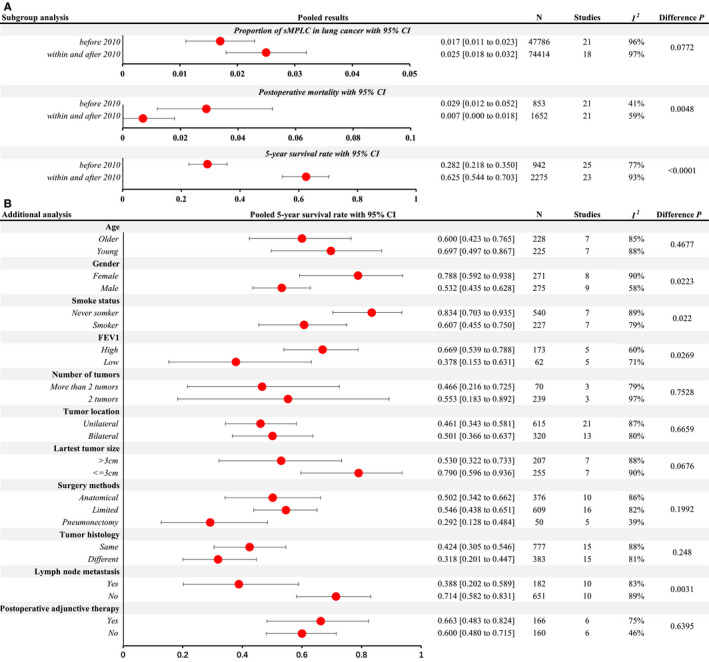
Subgroup analysis and additional analysis. (A) Subgroup analysis according to publication year of proportion of sMPLC in lung cancer, postoperative mortality rate, and 5‐year survival rate. (B) Additional analysis of 5‐year survival rate according to various clinical parameters. All meta‐analyses are performed with random‐effects model, and differences between two subgroups are analyzed by metareg with R package

Thirty‐nine studies, including 2215 sMPLC patients diagnosed from 122,000 patients with primary lung cancer undergoing surgical resection, were used to calculate the proportion of sMPLC, and the pooled result was 2.0% (95%CI, 1.6%–2.5%, Table 2), with significant evidence of between‐study heterogeneity (*I* ^2^ = 97%). Sensitivity analysis by omitting each study in turn revealed that the proportion of sMPLC in LC remained consistently, ranged from 1.9% to 2.1%. The distribution between publication year and the corresponding proportion of sMPLC revealed an increasing trend by visual inspection and a significant positive correlation Spearman analysis (Figure 2A, *r* = 0.349, *p* = 0.029). Subgroup analysis according to publication year showed the proportion of sMPLC was 1.7% (95%CI, 1.1%–2.3%) before 2010 and 2.5% (95%CI, 1.8%–3.2%) after 2010, with an almost significant difference (Figure 3A, *p* = 0.077).

### 3.4 Postoperative mortality

Meta‐analysis of 42 studies, including 68 deaths from 2505 sMPLC patients, showed that the postoperative mortality was 1.4% (95%CI, 0.5%–2.7%, *I* ^2^ = 64%, Table 2 and Data S1). Sensitivity analysis by omitting each study in turn showed that postoperative mortality ranged from 1.1% to 1.6%. A decreased trend of postoperative mortality over time was observed in the bubble charts, with a nonsignificant negative correlation (Figure 2B, *r* = −0.259, *p* = 0.097). While a significant difference between postoperative mortality before and after 2010 [2.9% (95%CI, 1.2%–5.2%) vs. 0.7% (95%CI, 0.0%–1.8%)] was detected in subgroup analysis (Figure 3A, *p* = 0.0048).

### 3.5 Survival rate of sMPLC

The overall results demonstrated the 1‐, 2‐, 3‐, and 5‐year survival rates were 86.8% (95%CI, 81.9%–91.2%, *I* ^2^ = 85%) by pooling 37 studies with 1682 participants, 71.7% (95%CI, 63.6%–79.1%, *I* ^2^ = 91%) by pooling 34 studies with 1624 participants, 62.9% (95%CI, 54.3%–71.2%, *I* ^2^ = 93%) by pooling 39 studies with 2085 participants, and 44.9% (95%CI, 37.4%–52.6%, *I* ^2^ = 94%) by pooling 48 studies with 3217 participants (Table 2 and Data S1). Sensitivity analysis by omitting each study in turn revealed that the 5‐year survival rates ranged from 43.7% to 46.2%, respectively. Increased trends of 1‐, 2‐, 3‐, and 5‐year survival rates over time and significant positive correlations were observed (Figure 2C–F, *r* = 0.629, 0.700, 0.660, 0.680, respectively, and all *p* < 0.001). Additionally, there is a significant difference between 5‐year survival rate before and after 2010 [28.2% (95%CI, 21.8%–35.0%) vs. 62.5% (95%CI, 54.4%–70.3%), Figure 3A, *p* < 0.0001].

### 3.6 Effect of various clinical parameters on 5‐year survival rate

Additional analyses were performed to evaluate the effects of various clinical parameters on the 5‐year survival rate. The forest chart of all subgroup analyses is shown in Data S1. As shown in Figure 3B, female, never smoker, and high FEV1 were associated with a significantly increased 5‐year survival rate (All *p* < 0.05). As mandatory parameters of TNM classification, a better 5‐year survival rate was observed to be significant in patients without lymph node metastasis (*p* = 0.003) and extremely close to significance in patients with the largest tumor size ≤3 cm (*p* = 0.068). While no significant impact on 5‐year survival rate was observed in clinical parameters.

### 3.7 Effect of various clinical parameters on OS

**FIGURE 4:**
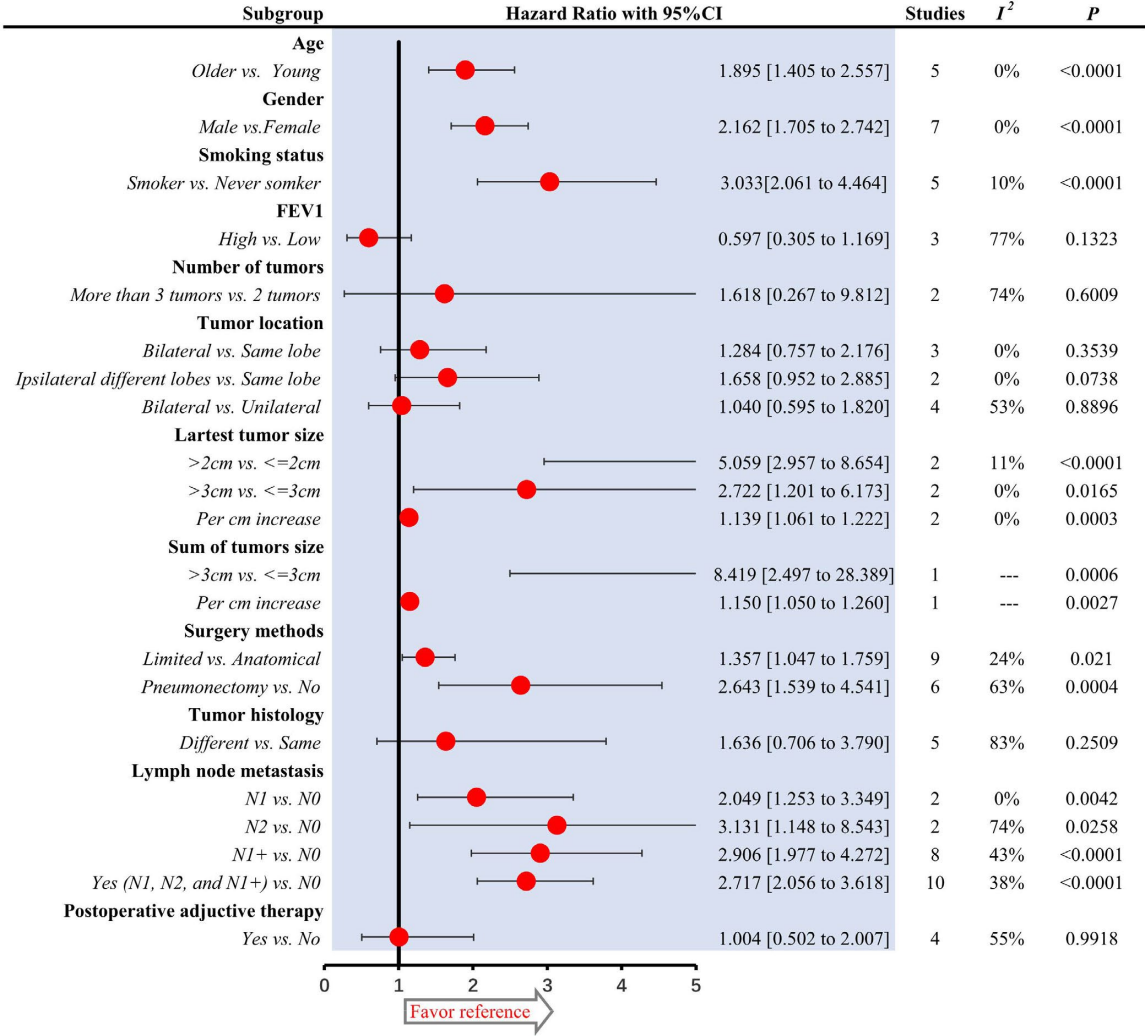
Additional analysis of overall survival according to various clinical parameters. All meta‐analyses were performed with random‐effects model. HR more than 1 means the results favor reference group

Additional analyses by meta‐analyses of HRs with 95%CIs were also executed to assess the effect of various clinical parameters on OS, shown in Figure 4 and Data S1. Consistent with 5‐year survival rate, gender, smoking status, largest tumor size, and lymph node metastasis status have significant impacts on OS, and no significant association between OS and parameters of tumors numbers, tumor location, tumor histology, postoperative adjunctive therapy. Though inconsistency between statistical significance existed in FEV1, age and surgical methods analysis, the trends were consistent. High FEV1 showed a better OS with a slight tendency toward significance [HR = 0.597 (95%CI, 0.305–1.169), *p* = 0.1323]. While for age analysis, the difference of OS between older and young patients reached a statistical significance (HR = 1.895, 95%CI, 1.405–2.557, *p* < 0.0001). For surgical method in sMPLC, limited resection showed a statistically significant worse OS than anatomical resection (HR = 1.357, 95%CI, 1.047–1.759, *p* = 0.021). Pneumonectomy was significantly associated with worse OS by comparison of non‐pneumonectomy (HR = 2.643, 95%CI, 1.539–4.541, *p* = 0.0004).

### 3.8 Publication bias

Asymmetry with smaller studies yielding more extreme estimates were observed in all funnel plots (Data S1) by visual inspection, and evidence of publication bias were also detected by the Egger's test for the proportion of sMPLC (*p* = 0.011), postoperative mortality (*p* = 0.044), and 5‐years survival rate (*p* = 0.001).

## 4 DISCUSSION

This is the first systematic review and meta‐analysis focusing characteristics and prognosis after surgical treatment of sMPLC, with 52 studies involving 3486 sMPLC. The main findings are: (a) The proportion of sMPLC is 2.0% of the lung cancer population with a significantly increasing trend over time, while the postoperative mortality is decreasing over time, with a pooled incidence of 1.4%. (b) The pooled 1‐, 2‐, 3‐, and 5‐year survival rates were 86.8%, 71.7%, 62.9%, and 44.9%, with significant increasing trends over time. (c) Age, gender, FEV1, smoking status, tumor size, surgical methods, and lymph node status are important prognostic factors for patients with sMPLC after surgery. In this study, we gave an overview of characteristics, prognosis, and prognostic factors of sMPLC after surgery, with expected substantial heterogeneity and evidence of publication bias among included studies.

With the establishment of the lung cancer screening program and improvement of screening techniques, it has been reported that patients diagnosed with sMPLC are increasing globally.^64^ Convincingly, a significant increasing trend of proportion of sMPLC over time was found in our study, and subgroup analysis according to publication time also consolidates this finding. Additionally, we provided a precise proportion of sMPLC in lung cancer. We also found that the average age of 63.4 years and female percentage of 36.7% were similar to patients with single lung cancer.^65^, ^66^ Though it is quite difficult to differentiate sMPLC from intrapulmonary metastases and inflammatory nodules by tumor number, histology, and tumor location,^56^ it is still worth to explore their effect on the prognosis of sMPLC.

Database study involving multiple centers reported that postoperative mortality of lung cancer was 2.2%.^67^ With the early stage cases increased, appropriate preoperative evaluation and perioperative management,^68^ the mortality was decreased over time. In sMPLC, we also found a decreased trend of postoperative mortality over time, with a pooled estimated incidence of 1.4%, which was lower than the single primary lung cancer. This could be explained by the small sample size, more prudential surgery selection, and lung‐preserved procedure preferred in our study. According to the eighth edition of the TNM classification for lung cancer,^69^ the estimated survival rates in patients with sMPLC were similar to IIB‐IIIA stage, with 2‐ and 5‐year survival rate of 71.6% and 44.9%. The widely accepted sMPLC staging method is according to the location of separate tumor nodules relative to the main tumor, classifying as T3 in the same lobe, T4 in ipsilateral different lobe, and M1a in contralateral lobe.^69^ A study confirmed the applicability of this classification method for intrapulmonary metastases, with similar OS to other T3, T4, and M1a tumors in single lung cancer.^70^, ^71^ However, whether this classification could predict the prognosis of sMPLC remains unclear, and differentiating sMPLC from intrapulmonary metastases is still a big challenge. Another proposed classification system by IASLC stated that all sMPLC nodules should be staged separately and the highest T category with the number of tumors in parenthesis (e.g., T2(m)NxMx) should be used to indicate the T stage of disease.^71^, ^72^ This staging method seems to consider the disease status comprehensively with all tumors involved, but it cannot reach a specific TNM category to predict the prognosis. AJCC specified that this staging method could only be used in patients with the same histology.^72^ The current contradiction and controversy for sMPLC staging indicates that an appropriate classification system is still required to be established.

In our study, we explored the potential clinical prognostic factors. Consistently with NSCLC,^73^ the negative association between long‐term survival and elder age and male gender in sMPLC were identified in our study. Improved prognosis in female might be explained by the younger age distribution, more histologic subtype of adenocarcinoma, and hormonal factors.^74^ It is an undisputed fact that smoking causes lung cancer and negatively affects the prognosis. More than 60 carcinogens in smoking could directly damage and mutate DNA, while smoking cessation could promote the replenishment of bronchial epithelium with cells and regenerative capacity of the lungs.^75^ Owing to tobacco control, epidemiological studies reported that lung cancer mortality had considerably decreased in United States.^76^ In sMPLC, the negative effect of smoking on prognosis and the beneficial effect of smoking cessation also verified.^15^ FEV1 is an important preoperative evaluation item for surgical method selection and prediction of postoperative clinical outcomes,^77^ and patients with high FEV1 could receive more treatment ways and exhibit better prognosis. The role of FEV1 in sMPLC prognosis is more significant, since completed resection of tumors necessitate pulmonary function capacity.

T descriptor is a mandatory classification parameter of the TNM staging system and major prognostic factor in lung cancer. The size of the primary tumor and its invasion to nearby tissue determined T category.^69^ Consistently, the negative association between the largest tumor size and prognosis was observed in sMPLC, and the sum of tumors` sizes also had a significant impact on sMPLC prognosis. While whether the largest tumor size or sum of tumors` sizes should be chosen as a candidate for staging remains to be explored. Lymph node metastasis, another major prognostic factor in lung cancer,^78^ was also found to be an indicator of poor prognosis in sMPLC. No significant difference in prognosis was detected between different number of tumors in sMPLC, indicating lack of evidence supporting them as a staging classification parameter. The poor prognosis trend among sMPLC patients with different histology was observed in our study and it was consistent with a previous pooled analysis of six studies.^79^ Should histology type be considered in staging sMPLC remains unclear. No association between tumor location and prognosis of sMPLC in our study, while this pooled analysis demonstrated that patients with unilateral tumor location had a poorer prognosis than those with bilateral disease.^79^ This inconsistent might be attributed to the small sample size in the pooled analysis. However, both findings suggest that current staging classification according to the location of separate tumor nodules relative to the main tumor is not appropriate for sMPLC. Further studies are still needed to assess their prognostic effects since sample sizes and sparse studies included in our study.

Though surgical resection has been considered as the first‐choice treatments for sMPLC, the surgical method and its effect on prognosis remain controversial.^80^ According to the included studies, lobectomy, bi‐lobectomy, and pneumonectomy with lymphadenectomy were regarded as anatomical resection and at least one sublobar resection involved was treated as limited resection. A meta‐analysis showed that limited resection has a slightly poor trend of OS in sMPLC by comparison of anatomical resection, without a statistical significance.^81^ While in our study, though most of our included studies^16^, ^37^, ^43^, ^46^, ^52^ found no significant difference in prognosis, the pooled estimate from nine studies indicated that patients received anatomical resection has a significantly better prognosis than limited resection. One study focusing on both synchronous and metachronous MPLC patients^53^ also confirmed the better OS advantage of anatomical resection than sublobar resection. However, anatomical resection for all tumor nodules is impractical for patients with limited cardiorespiratory status, and sublobar resection is also a valid option. While it also demonstrated that anatomical segmentectomy could confer a comparable survival benefit with lobectomy and a better prognosis than nonanatomical resections and.^53^ In another study, sublobar resection for at least one lesion is advised for patients with sMPLC to preserve cardiorespiratory status.^82^ We also verified that pneumonectomy is an independent factor of poor prognosis, which was consistent with most studies.^25^, ^26^, ^36^, ^40^, ^53^, ^80^, ^81^ Additionally, pneumonectomy is associated with a greater operative risk than lobectomy and sublobar resection, and poor pulmonary function induced by pneumonectomy also facilitates a poor prognosis. However, pneumonectomy, as a bailout procedure, is inevitable even in stage I single lung cancer for intraoperative safe dissection and tumor mass, large lymph nodes, or cancerous mass near to hilum.^83^ Considering the cardiorespiratory status, operative risk, postoperative pulmonary function, and long‐term prognosis, our finding supports that anatomical resection should be preferred for sMPLC with adequate pulmonary reserve and pneumonectomy should be always avoided unless absolutely inevitable. For patients with limited pulmonary function, lobectomy with sublobar resection or only sublobar resections are also valid options, and anatomical segmentectomy is preferred in sublobar resection.

Limitations should be addressed. First, meta‐analysis for survival rates was based on the presupposition of no loss to follow‐up. However, we also used the effect size of HR to analyze prognosis and reached consistent results. Second, substantial heterogeneity and publication bias were observed in our meta‐analysis. High heterogeneity and publication bias are frequent in meta‐analysis for proportion; thus, subgroup analysis and sensitivity analyses were performed to confirm the stability of the pooled estimates and explore the source of heterogeneity. Though the substantial heterogeneity and publication bias could not be explained by the variables inspected, subgroup analysis, sensitivity analyses, and additional analysis confirmed the stability of pooled results. The heterogeneity and publication bias might be associated with study populations, diagnostic equipment, and study period. Third, though 52 studies included, the overall sample size is still small and effect of clinical parameters on prognosis still remains to be explored.

## 5 CONCLUSION

There are increasing trends of proportion of sMPLC in lung cancer, with a summary estimate of 2.0%. The 5‐year survival rate was 44.9%, and postoperative mortality was 1.4% with a decreasing trend over time. Lobectomy should be preferred for sMPLC, while pneumonectomy should be always avoided. Additionally, lobectomy with sublobar resection or only sublobar resections are also valid options, especially for patients with limited pulmonary function. Age, gender, FEV1, smoking, tumor size, and lymph node status are important prognostic factors for patients with sMPLC after surgery. Considering the heterogeneity and publication bias, the findings should be treated with caution. Further studies are needed to further analyze the clinical prognostic factors to establish a well‐predictive staging system for prognosis.

## CONFLICT OF INTEREST

The authors declare no competing interests.

## ETHICAL APPROVAL

Since all analyses are based on previous published studies, thus, no ethical approval and patient consent are required.

## Supporting information

Data S1

## Data Availability

The data used to support the findings of this study are all available in the Data S1.
