# Supplementary material for: Characteristics and prognosis of synchronous multiple primary lung cancer after surgical treatment: A systematic review and meta‐analysis of current evidence
Source: Cancer Med. 2020 Dec 10;10(2):507–20. doi: 10.1002/cam4.3614 (PMC7877344; doi:10.1002/cam4.3614)

**Contents of Supplement Data**

**Appendix 1 Search strategies**

**Appendix 2 Baseline Characteristic**

**eTable 1** Location of tumors in included studies

**Appendix 3 Modified Newcastle-Ottawa quality scale**

**Appendix 4 Overall outcome of the included studies**

**eTable 2** Outcome data of postoperative morality and 1-, 2-, 3-, 4-, and 5-year survival rate

**Appendix 5 Outcome data of 5-year survival rate according to various clinical parameters**

**eTable 3** Clinical parameters: age

**eTable 4** Clinical parameters: gender

**eTable 5** Clinical parameters: smoking status

**eTable 6** Clinical parameters: FEV1

**eTable 7** Clinical parameters: number of

**eTable 8** Clinical parameters: tumor

**eTable 9** Clinical parameters: tumor size

**eTable 10** Clinical parameters: surgery methods

**eTable 11** Clinical parameters: tumor histology

**eTable 12** Clinical parameters: lymph node metastasis status

**eTable 13** Clinical parameters: postoperative adjunctive therapy

**Appendix 6 Outcome data of OS (HRs and 95%CIs) according to various clinical parameters**

**eTable 14** Clinical parameters: age

**eTable 15** Clinical parameters: gender

**eTable 16** Clinical parameters: smoking status

**eTable 17** Clinical parameters: FEV1

**eTable 18** Clinical parameters: number of tumors

**eTable 19** Clinical parameters: tumor location

**eTable 20** Clinical parameters: tumor size

**eTable 21** Clinical parameters: surgery methods

**eTable 22** Clinical parameters: tumor histology

**eTable 23** Clinical parameters: lymph node metastasis status

**eTable 24** Clinical parameters: postoperative adjunctive therapy

**Appendix 7 eTable 25: Quality assessment results of included studies**

**Appendix 8 Forest chart for characteristics of synchronous MPLC**

**eFigure 1** Characteristics: age

**eFigure 2** Characteristics: gender ratio

**eFigure 3** Characteristics: the proportion of more than 2 tumors

**eFigure 4** Characteristics: the proportion of tumors located in unilateral lung

**eFigure 5** Characteristics: the proportion of tumors with identical histology

**Appendix 9 Forest chart for the proportion of sMPLC in lung cancer**

**eFigure 6** Forest chart of meta-analysis for the proportion of sMPLC in lung caner

**eFigure 7** Forest chart of subgroup analysis for the proportion of sMPLC according to publication year

**eFigure 8** Forest chart of subgroup analysis for the proportion of sMPLC according to study quality

**eFigure 9** Sensitivity analysis for the proportion of sMPLC in lung cancer

**Appendix 10 Forest chart for postoperative mortality**

**eFigure 10** Forest chart of meta-analysis for postoperative mortality

**eFigure 11** Forest chart of subgroup analysis for postoperative mortality according to publication year

**eFigure 12** Forest chart of subgroup analysis for the postoperative mortality according to study quality

**eFigure 13** Sensitivity analysis for the postoperative mortality

**Appendix 11 Forest chart for the survival rate of sMPLC**

**eFigure 14** Forest chart of meta-analysis for 1-year survival rate

**eFigure 15** Forest chart of meta-analysis for 2-year survival rate

**eFigure 16** Forest chart of meta-analysis for 3-year survival rate

**eFigure 17** Forest chart of meta-analysis for 5-year survival rate

**eFigure 18** Forest chart of subgroup analysis for 5-year survival rate according to publication year

**eFigure 19** Forest chart of subgroup analysis for 5-year survival rate according to study quality

**eFigure 20** Sensitivity analysis for 5-year survival rate

**Appendix 12 Effect of various clinical parameters on 5-year survival rate**

**eFigure 21** Clinical parameters: age

**eFigure 22** Clinical parameters: gender

**eFigure 23** Clinical parameters: smoking status

**eFigure 24** Clinical parameters: FEV1

**eFigure 25** Clinical parameters: number of tumors

**eFigure 26** Clinical parameters: tumor location

**eFigure 27** Clinical parameters: tumor size

**eFigure 28** Clinical parameters: surgery methods

**eFigure 29** Clinical parameters: tumor histology status

**eFigure 30** Clinical parameters: lymph node metastasis

**eFigure 31** Clinical parameters: postoperative adjunctive therapy

**Appendix 13 Effect of various clinical parameters on overall survival**

**eFigure 32** Clinical parameters: age

**eFigure 33** Clinical parameters: gender

**eFigure 34** Clinical parameters: smoking status

**eFigure 35** Clinical parameters: FEV1

**eFigure 36** Clinical parameters: number of tumors

**eFigure 37** Clinical parameters: tumor location

**eFigure 38** Clinical parameters: tumor size

**eFigure 39** Clinical parameters: surgery methods

**eFigure 40** Clinical parameters: tumor histology

**eFigure 41** Clinical parameters: lymph node metastasis

**eFigure 42** Clinical parameters: postoperative adjunctive therapy

**Appendix 14 Publication bias**

**eFigure 43** Publication bias for sMPLC

**eFigure 44** Publication bias for postoperative mortality

**eFigure 45** Publication bias for 5-years survival rate

**Appendix 1: Search strategies**

#1 "lung cancer"[All Fields]

#2 "lung carcinoma"[All Fields]

#3 "lung cancers"[All Fields]

#4 "Lung Neoplasms"[MeSH Terms]

#5 OR/ (#1-4)

#6 "surgery"[Subheading]

#7 "surgical procedures, operative"[MeSH Terms]

#8 "operative surgical procedures"[All Fields]

#9 surgery"[All Fields]

#10 lobectomy [All Fields]

#11 "pneumonectomy"[MeSH Terms]

#12 "pneumonectomy"[All Fields]

#13 resection [All Fields]

#14 OR (#6-13)

#15 "survival"[MeSH Terms]

#16 "survival"[All Fields]

#17 "mortality"[MeSH Terms]

#18 "mortality"[All Fields]

#19 "prognosis"[MeSH Terms]

#20 "prognosis"[All Fields]

#21 OR (#14-19)

#22 synchronous [Title/Abstract]

#23 multiple [Title/Abstract]

#24 OR (#21-23)

#25 #5 AND #14 AND #21 AND #24

**Appendix 2 Baseline Characteristic**

**eTable 1 Characteristic of included studies**

| **Study ID** | **Tumor location** |
| --- | --- |
| Adebonojo 1997 | Unilateral (10), bilateral (5) |
| Andriolo 2012 | NA |
| Angeletti 1995 | Unilateral (10), bilateral (8) |
| Antakli 1995 | Same lobe (4), unilateral different lobe (12), bilateral (10) |
| Aziz 2002 | Unilateral (6), bilateral (4) |
| Bae 2012 | Unilateral (12), bilateral (7) |
| Chang 2007 | Same lobe (55), unilateral different lobe (26), bilateral (11) |
| Chen 2015 | Same lobe (27), unilateral different lobe (56), bilateral (20) |
| Cheng 2017 | NA |
| De Leyn 2008 | Bilateral (36) |
| Deschamps 1990 | NA |
| Fabian 2011 | Unilateral (23), bilateral (44) |
| Feng 2005 | Same lobe (8), unilateral different lobe (16), bilateral (7) |
| Finley 2010 | Same lobe (27), unilateral different lobe (78), bilateral (70) |
| Guo 2017 | Same lobe (132), unilateral different lobe (128), bilateral (97) |
| Hardavella 2018 | NA |
| Hsu 2016 | Same lobe (0), unilateral different lobe (18), bilateral (17) |
| Ishikawa2014 | Same lobe (31), unilateral different lobe (36), bilateral (26) |
| Jung 2011 | Same lobe (8), unilateral different lobe (15), bilateral (9) |
| Kocaturk 2011 | Unilateral (14), bilateral (12) |
| Li 2011 | Same lobe (12), unilateral different lobe (35), bilateral (25) |
| Li 2017 | Same lobe (5); unilateral different lobe (8); bilateral (23) |
| Lin 2014 | Same lobe (23), unilateral different lobe (20), bilateral (21) |
| Liu 2016 | Unilateral (86), bilateral (36) |
| Liu 2017 | NA |
| lv 2018 | NA |
| Mun2007 | NA |
| Okada 1998 | Unilateral (21), bilateral (7) |
| Peng 2017 | Unilateral (29), bilateral (14) |
| Pommier 1996 | NA |
| Rea 2001 | Same lobe (0), Unilateral different lobes (9), bilateral (10) |
| Ribet 1995 | Unilateral (9), bilateral (15) |
| Riquet 2008 | Same lobe (57), unilateral different lobe (51), bilateral (10) |
| Rosengart 1991 | Same lobe (12), unilateral different lobe (13), bilateral (8) |
| Rostad 2008 | Same lobe (48), unilateral different lobe (42), bilateral (4) |
| Shah 2012 | Bilateral (47) |
| Takamochi 2012 | Same lobe (13), different lobe (18) |
| Tanvetyanon 2010 | Unilateral different lobe (57), bilateral (59) |
| Trousse 2007 | Same lobe (63), unilateral different lobe (28), bilateral (34) |
| Tsunezuka 2004 | Bilateral 19 |
| Tung 2003 | Unilateral different lobes (20) |
| van Rens 2000 | Same lobe (16), unilateral different lobe (26), bilateral (43) |
| Vansteenkiste 2001 | Same lobe (11), unilateral different lobe (17), bilateral (7) |
| Verhagen 1994 | Same lobe (1), unilateral different lobe (9), bilateral (5) |
| Voltolini 2010 | Same lobe (0), Unilateral different lobe (15), bilateral (28) |
| Wang 2001 | Same lobe (3), unilateral different lobe (6), bilateral (3) |
| Wang 2008 | same lobe (10), unilateral different lobe (6), bilateral (0) |
| Wu 1987 | Unilateral (9), bilateral (1) |
| Xiao 2017 | Unilateral (28), bilateral (24) |
| Yu 2013 | Same lobe (39), unilateral different lobe (46), bilateral (12) |
| Zhang 2016 | Same lobe (55), unilateral different lobe (136), bilateral (94) |
| Zuin 2013 | Unilateral (14), bilateral (9) |
| SMPLC=synchronous multiple primary lung cancer; LC=lung cancer; NA=Not available. | |

**Appendix 3: Modified Newcastle-Ottawa quality scale**

**(1) Sample representativeness:**

1 point: Population with sMPLC selected from patients receiving surgery.

0 points: sMPLC selection not reported.

**(2) Ascertainment of sMPLC?**

1 point: Definition of sMPLC described in the study.

0 points: Definition of sMPLC not reported.

**(3) Sample size:**

1 point: Sample size was greater than or equal to 30 participants.

0 points: Sample size was less than 30 participants.

**(4) Assessment of outcomes (postoperative mortality and survival)**

1 point: according to medical records or follow-up.

0 points: not reported.

**(5) Enough long follow-up:**

1 point: follow-up longer than or equal to 5 years.

0 points: follow-up less than 5 years.

**(6) Quality of descriptive statistics reporting:**

1 point: The study reported proper statistics to describe the patients’ characteristics (e.g. age, gender, tumor location, number of tumors, identical histology rate) and outcomes (postoperative mortality and 5-year survival rate)

0 points: The study did not report descriptive statistics, incompletely reported descriptive statistics, or did not report measures of dispersion.

**(7) Clinical parameter analysis on the prognosis of sMPLC.**

1 point: The study reported the estimated effect of clinical parameters on the sMPLC prognosis.

0 points: The study did not explore the effect of clinical parameters on the sMPLC prognosis.

**Appendix 4 Overall outcome of the included studies**

**eTable 2**: Outcome data of postoperative morality and 1-, 2-, 3-, and 5-year survival rate

| **Study ID** | **Postoperative mortality** | **Patient for prognosis analysis** | **1-year survival rate** | **2-year survival rate** | **3-year survival rate** | **5-year survival rate** |
| --- | --- | --- | --- | --- | --- | --- |
| Adebonojo 1997 | 0/15 | 15 | 86.7% | 73.2% | 58% | 0 |
| Andriolo 2012 | NA | 13 | NA | NA | NA | 50% |
| Angeletti 1995 | 0/18 | 18 | 81.2% | 69.0% | 61.6% | 46.2% |
| Antakli 1995 | NA | 26 | 72.5 | 12.5 | 12.5 | 12.5% |
| Aziz 2002 | 0/10 | 10 | 50% | 40% | 40% | 10% |
| Bae 2012 | 2/19 | 19 | 79.0% | 73.2% | 60.0% | 51.4% |
| Chang 2007 | 1/92 | 92 | NA | NA | NA | 35.3% |
| Chen 2015 | 0/103 | 103 | 100% | 100% | 87.30% | 68.60% |
| Cheng 2017 | 0/45 | 45 | 100% | 93.40% | 88.90% | 72% |
| De Leyn 2008 | 1/36 | 36 | NA | 73.1% | NA | 38.1% |
| Deschamps 1990 | 2/36 | 36 | NA | NA | NA | 15.70% |
| Fabian 2011 | 2/67 | 67 | 91.1% | 74% | 64% | 53% |
| Feng 2005 | 0/31 | 31 | 52% | NA | 29% | 20% |
| Finley 2010 | 2/175 | 175 | 87.9% | 75.3% | 64% | 51.8% |
| Guo 2017 | 0/357 | 357 | NA | NA | 91.93% | 84.37% |
| Hardavella 2018 | 0/8 | 8 | 100% | NA | NA | NA |
| Hsu 2016 | 0/35 | 35 | NA | NA | 91.5% | 75% |
| Ishikawa2014 | 0/93 | 93 | 98.1% | 95.8% | 93.6% | 87.0% |
| Jung 2011 | 3/32 | 32 | 84.0% | 80% | 69.6% | 60.9% |
| Kocaturk 2011 | 2/26 | 26 | 88.1% | 68.8% | 62.0% | 49.7% |
| Li 2011 | 0/72 | 72 | 81.20% | 70.70% | 53.70% | 43.00% |
| Li 2017 | 0/36 | 36 | 97.05% | 97.05% | 97.05% | 86.10% |
| Lin 2014 | 0/64 | 64 | NA | NA | NA | 95.8% |
| Liu 2016 | 4/122 | 122 | NA | NA | NA | 40.5% |
| Liu 2017 | NA | 438 | NA | NA | NA | 59.48% |
| lv 2018 | NA | 48 | 95.8%% | 89.70% | 89.7%% | NA |
| Mun2007 | 0/19 | 19 | NA | NA | 94.7% | 75.8% |
| Okada 1998 | 0/28 | 28 | 83.7% | 77.3% | 72.8% | 70.3% |
| Peng 2017 | 0/43 | 43 | 97.00% | NA | 76.70% | NA |
| Pommier 1996 | 0/27 | 27 | NA | NA | NA | 24% |
| Rea 2001 | 1/19 | 19 | 100% | 68.5% | 49.8% | 20% |
| Ribet 1995 | 1/24 | 15 | 86.7% | 46.7% | 6.7% | 0% |
| Riquet 2008 | 6/118 | 118 | 66.6% | 53.5% | 40.1% | 26% |
| Rosengart 1991 | NA | 33 | 81.6% | 68.3% | 54.0% | 44% |
| Rostad 2008 | 8/94 | 94 | NA | NA | NA | 27.6% |
| Shah 2012 | 1/47 | 47 | 69.6% | 51.6% | 35% | 29% |
| Takamochi 2012 | NA | 31 | 96.5% | 86.6% | 82.1% | 77.3% |
| Tanvetyanon 2010 | 5/116 | 116 | NA | NA | NA | NA |
| Trousse 2007 | 6/125 | 125 | 76.5% | 61.6% | 48.4% | 34% |
| Tsunezuka 2004 | NA | 19 | 94.5% | 88.6% | 80.8% | 69% |
| Tung 2003 | NA | 20 | 60.00% | 39.30% | 28.10% | 28.10% |
| van Rens 2000 | 12/73 | 73 | 71.6% | 51.7% | 34.6% | 19% |
| Vansteenkiste 2001 | 3/35 | 35 | 68.4% | 54% | 48.5% | 33% |
| Verhagen 1994 | 3/15 | 15 | 66% | 33.1% | 26% | 15% |
| Voltolini 2010 | 3/43 | 43 | 86.1% | 58.5% | 41% | 34% |
| Wang 2001 | 0/12 | 12 | 80% | NA | 77.70% | 14.30% |
| Wang 2008 | 0/16 | 16 | NA | NA | NA | 16.90% |
| Wu 1987 | 0/10 | 10 | 70% | 48% | 48% | 35% |
| Xiao 2017 | 0/52 | 52 | 96.10% | 90.20% | 75.50% | 40.60% |
| Yu 2013 | 0/97 | 97 | 96.7% | 93.1% | 84.8% | 69.6% |
| Zhang 2016 | NA | 285 | NA | NA | NA | 77.6%, |
| Zuin 2013 | NA | 23 | 91.4% | 64.2% | 55.0% | 40% |

NA, not available

**Appendix 5 Outcome data of 5-year survival rate according to various clinical parameters**

**eTable 3**: Outcome data of 5-year survival rate according to age

| **Study ID** | **Comparison** | **Subgroup** | **N** | **5-year survival rate** |
| --- | --- | --- | --- | --- |
| Hsu 2016 | ≥65yrs *vs.* <65yrs | Older | 20 | 62.2% |
| Hsu 2016 | ≥65yrs *vs.* <65yrs | Young | 15 | 88.9% |
| Ishikawa2014 | ≥70yrs *vs.* <70yrs | Older | 40 | 92% |
| Ishikawa2014 | ≥70yrs *vs.* <70yrs | Young | 53 | 83.8% |
| Jung 2011 | ≥65yrs *vs.* <65yrs | Older | 17 | 36% |
| Jung 2011 | ≥65yrs *vs.* <65yrs | Young | 15 | 85.7% |
| Liu 2016 | >60yrs *vs.* <=60yrs | Older | 57 | 45.4% |
| Liu 2016 | >60yrs *vs.* <=60yrs | Young | 65 | 38.8% |
| Takamochi 2012 | >65 *vs.*<=65yrs | Older | 19 | 75.8% |
| Takamochi 2012 | >65 *vs.*<=65yrs | Young | 12 | 80.2% |
| Voltolini 2010 | ≥67yrs *vs.* <67yrs | Older | 22 | 36.6% |
| Voltolini 2010 | ≥67yrs *vs.* <67yrs | Young | 21 | 27.6% |
| Yu 2013 | ≥65yrs *vs.* <65yrs | Older | 53 | 64% |
| Yu 2013 | ≥65yrs *vs.* <65yrs | Young | 44 | 77.4% |

**eTable 4**: Outcome data of 5-year survival rate according to gender

| **Study ID** | **Subgroup** | **N** | **5-year survival rate** |
| --- | --- | --- | --- |
| Fabian 2011 | Male | 30 | 53% |
| Fabian 2011 | Female | 37 | 50% |
| Hsu 2016 | Male | 11 | 57.1% |
| Hsu 2016 | Female | 24 | 85.1% |
| Ishikawa 2014 | Male | 36 | 77.4% |
| Ishikawa 2014 | Female | 57 | 93.1% |
| Jung 2011 | Male | 22 | 44.6% |
| Jung 2011 | Female | 10 | 100% |
| Kocaturk 2011 | Male | 26 | 49.7% |
| Liu 2016 | Male | 54 | 46.6% |
| Liu 2016 | Female | 68 | 39.2% |
| Takamochi 2012 | Male | 14 | 58.6% |
| Takamochi 2012 | Female | 17 | 94.1% |
| Voltolini 2010 | Male | 40 | 33.4% |
| Voltolini 2010 | Female | 3 | 50% |
| Yu 2013 | Male | 42 | 62.2% |
| Yu 2013 | Female | 55 | 75.5% |

**eTable 5:** Outcome data of 5-year survival rate according to smoking status

| **Study ID** | **Subgroup** | **N** | **5-year survival rate** |
| --- | --- | --- | --- |
| Guo 2017 | Never smoker | 296 | 90.7% |
| Guo 2017 | Smoker | 61 | 63.1% |
| Hsu 2016 | Never smoker | 27 | 71.8% |
| Hsu 2016 | Smoker | 8 | 85.7% |
| Ishikawa 2014 | Never smoker | 60 | 90% |
| Ishikawa 2014 | Smoker | 33 | 81.8% |
| Jung 2011 | Never smoker | 10 | 100% |
| Jung 2011 | Smoker | 22 | 44.6% |
| Liu 2016 | Never smoker | 76 | 55.6% |
| Liu 2016 | Smoker | 46 | 30.8% |
| Takamochi 2012 | Never smoker | 13 | 92.3% |
| Takamochi 2012 | Smoker | 18 | 66.5% |
| Yu 2013 | never smoker | 58 | 77.8% |
| Yu 2013 | smoker | 39 | 58.5% |

**eTable 6**: Outcome data of 5-year survival rate according to FEV1

| **Study ID** | **FEV1** | **Subgroup** | **N** | **5-year survival rate** |
| --- | --- | --- | --- | --- |
| Bae 2012 | ＜80% | Low | 7 | 35.7% |
| Bae 2012 | ≥80% | High | 12 | 48.1% |
| Hsu 2016 | ＜80% | Low | 12 | 57.1% |
| Hsu 2016 | ≥80% | High | 23 | 83.6% |
| Jung 2011 | ＜70% | Low | 8 | 25% |
| Jung 2011 | ≥70% | High | 24 | 76% |
| Yu 2013 | ＜80% | Low | 18 | 66.9% |
| Yu 2013 | ≥80% | High | 79 | 70.7% |
| Xiao 2017 | ＜80% | Low | 17 | 9.7% |
| Xiao 2017 | >=80 | High | 35 | 47.6% |

**eTable 7**: Outcome data of 5-year survival rate according to the number of tumors

| **Study ID** | **Subgroup** | **N** | **5-year survival rate** |
| --- | --- | --- | --- |
| Ishikawa 2014 | <3 | 71 | 90.7% |
| Ishikawa 2014 | >=3 | 22 | 71.1% |
| Liu 2016 | <3 | 101 | 43.9% |
| Liu 2016 | >=3 | 21 | 36.8% |
| Rostad 2008 | <3 | 67 | 27.1% |
| Rostad 2008 | >=3 | 27 | 29% |

**eTable 8**: Outcome data of 5-year survival rate according to tumor location

| **Study ID** | **Location** | **Subgroup** | **N** | **5-year survival rate** |
| --- | --- | --- | --- | --- |
| Bae 2012 | Unilateral (same+different) | Unilateral | 12 | 64.2% |
| Bae 2012 | Bilateral | Bilateral | 7 | 22.2% |
| Fabian 2011 | Same lobe | Unilateral | 5 | 78% |
| Fabian 2011 | Ipsilateral different lobe | Unilateral | 18 | 43% |
| Fabian 2011 | Bilateral | Bilateral | 44 | 54% |
| Hsu 2016 | Ipsilateral different lobe | Unilateral | 18 | 76.2% |
| Hsu 2016 | Bilateral | Bilateral | 17 | 76.6% |
| Ishikawa 2014 | Unilateral (same+different) | Unilateral | 67 | 90.9 |
| Ishikawa 2014 | Bilateral | Bilateral | 26 | 76.9% |
| Jung 2011 | Same lobe | Unilateral | 8 | 50% |
| Jung 2011 | Ipsilateral different lobe | Unilateral | 15 | 62.2% |
| Jung 2011 | Bilateral | Bilateral | 9 | 57.1% |
| Kocaturk 2011 | Unilateral (same+different) | Unilateral | 14 | 40.6% |
| Kocaturk 2011 | Bilateral | Bilateral | 12 | 62.8% |
| Liu 2016 | Unilateral (same+different) | Unilateral | 86 | 41.5% |
| Liu 2016 | Bilateral | Bilateral | 36 | 36.8% |
| Riquet 2008 | Same lobe | Unilateral | 57 | 29.9% |
| Riquet 2008 | Ipsilateral different lobe | Unilateral | 51 | 15.6% |
| Trousse 2007 | Same lobe | Unilateral | 63 | 32.7% |
| Trousse 2007 | Ipsilateral different lobe | Unilateral | 28 | 24.3% |
| Trousse 2007 | Bilateral | Bilateral | 34 | 44.3% |
| Tsunezuka 2004 | Bilateral | Bilateral | 19 | 69% |
| Tung 2003 | Ipsilateral different lobe | Unilateral | 20 | 28.1% |
| van Rens 2000 | Same lobe | Unilateral | 16 | 29% |
| van Rens 2000 | Different lobe | Bilateral | 69 | 16% |
| Vansteenkiste 2001 | Same lobe | Unilateral | 11 | 23% |
| Vansteenkiste 2001 | Ipsilateral different lobe | Unilateral | 17 | 18% |
| Vansteenkiste 2001 | Bilateral | Bilateral | 7 | 43% |
| Voltolini 2010 | Unilateral (same+different) | Unilateral | 15 | 43% |
| Voltolini 2010 | Bilateral | Bilateral | 28 | 27% |
| Wang 2008 | Same lobe | Unilateral | 9 | 30.4% |
| Yu 2013 | Same lobe | Unilateral | 39 | 60.6% |
| Yu 2013 | Ipsilateral different lobe | Unilateral | 46 | 76.3% |
| Yu 2013 | Bilateral | Bilateral | 12 | 68.8% |

**eTable 9**: Outcome data of 5-year survival rate according to tumor size

| **Study ID** | **Subgroup** | **N** | **5-year survival rate** |
| --- | --- | --- | --- |
| Hsu 2016 | Largest tumor size<=3cm | 17 | 100% |
| Hsu 2016 | Largest tumor size>3cm | 18 | 62.1% |
| Ishikawa 2014 | Largest tumor size<=3cm | 72 | 84.5% |
| Ishikawa 2014 | Largest tumor size>3cm | 21 | 95.2% |
| Jung 2011 | Largest tumor size<=3cm | 19 | 84.2% |
| Jung 2011 | Largest tumor size>3cm | 13 | 24.4% |
| Liu 2016 | Largest tumor size<=3cm | 73 | 41.2% |
| Liu 2016 | Largest tumor size>3cm | 49 | 38.5% |
| Yu 2013 | Largest tumor size<=3cm | 29 | 85.9% |
| Yu 2013 | Largest tumor size>3cm | 68 | 62.3% |
| Xiao 2017 | Largest tumor size<=3cm | 27 | 50.5% |
| Xiao 2017 | Largest tumor size>3cm | 25 | 16.8% |
| Guo 2017 | Largest tumor size<=2cm | 244 | 96.8% |
| Guo 2017 | Largest tumor size>2cm | 113 | 68.5% |

**eTable 10**: Outcome data of 5-year survival rate according to surgery methods

| **Study ID** | **Surgery method** | **Subgroup1** | **Subgroup2** | **N** | **5-year survival rate** |
| --- | --- | --- | --- | --- | --- |
| Bae 2012 | Pneumonectomy | Pneumonectomy | Extended | 6 | 50% |
| Bae 2012 | Non-pneumonectomy | Limited | Limited | 13 | 47.9% |
| De Leyn 2008 | Limited resection | Limited | Limited | 26 | 36.7% |
| De Leyn 2008 | Bilateral lobectomy | Anatomical | Extended | 10 | 40% |
| Ishikawa 2014 | Segmentectomy | Limited | Limited | 18 | 82.1% |
| Ishikawa 2014 | Wedge resection | Limited | Limited | 36 | 80.4% |
| Ishikawa 2014 | Lobectomy | Anatomical | Extended | 39 | 92.5% |
| Jung 2011 (1) | Pneumonectomy | pneumonectomy | Extended | 5 | 30% |
| Jung 2011 (1) | Non-pneumonectomy | Limited | Limited | 27 | 73% |
| Jung 2011 (2) | Limited resection | Limited | Limited | 17 | 79.4% |
| Jung 2011 (2) | No limited resection | Anatomical | Extended | 15 | 51.2% |
| Kocaturk 2011 | Pneumonectomy | pneumonectomy | Extended | 11 | 27% |
| Kocaturk 2011 | Non-pneumonectomy | Limited | Limited | 15 | 71.1% |
| Liu 2016 | Pneumonectomy | pneumonectomy | Extended | 14 | 10.4% |
| Liu 2016 | Limited resection | Limited | Limited | 26 | 42.7% |
| Liu 2016 | Lobectomy and limited resection | Limited | Limited | 48 | 50.7% |
| Liu 2016 | Lobectomy | Anatomical | Extended | 34 | 38.8% |
| Liu 2017 | Pneumonectomy | Pneumonectomy | Extended | 14 | 45.9% |
| Liu 2017 | Sublobar resection | Limited | Limited | 233 | 53.8% |
| Liu 2017 | Lobectomy | Anatomical | Extended | 174 | 70.5% |
| Pommier 1996 | Incompletely | Limited | Limited | 13 | 0% |
| Pommier 1996 | Resected completely | Anatomical | Extended | 14 | 45% |
| Vansteenkiste 2001 | Limited resection | Limited | Limited | 8 | 37% |
| Vansteenkiste 2001 | Non-limited resection | Anatomical | Extended | 27 | 27% |
| Voltolini 2010 | Sublobar resection | Limited | Limited | 28 | 29% |
| Voltolini 2010 | Lobar resection | Anatomical | Extended | 15 | 42% |
| Xiao 2017 | Limited | Limited | Limited | 43 | 41.1% |
| Xiao 2017 | Extended | Anatomical | Extended | 9 | 8.5% |
| Yu 2013 | Sublobar+sublobar | Limited | Limited | 13 | 64.7% |
| Yu 2013 | Lobect+lobect/sublobar | Limited | Limited | 45 | 79.7% |
| Yu 2013 | Lobectomy | Anatomical | Extended | 39 | 60.6% |

**eTable 11**: Outcome data of 5-year survival rate according to tumor histology

| **Study ID** | **Subgroup** | **N** | **5-year survival rate** |
| --- | --- | --- | --- |
| Angeletti 1995 | Same | 6 | 16.7% |
| Angeletti 1995 | Different | 12 | 0% |
| Bae 2012 | Same | 8 | 75% |
| Bae 2012 | Different | 11 | 20.8% |
| De Leyn 2008 | Same | 18 | 31.1% |
| De Leyn 2008 | Different | 18 | 45.5% |
| Jung 2011 | Same | 14 | 100% |
| Jung 2011 | Different | 18 | 35.6% |
| Kocaturk 2011 | Same | 17 | 25% |
| Kocaturk 2011 | Different | 9 | 78% |
| Liu 2016 | Same | 88 | 46.9% |
| Liu 2016 | Different | 34 | 24.8% |
| Liu 2017 | Same | 339 | 65% |
| Liu 2017 | Different | 99 | 41.7% |
| Pommier 1996 | Same | 15 | 33% |
| Pommier 1996 | Different | 12 | 17% |
| Rea 2001 | Same | 13 | 15% |
| Rea 2001 | Different | 6 | 33% |
| Riquet 2008 | Same | 60 | 32.5% |
| Riquet 2008 | Different | 58 | 20.2% |
| Rostad 2008 | Same | 85 | 29.2% |
| Rostad 2008 | Different | 9 | 12.7% |
| Shah 2012 | Same | 19 | 23% |
| Shah 2012 | Different | 17 | 40% |
| Vansteenkiste 2001 | Same | 30 | 31% |
| Vansteenkiste 2001 | Different | 5 | 20% |
| Voltolini 2010 | Same | 27 | 34% |
| Voltolini 2010 | Different | 16 | 33% |
| Yu 2013 | Same | 38 | 64.9% |
| Yu 2013 | Different | 59 | 74% |

**eTable 12**: Outcome data of 5-year survival rate according to lymph node metastasis

| **Study ID** | **Subgroup** | **N** | **5-year survival rate** |
| --- | --- | --- | --- |
| Chang 2007 | N0 | 50 | 52.5% |
| Chang 2007 | N1+ | 42 | 15.5% |
| Hsu 2016 | N0 | 32 | 73.6% |
| Hsu 2016 | N1+ | 3 | 100% |
| Ishikawa2014 | N0 | 75 | 93.4% |
| Ishikawa2014 | N1 | 10 | 75% |
| Ishikawa2014 | N2 | 8 | 41.7% |
| Jung 2011 | N0 | 25 | 64.5% |
| Jung 2011 | N1+ | 7 | 53.6% |
| Vansteenkiste 2001 | N0 | 20 | 33% |
| Vansteenkiste 2001 | N1 | 8 | 25% |
| Vansteenkiste 2001 | N2 | 7 | 17% |
| Voltolini 2010 | N0 | 25 | 57% |
| Voltolini 2010 | N1+ | 18 | 0% |
| Yu 2013 | N0 | 75 | 71.7% |
| Yu 2013 | N1+ | 22 | 59.7% |
| Tung 2003 | N0 | 6 | 66.7% |
| Tung 2003 | N1+ | 12 | 10.3% |
| Guo 2017 | N0 | 319 | 88.7% |
| Guo 2017 | N1+ | 38 | 54.8% |
| Takamochi 2012 | N0 | 24 | 81.3% |
| Takamochi 2012 | N1+ | 7 | 66.7% |

**eTable 13**: Outcome data of 5-year survival rate according to postoperative adjunctive therapy

| **Study ID** | **Subgroup** | **N** | **5-year survival rate** |
| --- | --- | --- | --- |
| Bae 2012 | Yes | 8 | 31.2% |
| Bae 2012 | No | 11 | 71.4% |
| Hsu 2016 | Yes | 24 | 71.5% |
| Hsu 2016 | No | 11 | 87.5% |
| Ishikawa 2014 | No | 12 | 50% |
| Ishikawa 2014 | Yes | 6 | 83.3% |
| Jung 2011 | No | 20 | 45% |
| Jung 2011 | Yes | 12 | 90% |
| Trousse 2007 | Yes | 58 | 47.2% |
| Trousse 2007 | No | 67 | 52.8% |
| Yu 2013 | No | 39 | 62.5% |
| Yu 2013 | Yes | 58 | 73.7% |

**Appendix 6 Outcome data of OS (HRs and 95%CIs) according to various clinical parameters**

**eTable 14**: Outcome data of OS according to clinical parameters: age

| **Study ID** | **Comparison** | **HRs with 95%CI** |
| --- | --- | --- |
| Guo 2017 | >60yrs *vs.* ≤60yrs | 2.951(1.222-7.126) |
| Rostad 2008 | >70yrs *vs.*≤70yrs | 1.91 (1.15-3.19) |
| Trousse-2007 | ≥60yrs *vs.* <60yrs | 1.89 (1.10-3.23) |
| Voltolini 2010 | ≥67yrs *vs.* <67yrs | 1.499 (0.466-4.831) |
| Zhang 2016 | ≥60yrs *vs.* <60yrs | 1.51 (0.72–3.15) |

**eTable 15**: Outcome data of OS according to clinical parameters: gender

| **Study ID** | **Comparison** | **HRs with 95%CI** |
| --- | --- | --- |
| Voltolini 2010 | Male *vs.* Female | 3.05 (0.327-28.477) |
| Zhang 2016 | Male *vs.* Female | 2.56 (1.16-5.68) |
| Trousse-2007 | Male *vs.* Female | 2.5 (1.15-5.42) |
| Rostad 2008 | Male *vs.* Female | 1.70 (1.01-2.86) |
| Tanvetyanon 2010 | Male *vs.* Female | 1.69 (1.01-2.86) |
| Guo 2017 | Male *vs.* Female | 5.09 (2.105-12.31) |
| Finley 2010 | Male *vs.* Female | 2.21 (1.45-3.38) |

**eTable 16**: Outcome data of OS according to clinical parameters: smoking status

| **Study ID** | **Comparison** | **HRs with 95%CI** |
| --- | --- | --- |
| Finley 2010 | Current smoker *vs.* Non-smoker | 1.85 (0.68 -5) |
| Guo 2017 | Smoker *vs.* Non-smoker | 4.771 (2.249-7.293) |
| Liu 2016 | Smoker *vs.* Non-smoker | 5.021 (3.007-22.332) |
| Voltolini 2010 | Smoker *vs.* Non-smoker | 1.959 (0.188-20.412) |
| Zhang 2016 | Light smoker *vs.* Non-smoker | 0.84 (0.11-6.42) |
| Zhang 2016 | Moderate smoker *vs.* Non-smoker | 2.63 (1.1-6.27) |
| Zhang 2016 | Heavy smoker *vs.* Non-smoker | 2.06 (0.87-4.93) |

**eTable 17**: Outcome data of OS according to clinical parameters: FEV1

| **Study ID** | **Comparison** | **HRs with 95%CI** |
| --- | --- | --- |
| Tanvetyanon 2010 | High *vs.* Low | 0.49 (0.26－0.94 |
| Trousse 2007 | High *vs.* Low | 0.95 (0.93－0.98) |
| Xiao 2017 | High *vs.* Low | 0.315 (0.118-0.847) |

**eTable 18**: Outcome data of OS according to clinical parameters: number of tumors

| **Study ID** | **Comparison** | **HRs with 95%CIs** |
| --- | --- | --- |
| Zhang 2016 | ≥3 *vs.* <3 | 0.7 (0.25-2.01) |
| Guo 2017 | ≥3 *vs.* <3 | 4.44 (0.98-20.41) |

**eTable 19**: Outcome data of OS to clinical parameters: tumor location

| **Study ID** | **Comparison** | **HRs with 95%CIs** |
| --- | --- | --- |
| Finley 2010 | Ipsilateral different *vs.* Same | 1.4 (0.73-2.7) |
| Finley 2010 | Bilateral *vs.* Same | 1.31 (0.68-2.51) |
| Guo 2017 | Ipsilateral different *vs.* Same | 2.55 (0.9-7.26) |
| Guo 2017 | Bilateral *vs.* Same | 1.55 (0.49-4.89) |
| Ishikawa2014 | Bilateral vs. Unilateral | 4.63 (1.148-18.666) |
| Tanvetyanon 2010 | Bilateral *vs.* Unilateral | 0.89 (0.37-2.19) |
| Tanvetyanon 2010 | Bilateral *vs.* Unilateral | 0.71 (0.4-1.25) |
| Voltolini 2010 | Bilateral *vs.* Unilateral | 1.675 (0.631-4.444) |
| Zhang 2016 | Bilateral *vs.* Unilateral | 0.62 (0.29-1.33) |
| Zhang 2016 | Bilateral *vs.* Same | 0.87 (0.21-3.66) |

**eTable 20**: Outcome data of OS according to clinical parameters: tumor size

| **Study ID** | **Comparison** | **HRs with 95%CIs** |
| --- | --- | --- |
| Finley 2010 | Largest tumor size (per cm increase) | 1.11 (1.01–1.23) |
| Guo 2017 (1) | >2cm *vs.* ≤ 2cm | 6.737 (2.579-10.895) |
| Guo 2017 (2) | sum of tumor size >3 cm *vs.* ≤ 3cm | 8.419 (2.497-28.394) |
| Hsu 2016 | >3cm *vs.* ≤ 3cm | 42.83 (0.04-46804) |
| Tanvetyanon 2010 (1) | largest tumor size (per cm increase) | 1.17 (1.06-1.3) |
| Tanvetyanon 2010 (2) | sum of tumor size (per cm increase) | 1.15 (1.05-1.26) |
| Xiao 2017 | >3cm *vs.* ≤ 3cm | 2.62 (1.15–5.98) |
| Zhang 2016 (1) | >2, ≤ 3cm *vs.* ≤ 2cm | 2.24 (0.70–7.13) |
| Zhang 2016 (2) | >3, ≤ 5cm *vs.* ≤ 2cm | 3.85 (1.22–12.09) |
| Zhang 2016 (3) | >5 *vs.* ≤ 2cm | 8.55 (2.40–30.54) |

**eTable 21**: Outcome data of OS according to clinical parameters: surgery methods

| **Study ID** | **Comparison** | **HRs with 95%CIs** |
| --- | --- | --- |
| Guo 2017 (1) | Limited *vs.* Anatomical | 1.253 (0.464-3.385) |
| Guo 2017 (2) | Limited *vs.* Anatomical | 2.079 (0.760-5.690) |
| Ishikawa2014 | Limited *vs.* Anatomical | 4.425 (1.054–18.580) |
| Kocaturk 2011 | Pneumonectomy *vs.* no | 1.68 (0.96-30.2) |
| Liu 2016 | Pneumonectomy *vs.* no | 5.623 (3.067-27.855) |
| Liu 2017 | Limited *vs.* Anatomical | 1.41 (1.07-1.89) |
| Liu 2017 | pneumonectomy *vs.* no | 1.91 (1.0-3.7) |
| Rostad 2008 | pneumonectomy *vs.* no | 1.96 (1.18-3.26) |
| Shah 2012 | Limited *vs.* Anatomical | 1.19 (0.52-2.78) |
| Tanvetyanon 2010 | Pneumonectomy *vs.* no | 1.36 (0.58-3.18) |
| Trousse 2007 | Pneumonectomy *vs.* no | 6.6 (3.34-13.1) |
| van Rens 2000 | Limited *vs.* Anatomical | 1.44 (0.86-2.4) |
| Voltolini 2010 | Limited *vs.* Anatomical | 1.048 (0.389-2.823) |
| Xiao 2017 | Limited vs. Anatomical | 0.32 (0.108-0.952) |
| Zhang 2016 (1) | Limited vs. Anatomical | 1 (0.44-2.3) |
| Zhang 2016 (2) | Limited vs. Anatomical | 1.22 (0.45-3.27) |

**eTable 22**: Outcome data of OS according to clinical parameters: tumor histology

| **Study ID** | **Comparison** | **HRs with 95%CIs** |
| --- | --- | --- |
| Finley 2010 | Different *vs.* same | 1.64 (0.99-2.74) |
| Kocaturk 2011 | Different *vs.* same | 0.38 (0.07-1.83) |
| Tanvetyanon 2010 | Different *vs.* same | 0.81 (0.47-1.41) |
| Voltolini 2010 | Different *vs.* same | 1.927 (0.688-5.405) |
| Guo 2017 | Different *vs.* same | 7.87 (3.41-18.18) |

**eTable 23**: Outcome data of OS according to clinical parameters: lymph node metastasis

| **Study ID** | **Comparison** | **HRs with 95%CIs** |
| --- | --- | --- |
| Chang 2007 | N1+ *vs.* N0 | 2.367 (1.196-4.682) |
| Finley 2010 | N1 *vs.* N0 | 1.96 (1.11-3.47) |
| Finley 2010 | N2 *vs.* N0 | 1.96 (1.08-3.53) |
| Guo 2017 | N1+ *vs.* N0 | 3.753 (2.229-5.277) |
| Ishikawa2014 | N1+ *vs.* N0 | 10.56 (2.142-52.076) |
| Liu 2016 | N1+ *vs.* N0 | 6.036 (4.614-94.917) |
| Tanvetyanon 2010 | N1+ *vs.* N0 | 1.42 (0.74-2.73) |
| Trousse 2007 | N1+ *vs.* N0 | 2.9 (1.2-6.9) |
| Voltolini 2010 | N1+ *vs.* N0 | 4.95 (1.83-13.33) |
| Xiao 2017 | N1+ *vs.* N0 | 1.71 (0.71-4.13) |
| Zhang 2016 | N1 *vs.* N0 | 2.33 (0.88-6.15) |
| Zhang 2016 | N2 *vs.* N0 | 5.48 (2.36-12.74) |

**eTable 24**: Outcome data of OS according to clinical parameters: postoperative adjunctive therapy

| **Study ID** | **Comparison** | **HRs with 95%CIs** |
| --- | --- | --- |
| Kocaturk 2011 | No *vs.* Yes | 1.8 (0.88-41.97) |
| Trousse-2007 | No *vs.* Yes | 1.8 (1.02-3.33) |
| Voltolini 2010 | No *vs.* Yes | 0.603 (0.195-1.862) |
| Zhang 2016 | No *vs.* Yes | 0.61 (0.3-1.23) |

**Appendix 7 eTable 25: Quality assessment results of included studies**

| **Study ID** | **Study type** | **Representativeness** | **Ascertainment of sMPLC** | **Sample Size >=30** | **Assessment of outcomes** | **Enough long follow-up** | **Quality of descriptive statistics reporting** | **Clinical parameter on the prognosis of sMPLC** | **Total score** |
| --- | --- | --- | --- | --- | --- | --- | --- | --- | --- |
| Adebonojo 1997 | longitudinal | 1 | 1 | 0 | 1 | 1 | 0 | 0 | 4 |
| Angeletti 1995 | longitudinal | 1 | 1 | 0 | 1 | 1 | 1 | 0 | 5 |
| Antakli 1995 | longitudinal | 1 | 1 | 0 | 1 | 1 | 0 | 0 | 4 |
| Aziz 2002 | longitudinal | 1 | 1 | 0 | 1 | 1 | 1 | 0 | 5 |
| Bae 2012 | longitudinal | 1 | 1 | 0 | 1 | 1 | 1 | 1 | 6 |
| Chang 2007 | longitudinal | 1 | 1 | 1 | 1 | 1 | 1 | 0 | 6 |
| De Leyn 2008 | longitudinal | 1 | 0 | 1 | 1 | 1 | 1 | 1 | 6 |
| Fabian 2011 | longitudinal | 0 | 1 | 1 | 1 | 1 | 1 | 1 | 6 |
| Feng 2005 | longitudinal | 1 | 1 | 1 | 1 | 1 | 1 | 0 | 6 |
| Finley 2010 | longitudinal | 0 | 1 | 1 | 1 | 1 | 1 | 1 | 6 |
| Hsu 2016 | longitudinal | 1 | 1 | 1 | 1 | 1 | 1 | 1 | 7 |
| Ishikawa2014 | longitudinal | 1 | 1 | 1 | 1 | 1 | 1 | 1 | 7 |
| Jung 2011 | longitudinal | 1 | 1 | 1 | 1 | 1 | 1 | 1 | 7 |
| Kocaturk 2011 | longitudinal | 1 | 1 | 0 | 1 | 1 | 1 | 1 | 6 |
| Lin 2014 | longitudinal | 1 | 0 | 1 | 1 | 1 | 1 | 0 | 5 |
| Liu 2016 | longitudinal | 1 | 1 | 1 | 1 | 1 | 1 | 1 | 7 |
| Mun2007 | longitudinal | 1 | 1 | 0 | 1 | 1 | 1 | 0 | 5 |
| Okada 1998 | longitudinal | 1 | 1 | 0 | 1 | 1 | 1 | 0 | 5 |
| Pommier 1996 | longitudinal | 1 | 1 | 0 | 1 | 1 | 1 | 1 | 6 |
| Rea 2001 | longitudinal | 0 | 1 | 0 | 1 | 1 | 0 | 1 | 4 |
| Ribet 1995 | longitudinal | 1 | 1 | 0 | 1 | 1 | 1 | 0 | 5 |
| Riquet 2008 | longitudinal | 1 | 1 | 1 | 1 | 1 | 1 | 1 | 7 |
| Rosengart 1991 | longitudinal | 0 | 1 | 1 | 1 | 1 | 1 | 0 | 5 |
| Rostad 2008 | longitudinal | 1 | 0 | 1 | 1 | 1 | 1 | 1 | 6 |
| Shah 2012 | longitudinal | 0 | 0 | 1 | 1 | 1 | 1 | 1 | 5 |
| Tanvetyanon 2010 | longitudinal | 1 | 1 | 1 | 1 | 0 | 1 | 1 | 6 |
| Trousse 2007 | longitudinal | 1 | 1 | 1 | 1 | 1 | 1 | 1 | 7 |
| Tsunezuka 2004 | longitudinal | 1 | 1 | 0 | 1 | 1 | 1 | 0 | 5 |
| van Rens 2000 | longitudinal | 1 | 1 | 1 | 1 | 1 | 1 | 1 | 7 |
| Vansteenkiste 2001 | longitudinal | 0 | 0 | 1 | 1 | 1 | 1 | 1 | 5 |
| Verhagen 1994 | longitudinal | 1 | 1 | 0 | 1 | 1 | 0 | 0 | 4 |
| Voltolini 2010 | longitudinal | 1 | 0 | 1 | 1 | 1 | 1 | 1 | 6 |
| Wu 1987 | longitudinal | 1 | 1 | 0 | 1 | 1 | 1 | 0 | 5 |
| Yu 2013 | longitudinal | 1 | 1 | 1 | 1 | 1 | 1 | 1 | 7 |
| Zhang 2016 | longitudinal | 0 | 1 | 1 | 1 | 1 | 1 | 1 | 6 |
| Zuin 2013 | longitudinal | 1 | 1 | 0 | 1 | 1 | 1 | 0 | 5 |
| Andriolo 2012 | longitudinal | 0 | 1 | 0 | 1 | 1 | 0 | 0 | 3 |
| Deschamps 1990 | longitudinal | 0 | 0 | 1 | 1 | 1 | 0 | 0 | 3 |
| Hardavella 2018 | longitudinal | 0 | 0 | 0 | 1 | 0 | 0 | 0 | 1 |
| Liu 2017 | longitudinal | 0 | 1 | 1 | 1 | 1 | 0 | 1 | 5 |
| Chen 2015 | longitudinal | 1 | 1 | 1 | 1 | 1 | 1 | 0 | 6 |
| Li 2011 | longitudinal | 0 | 1 | 1 | 1 | 1 | 1 | 0 | 5 |
| Wang 2001 | longitudinal | 1 | 1 | 0 | 1 | 1 | 1 | 0 | 5 |
| Wang 2008 | longitudinal | 1 | 1 | 0 | 1 | 1 | 1 | 1 | 6 |
| Tung 2003 | longitudinal | 1 | 0 | 0 | 1 | 1 | 1 | 1 | 5 |
| Cheng 2017 | longitudinal | 1 | 1 | 1 | 1 | 1 | 1 | 0 | 6 |
| Xiao 2017 | longitudinal | 1 | 1 | 1 | 1 | 1 | 1 | 1 | 7 |
| Guo 2017 | longitudinal | 1 | 1 | 1 | 1 | 1 | 1 | 1 | 7 |
| Peng 2017 | longitudinal | 0 | 1 | 1 | 1 | 0 | 1 | 0 | 4 |
| Li 2017 | longitudinal | 1 | 1 | 1 | 1 | 1 | 1 | 0 | 6 |
| lv 2018 | longitudinal | 1 | 1 | 1 | 1 | 0 | 1 | 0 | 5 |
| Takamochi 2012 | longitudinal | 1 | 1 | 1 | 1 | 1 | 1 | 1 | 7 |

**Appendix 8 Forest chart for characteristics of synchronous MPLC**


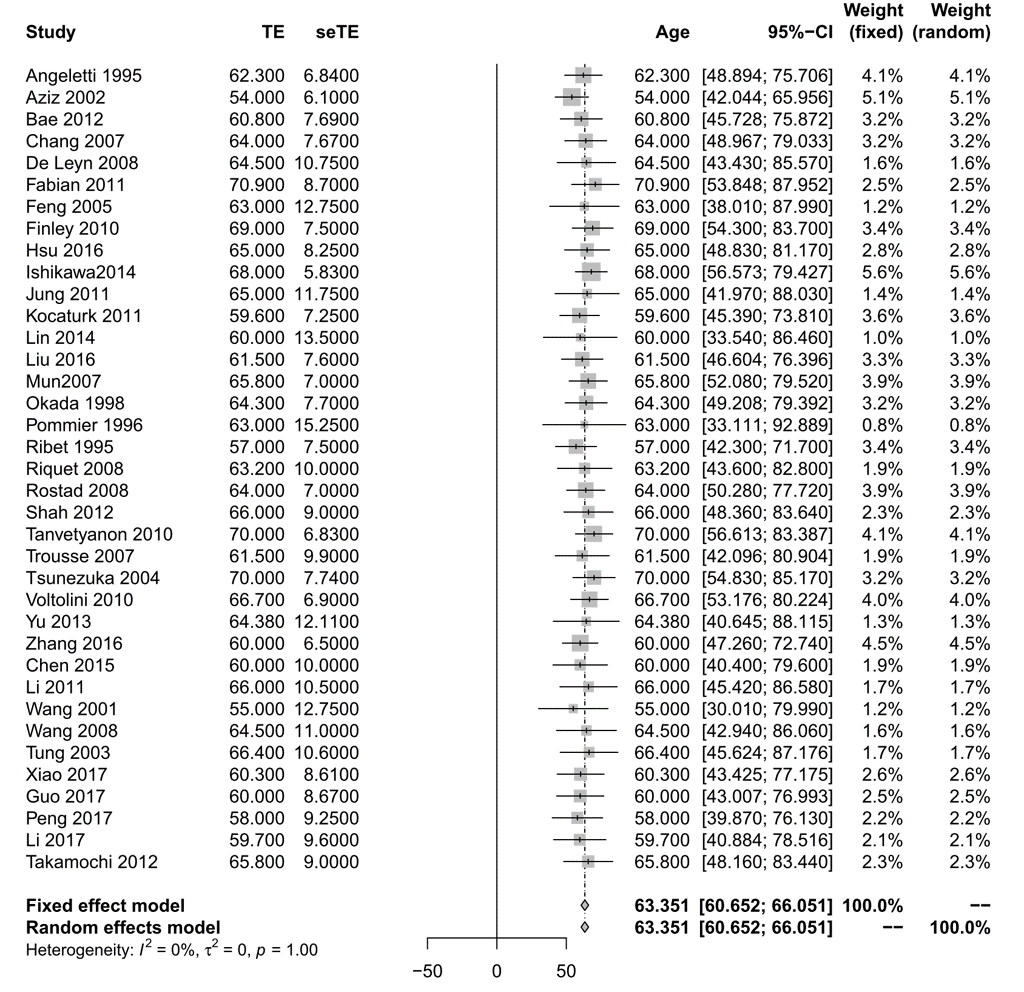
**eFigure 1** Forest chart of meta-analysis for characteristics of synchronous MPLC: age

Result from random-effects model is used.


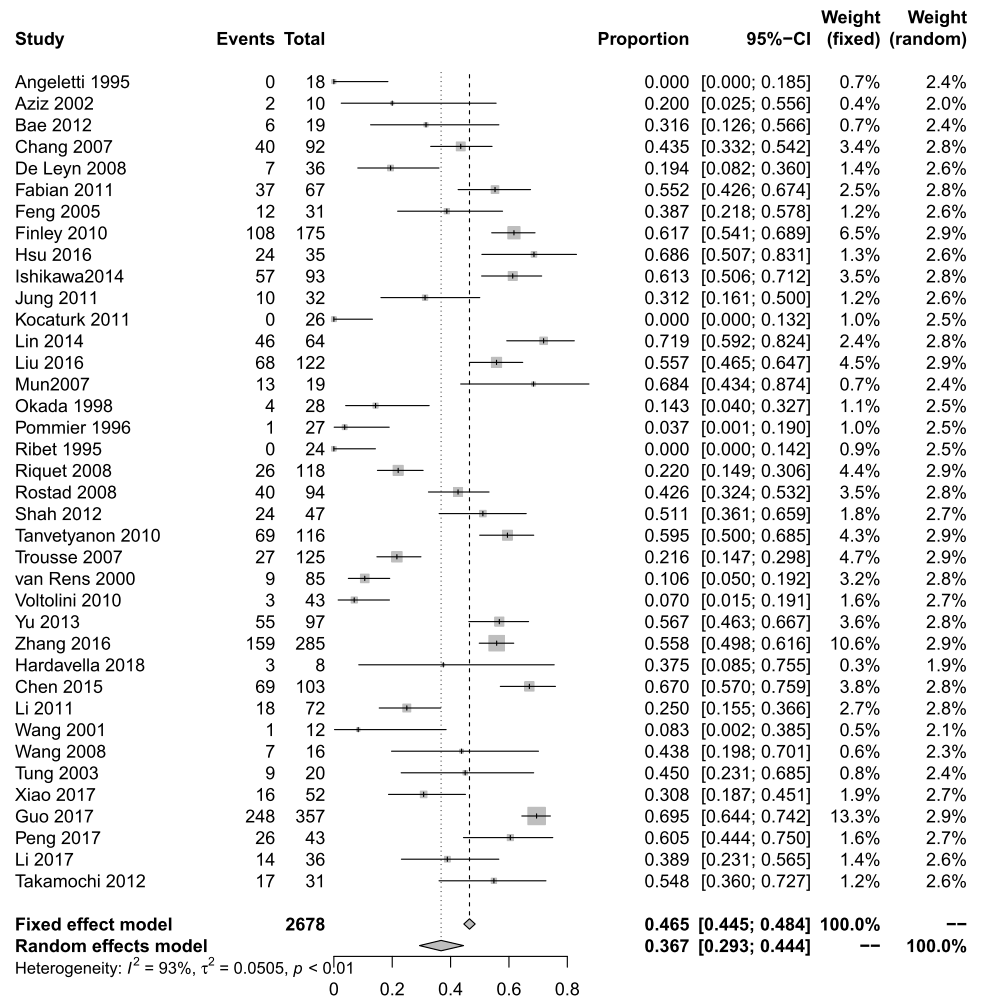
**eFigure 2** Forest chart of meta-analysis for characteristics of synchronous MPLC: gender ratio

Result from random-effects model is used.


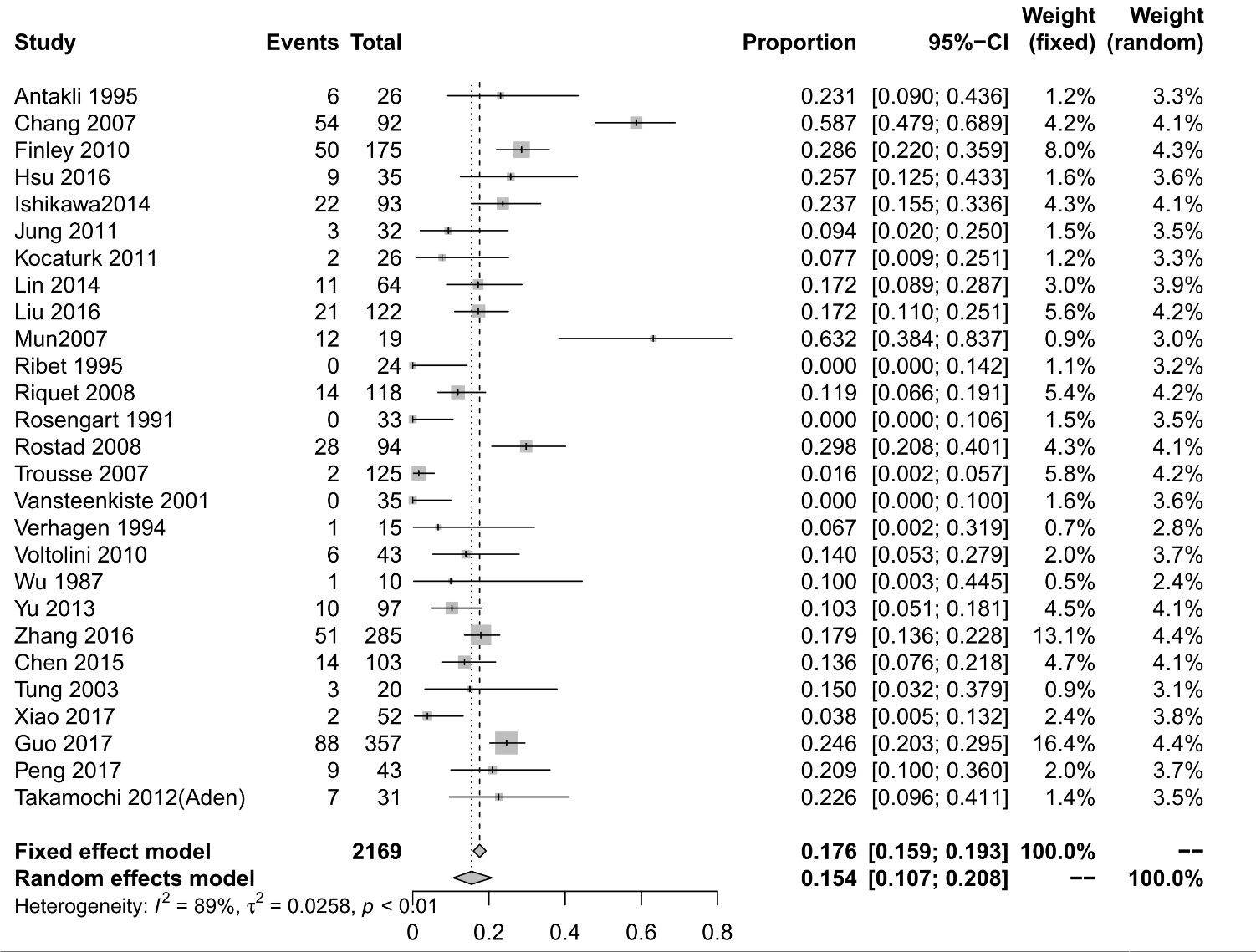
**eFigure 3** Forest chart of meta-analysis for characteristics of synchronous MPLC: the proportion of more than 2 tumors

Result from random-effects model is used.


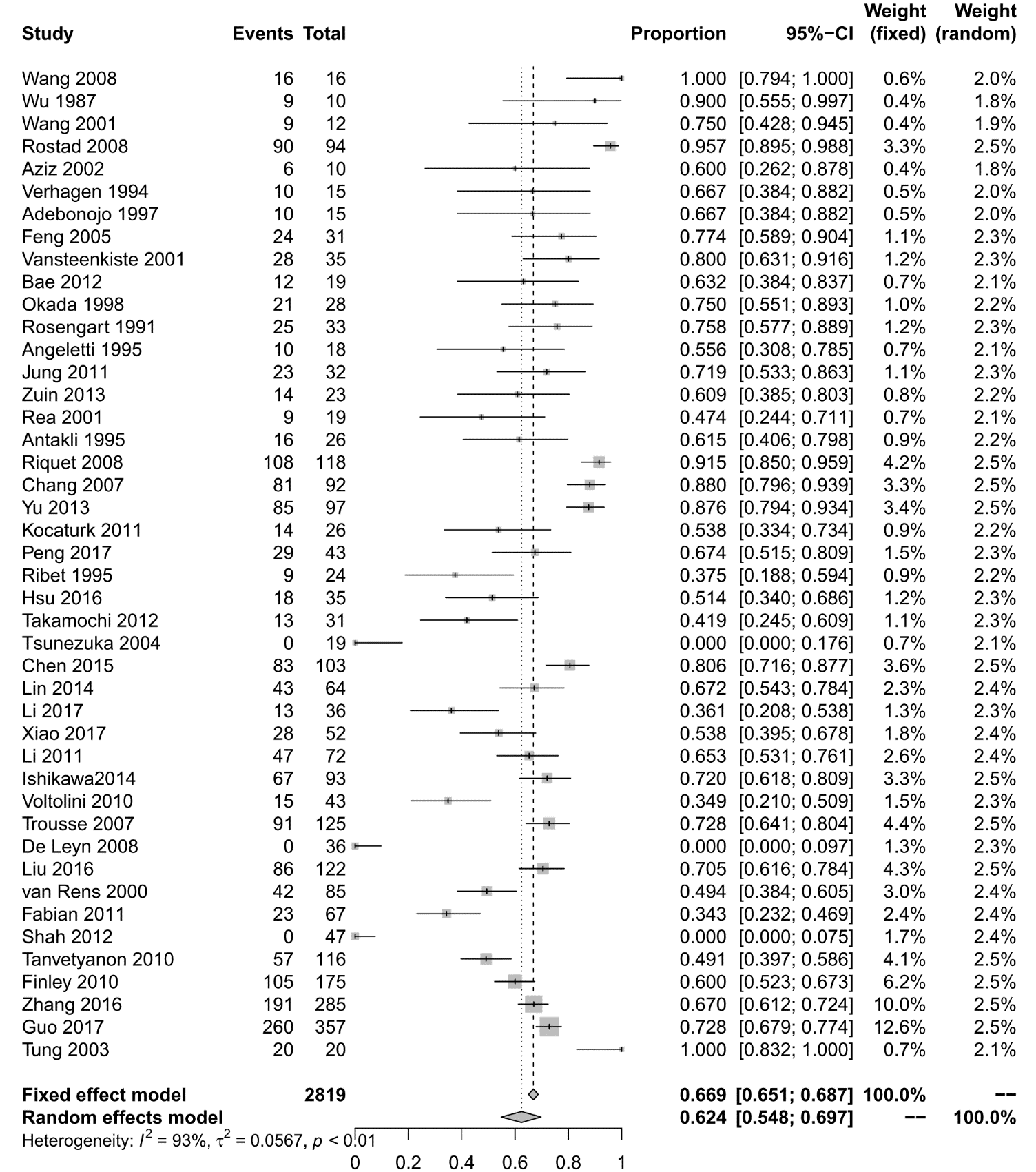
**eFigure 4** Forest chart of meta-analysis for characteristics of synchronous MPLC: proportion of tumors located in unilateral lung

Result from random-effects model is used.


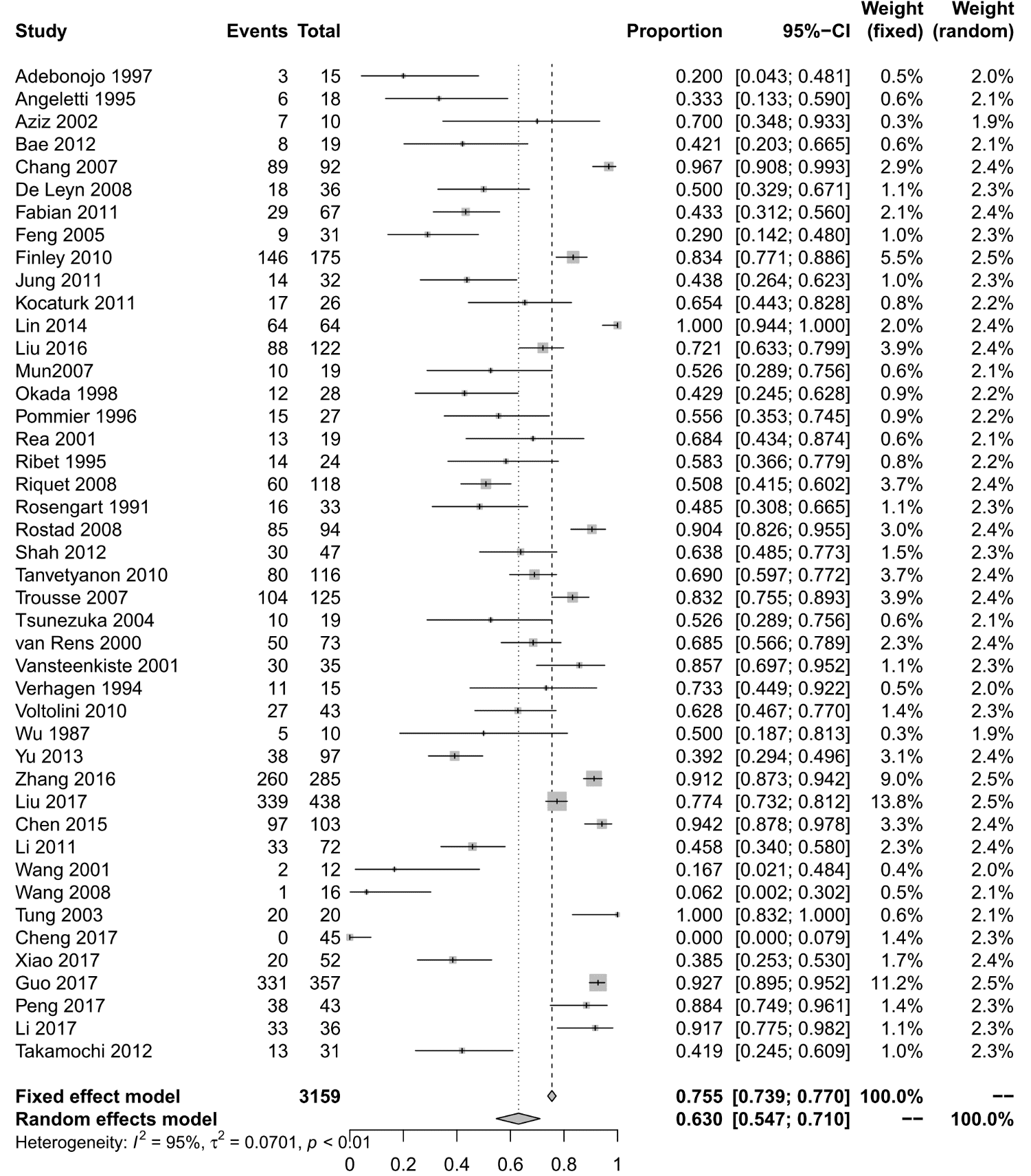
**eFigure 5** Forest chart of meta-analysis for characteristics: the proportion of tumors with identical histology

Result from random-effects model is used.

**Appendix 9 Forest chart for the proportion of sMPLC in lung cancer**


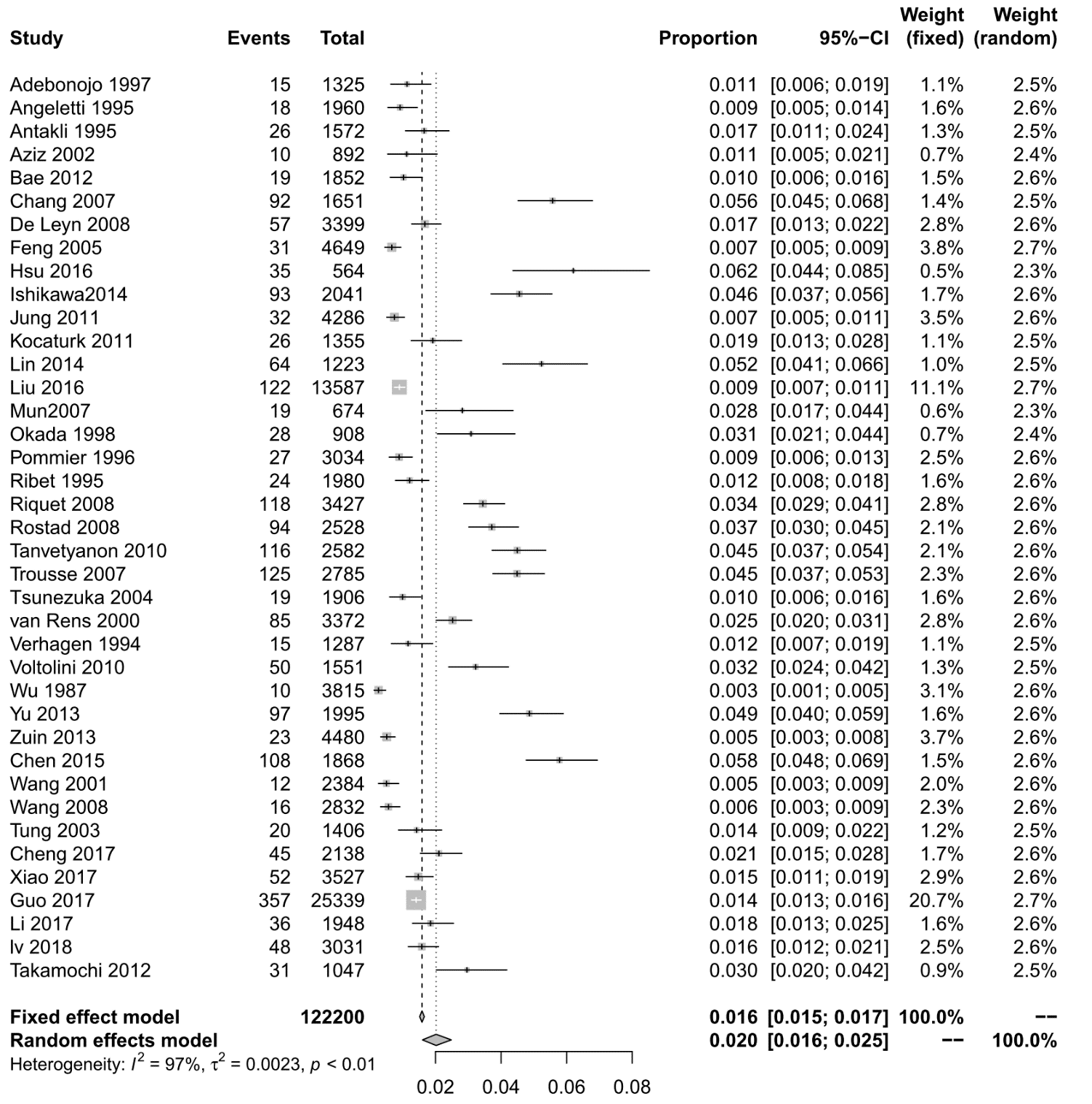
**eFigure 6** Forest chart of meta-analysis for the proportion of sMPLC in lung cancer

Result from random-effects model is used.


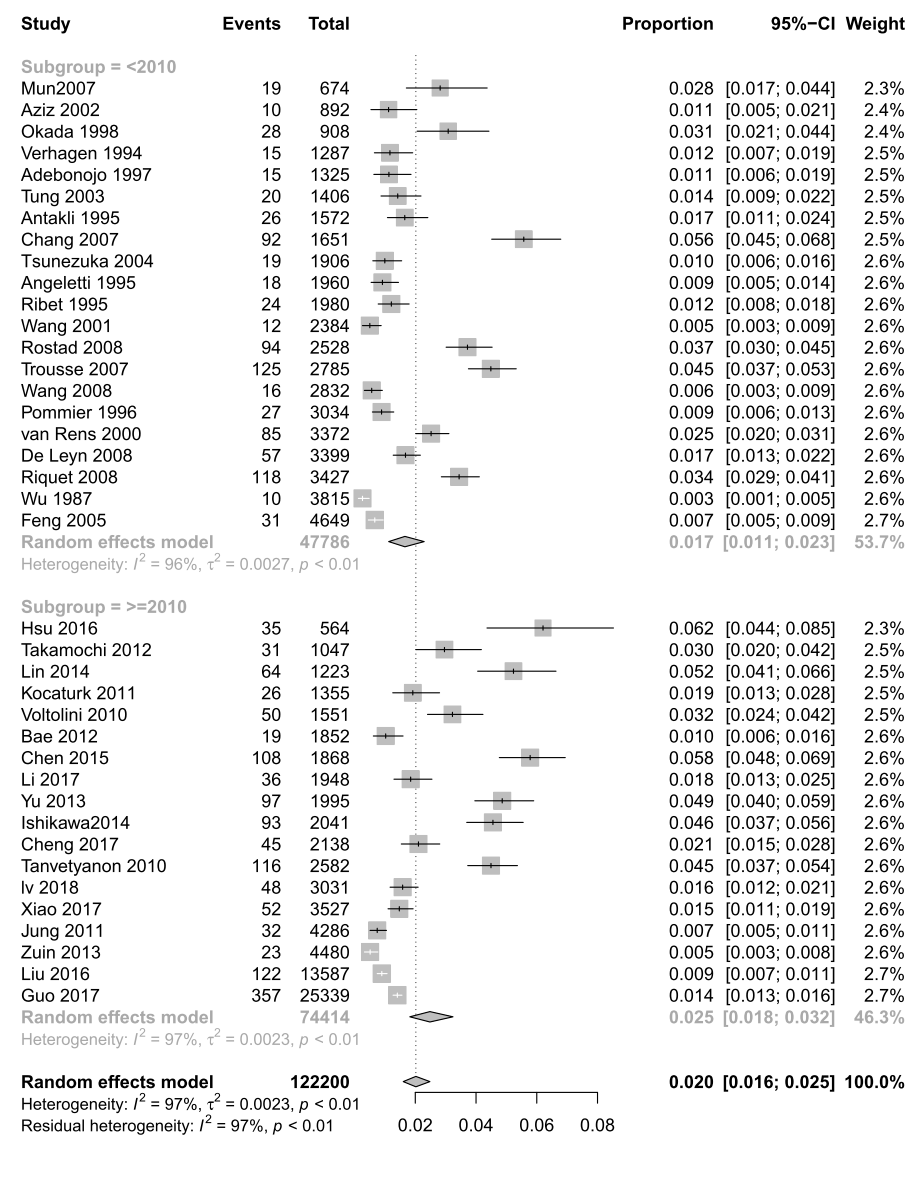
**eFigure 7** Forest chart of subgroup analysis for the proportion of sMPLC according to publication year


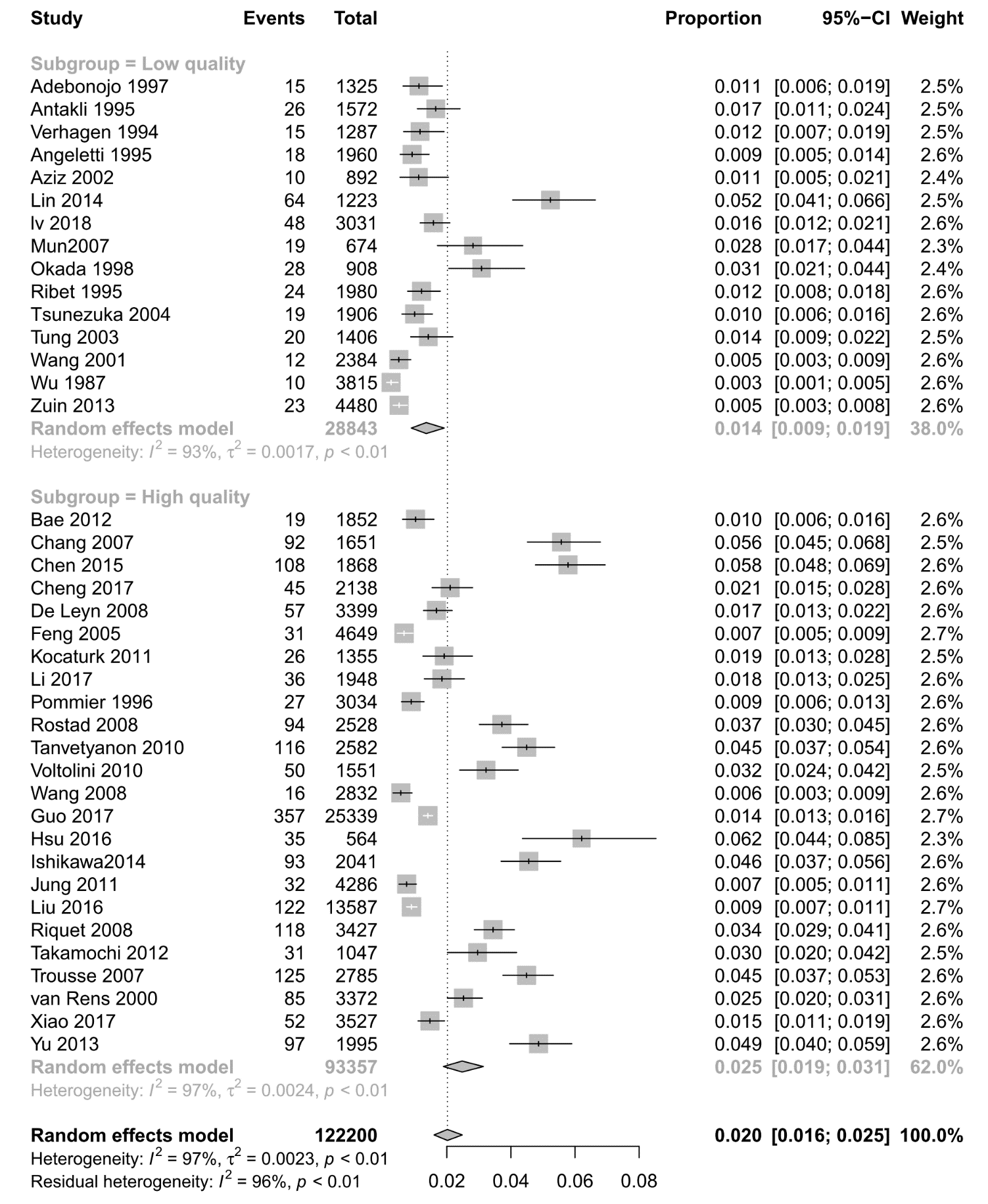
**eFigure 8** Forest chart of subgroup analysis for the proportion of sMPLC according to study quality

**eFigure 9** Sensitivity analysis for the proportion of sMPLC in lung cancer


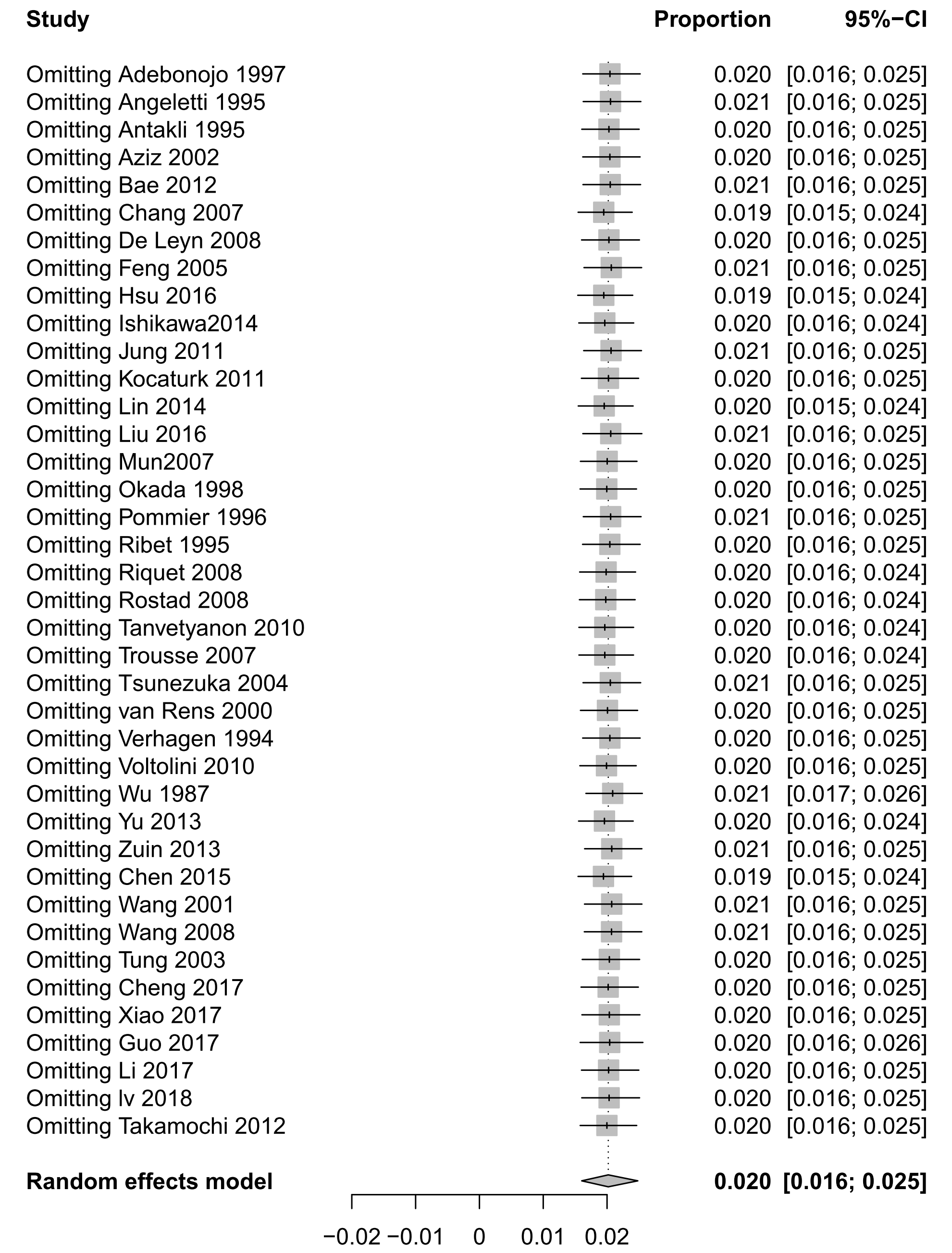


**Appendix 10 Forest chart for postoperative mortality**


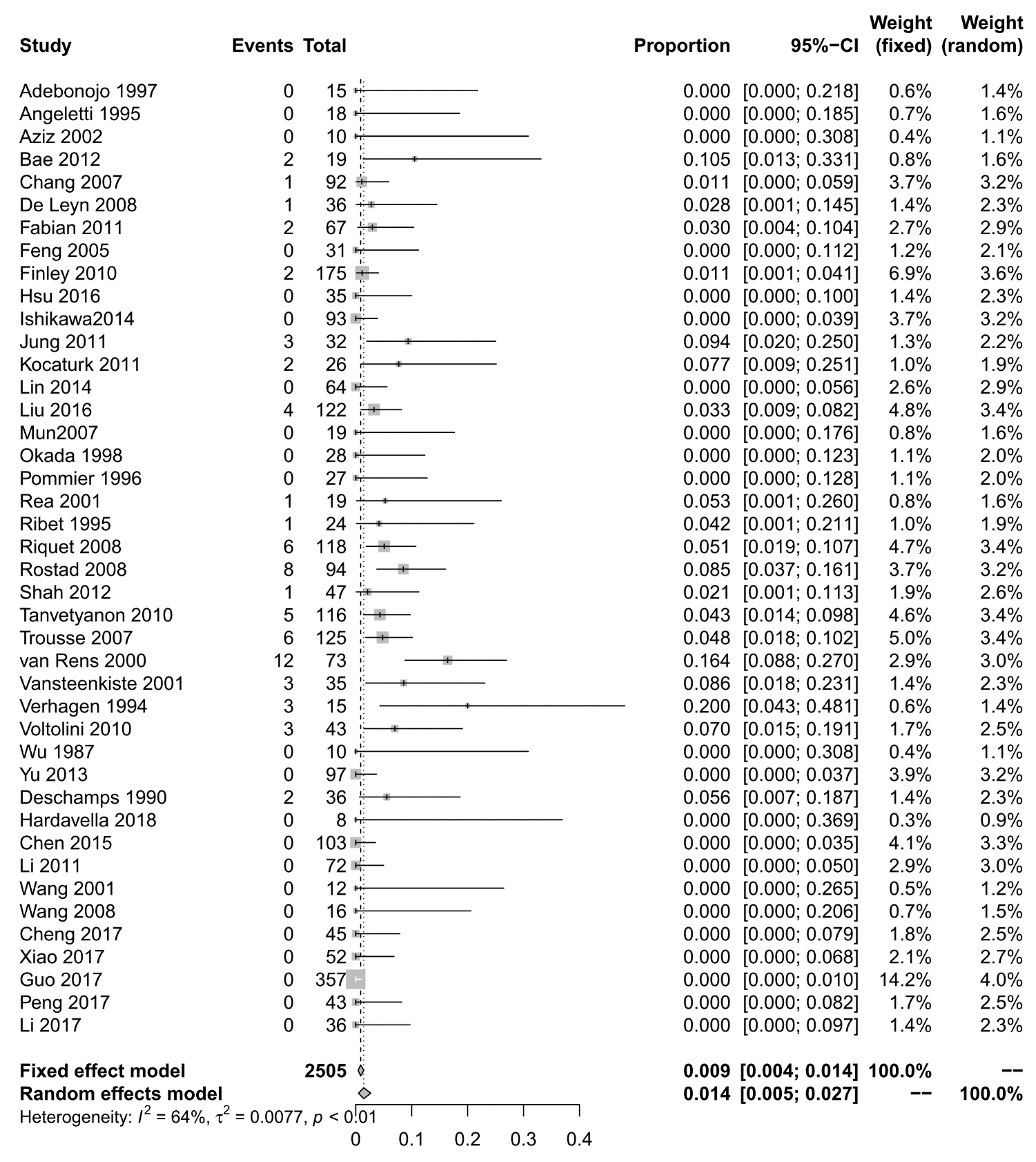
**eFigure 10** Forest chart of meta-analysis for postoperative mortality

Result from random-effects model is used.


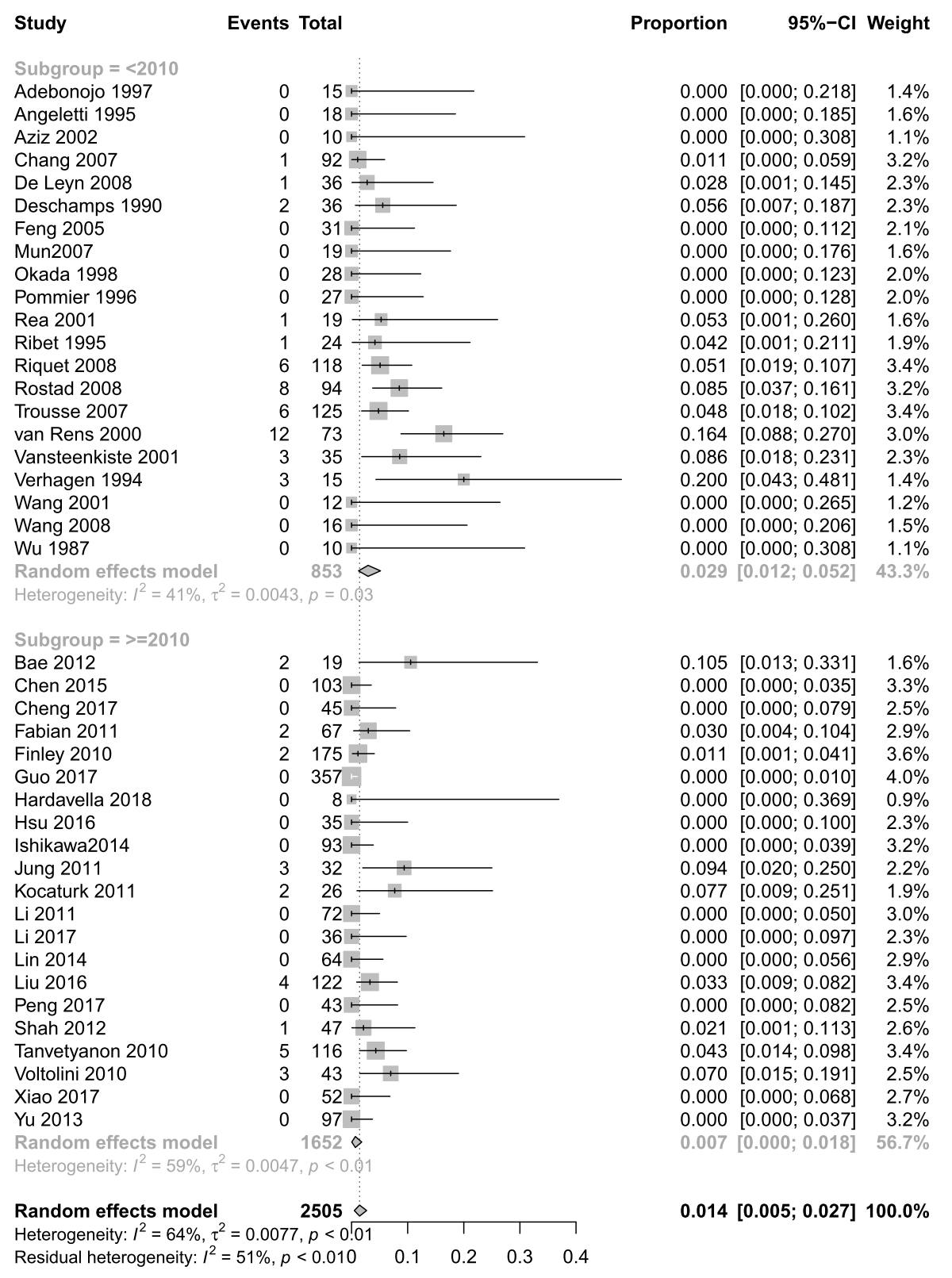
**eFigure 11** Forest chart of subgroup analysis for postoperative mortality according to publication year


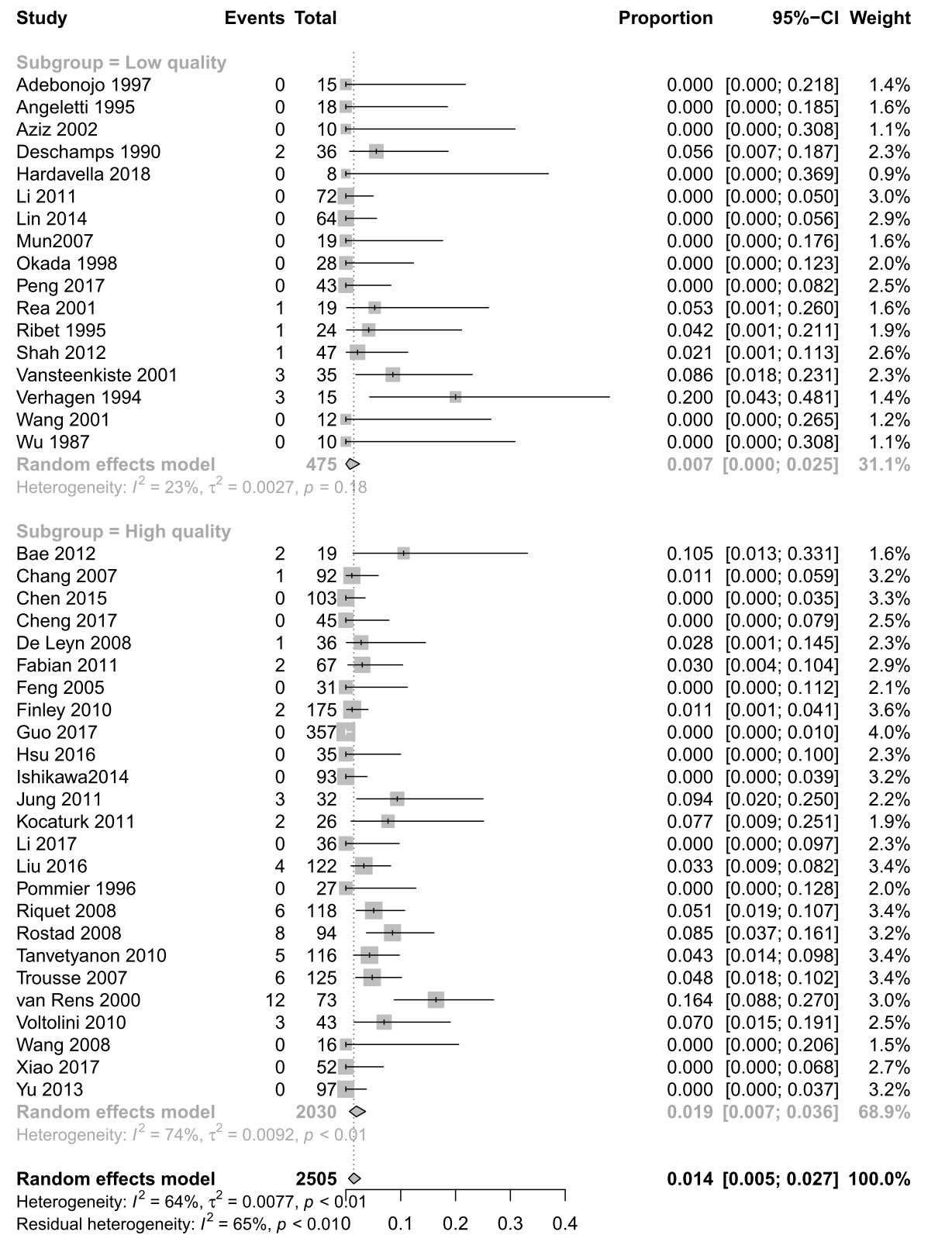
**eFigure 12** Forest chart of subgroup analysis for the postoperative mortality according to study quality

**eFigure 13** Sensitivity analysis for the postoperative mortality


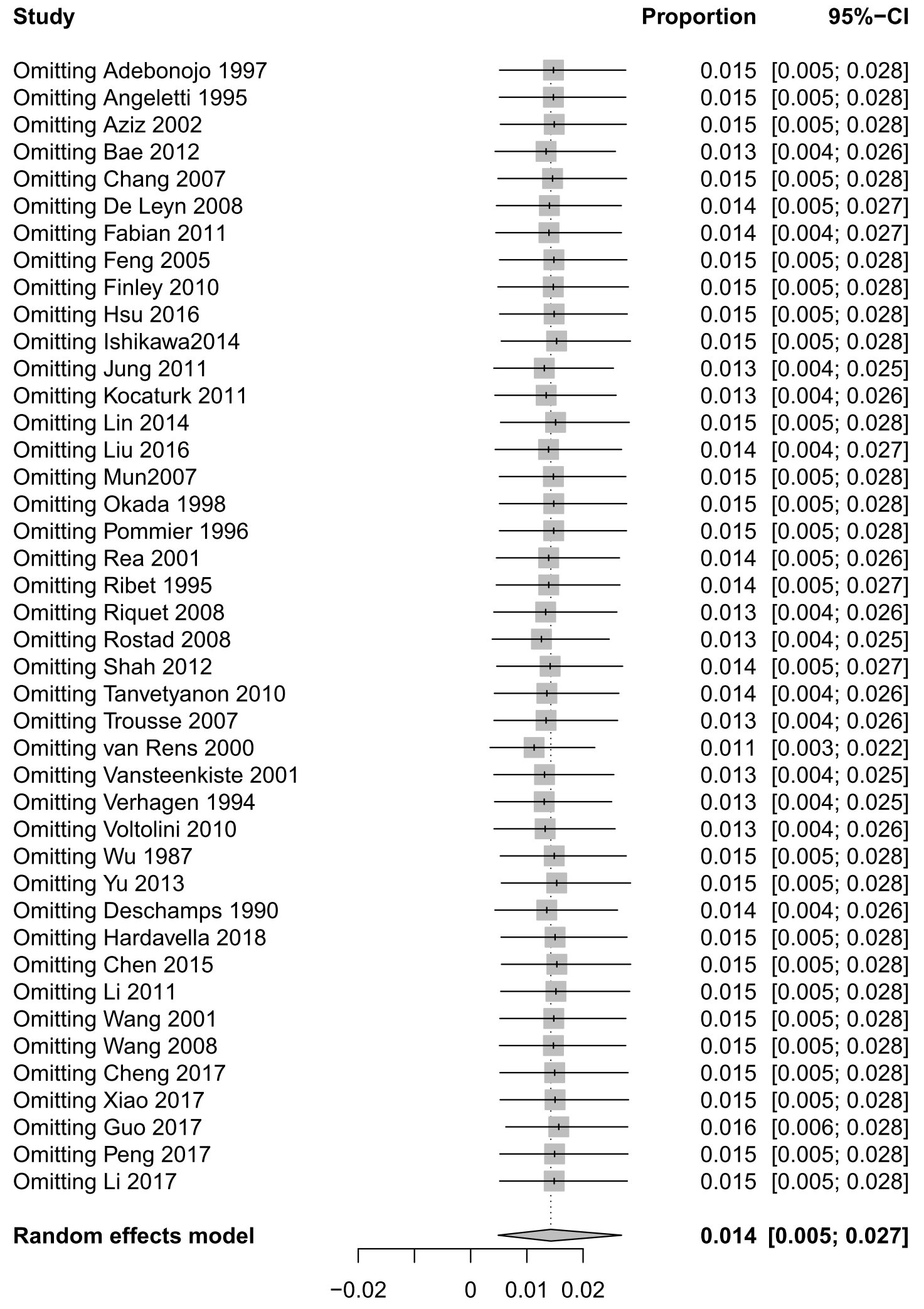


**Appendix 11 Forest chart for the survival rate of sMPLC**


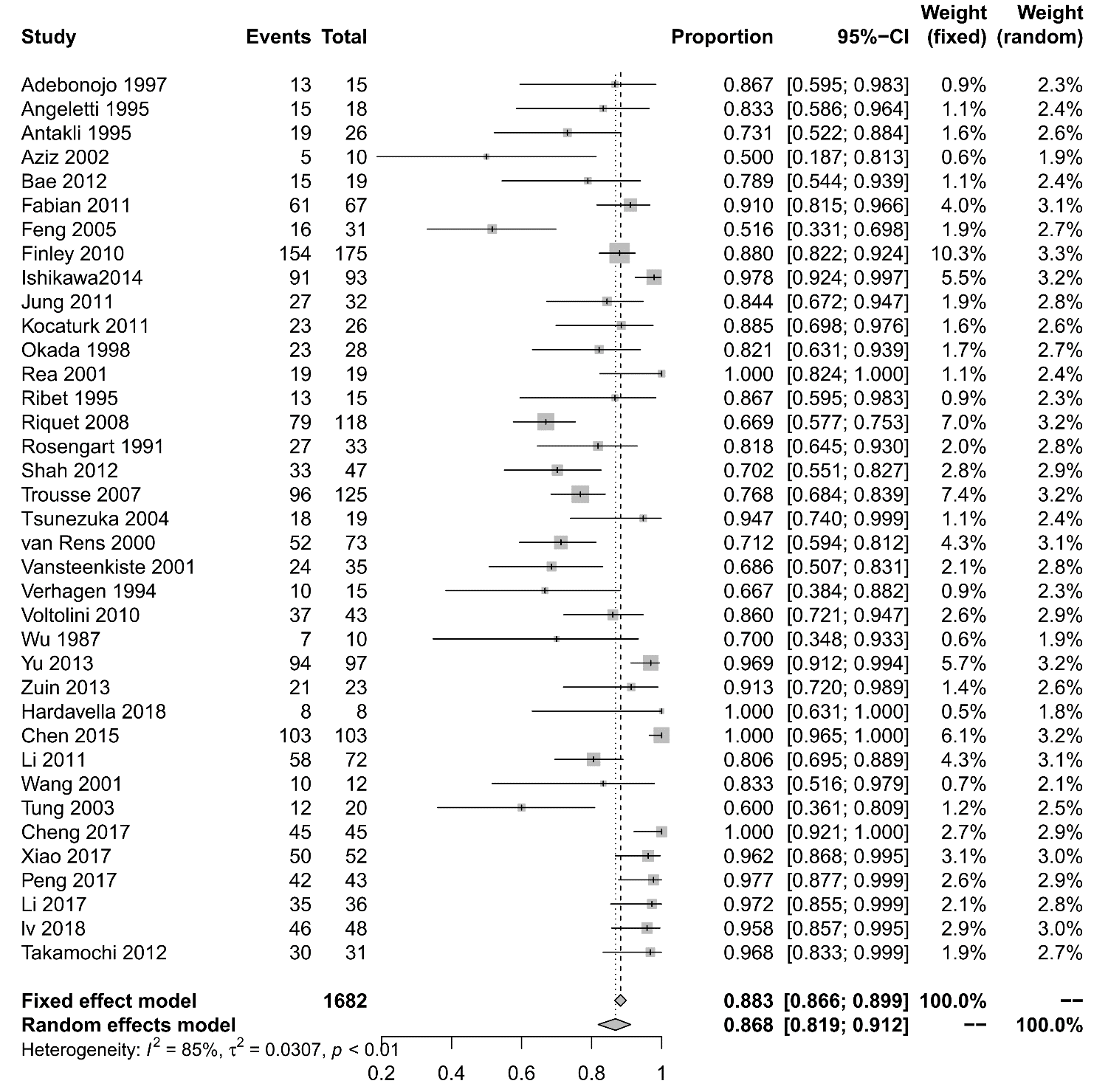
**eFigure 14** Forest chart of meta-analysis for 1-year survival rate

Result from random-effects model is used.


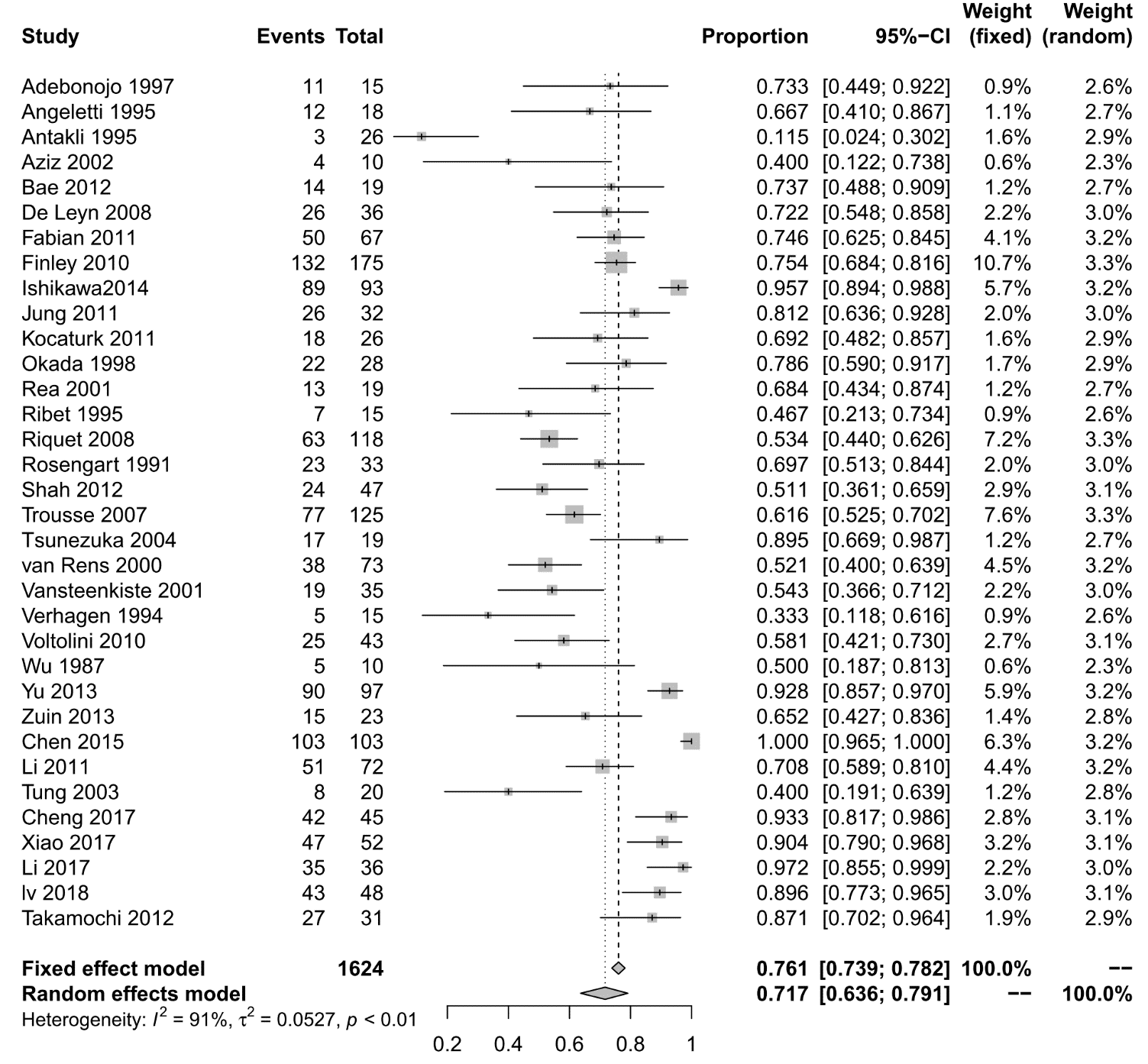
**eFigure 15** Forest chart of meta-analysis for 2-year survival rate

Result from random-effects model is used.


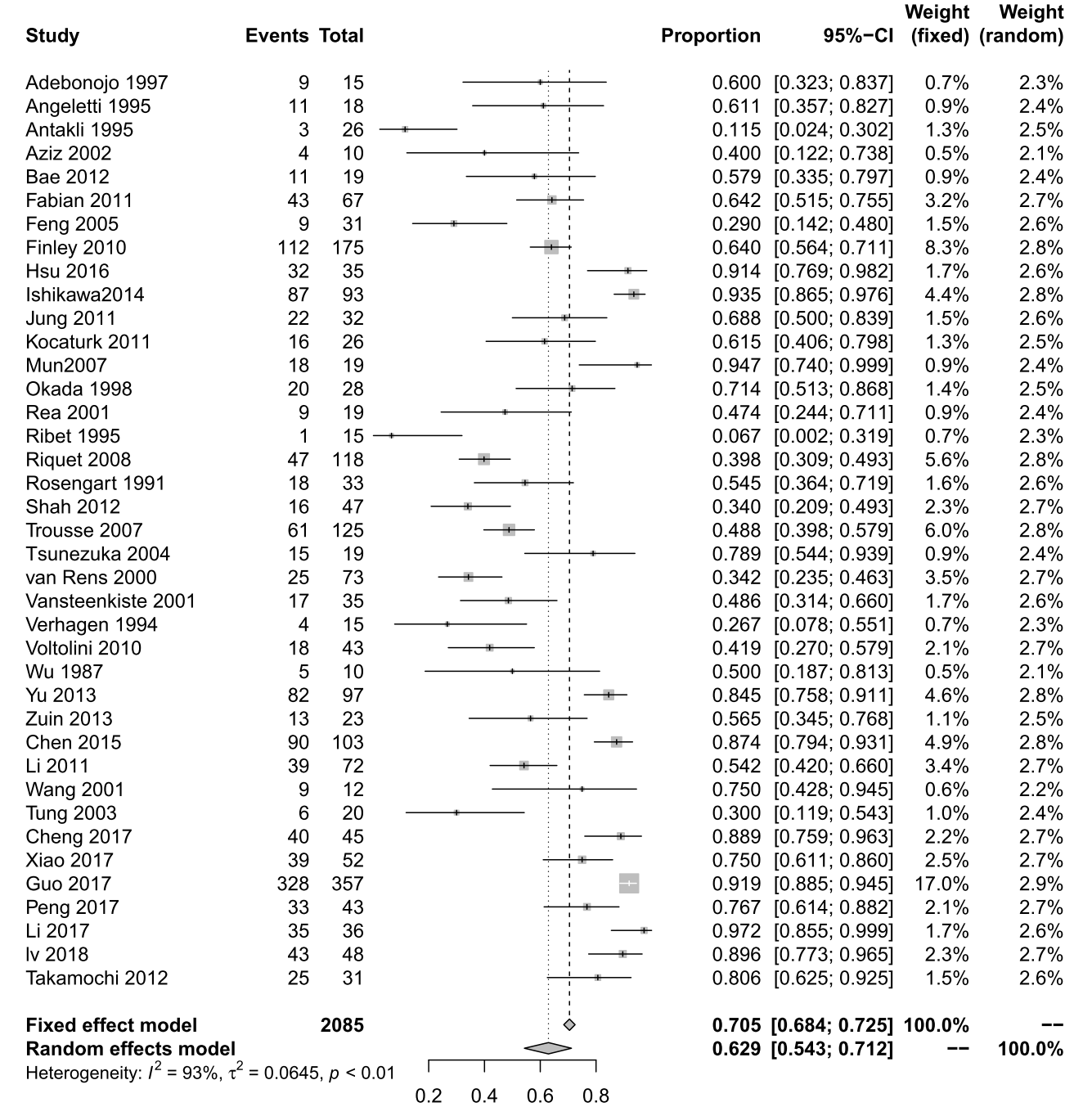
**eFigure 16** Forest chart of meta-analysis for 3-year survival rate

Result from random-effects model is used.


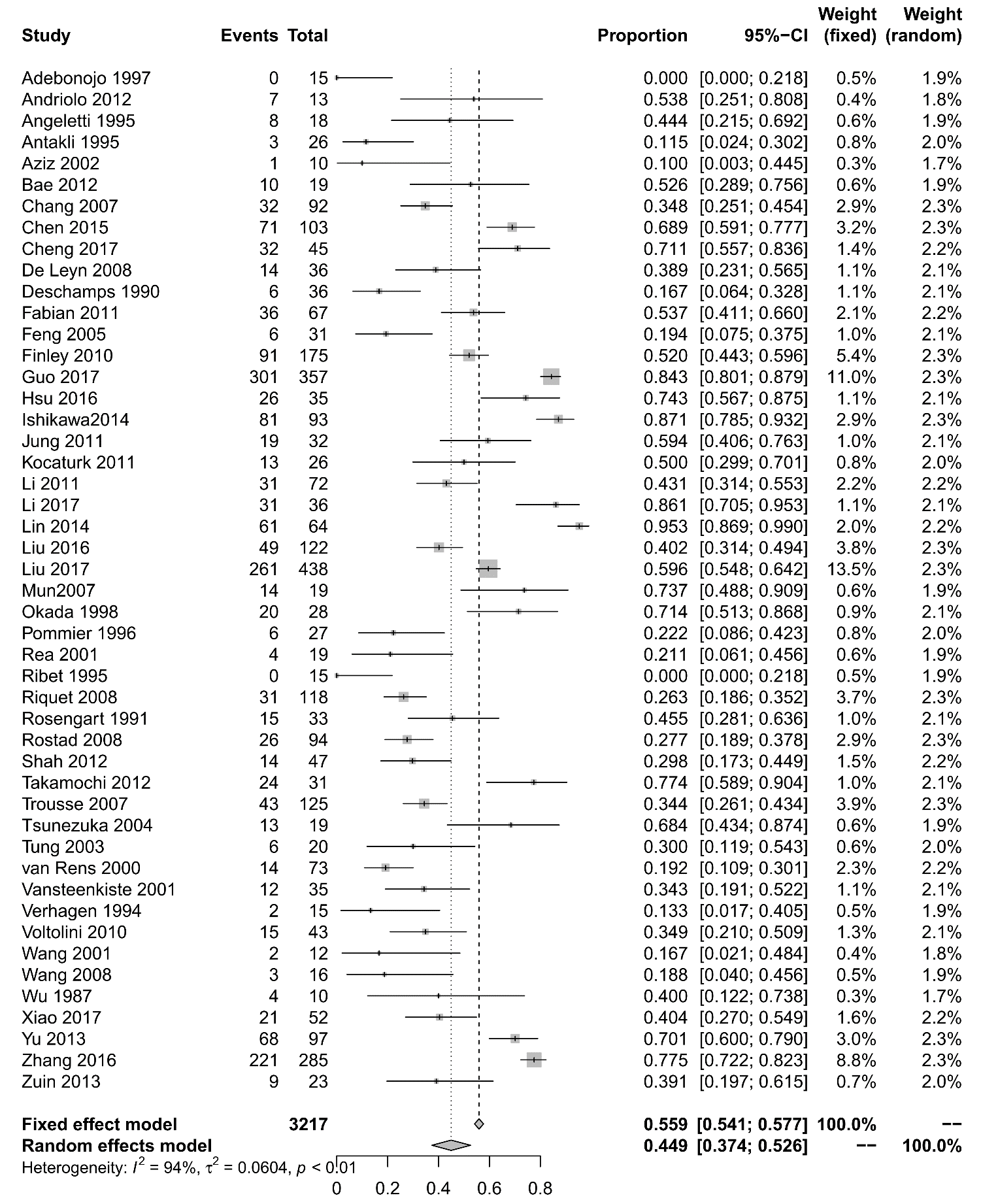
**eFigure 17** Forest chart of meta-analysis for 5-year survival rate

Result from random-effects model is used.

**eFigure 18** Forest chart of subgroup analysis for 5-year survival rate according to publication year


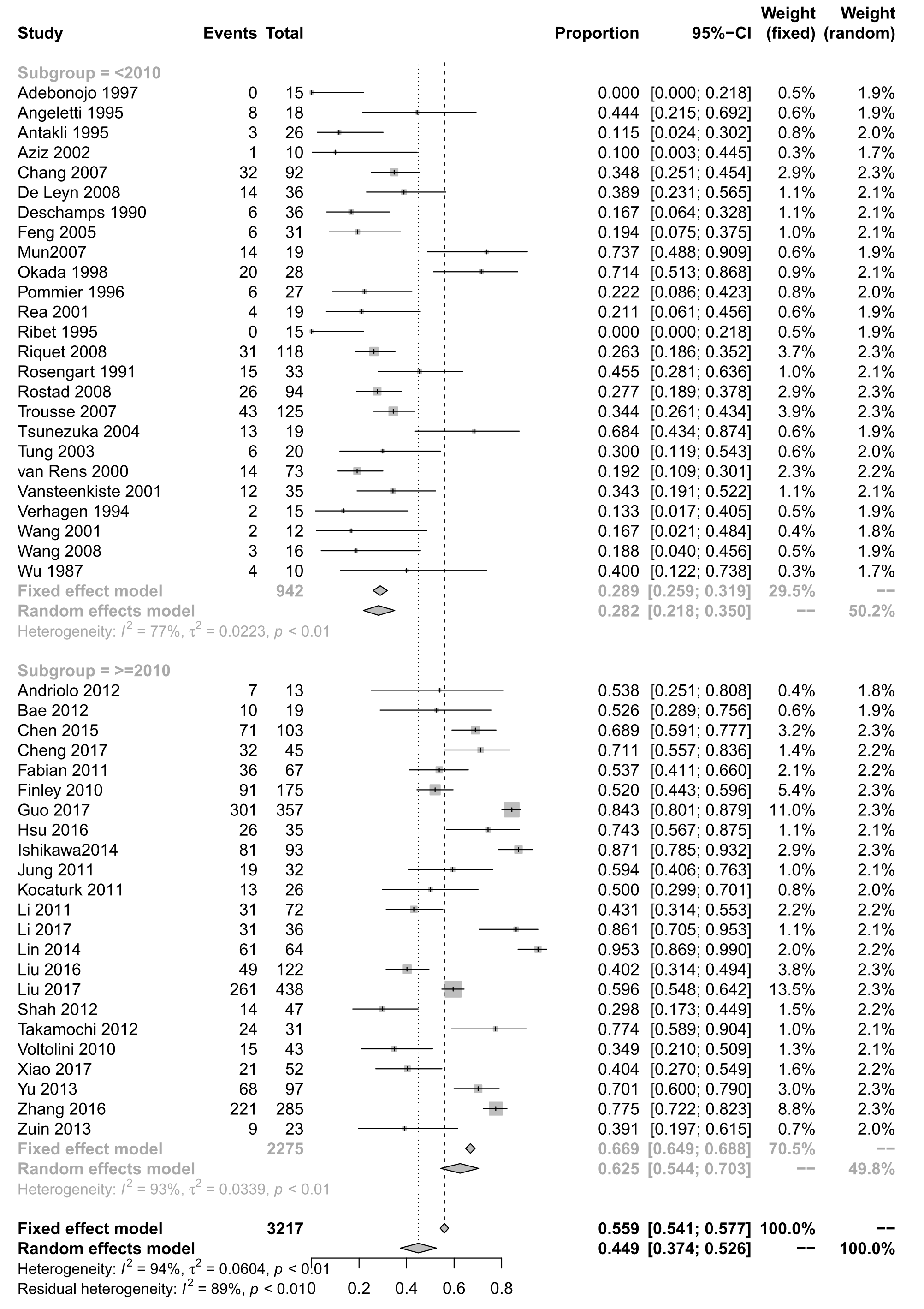
 Result from random-effects model is used.


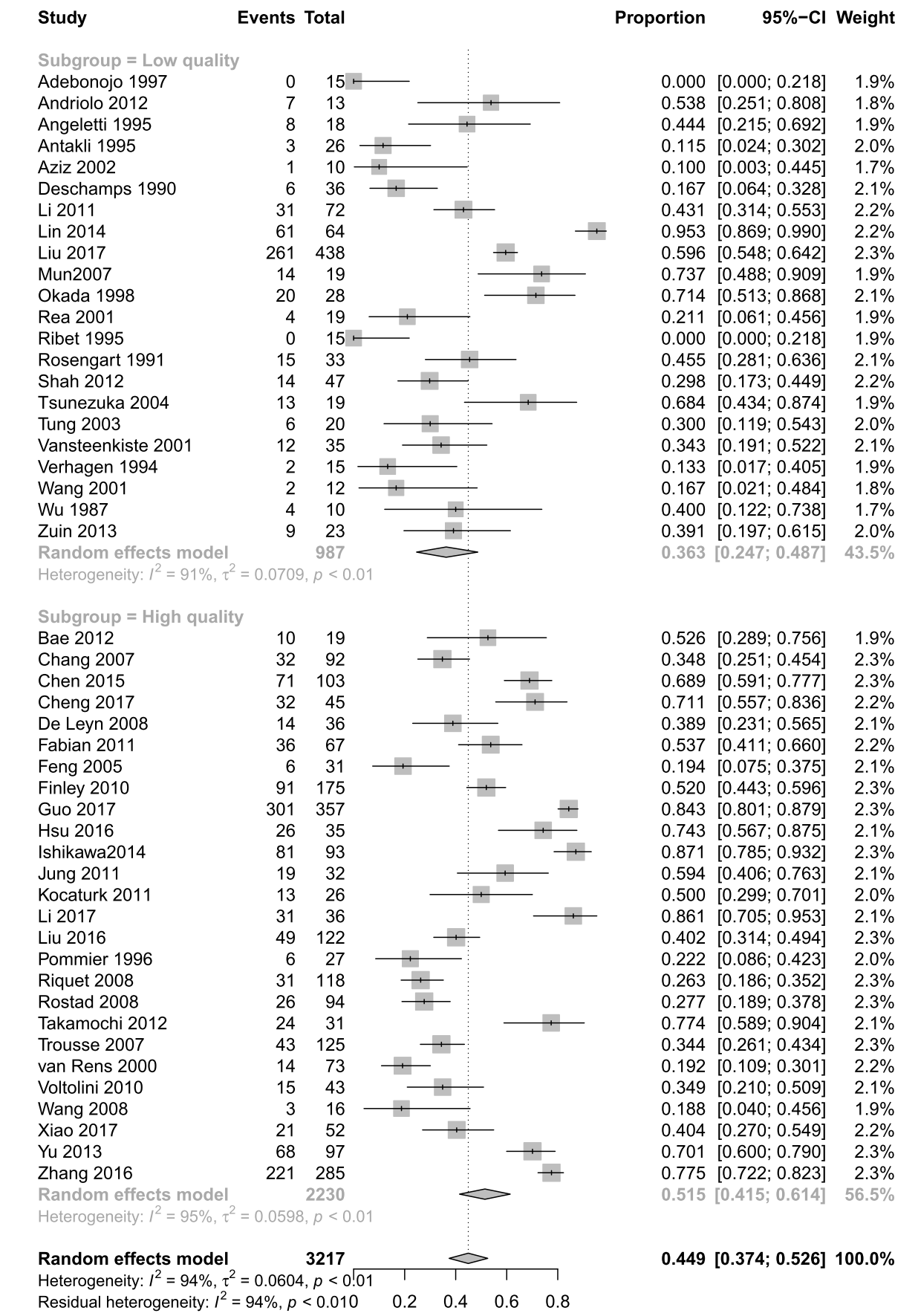
**eFigure 19** Forest chart of subgroup analysis for 5-year survival rate according to study quality

**eFigure 20** Sensitivity analysis for 5-year survival rate


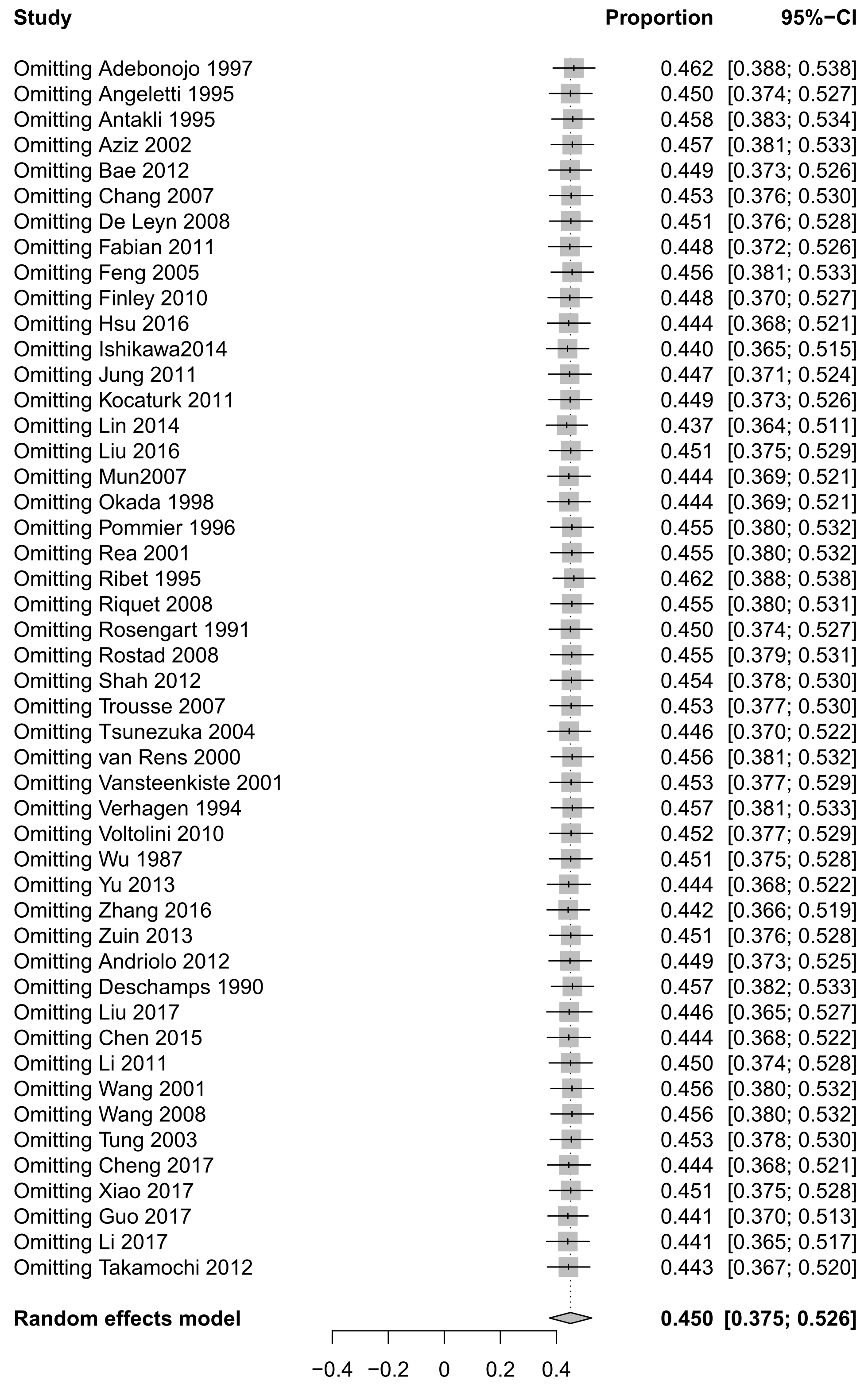


**Appendix 12 Effect of various clinical parameters on 5-year survival rate**


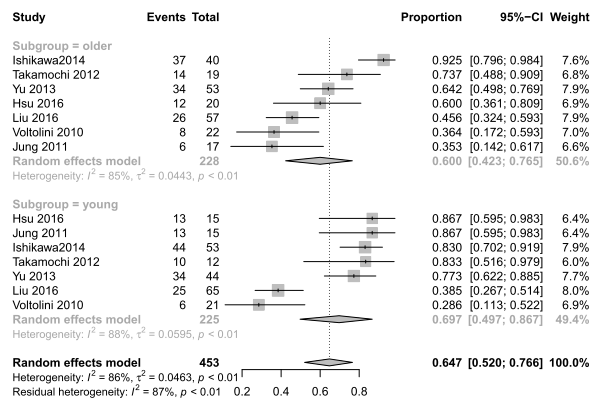
**eFigure 21** Forest chart of additional analysis for 5-year survival rate according to clinical parameters: age


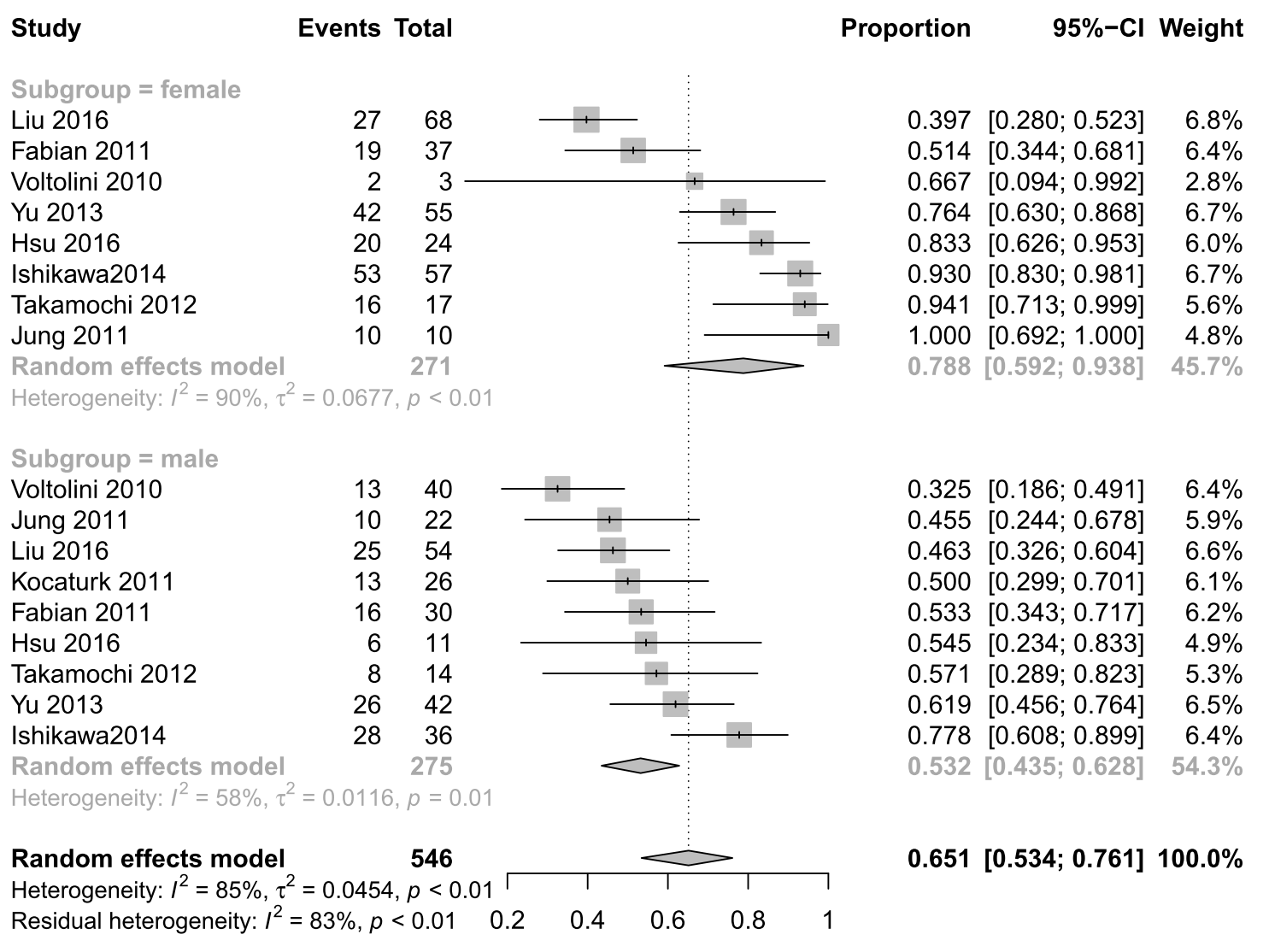
**eFigure 22** Forest chart of additional analysis for 5-year survival rate according to clinical parameters: gender


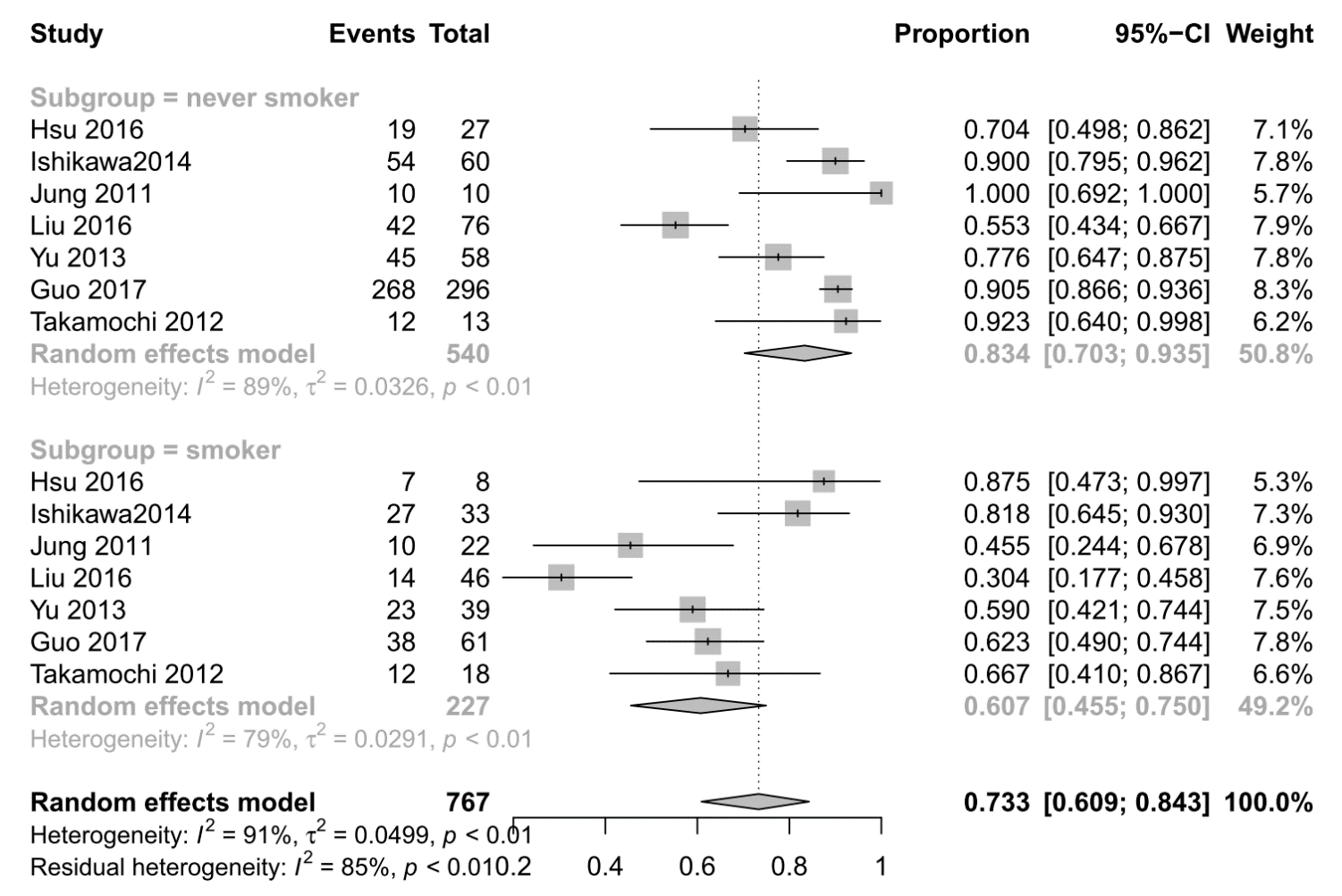
**eFigure 23** Forest chart of additional analysis for 5-year survival rate according to clinical parameters: smoking status

**eFigure 24** Forest chart of additional analysis for 5-year survival rate according to clinical
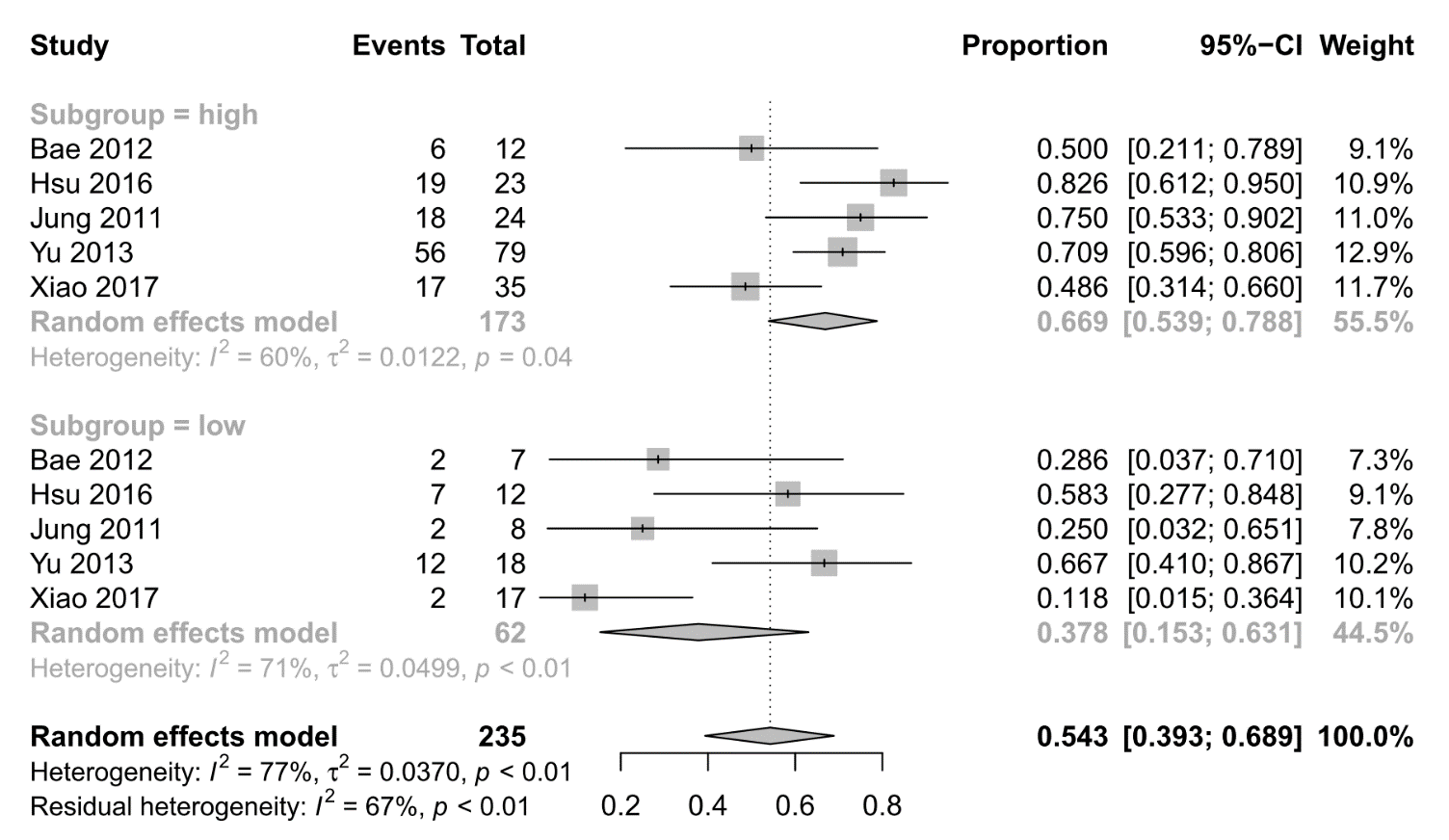
parameters: FEV1


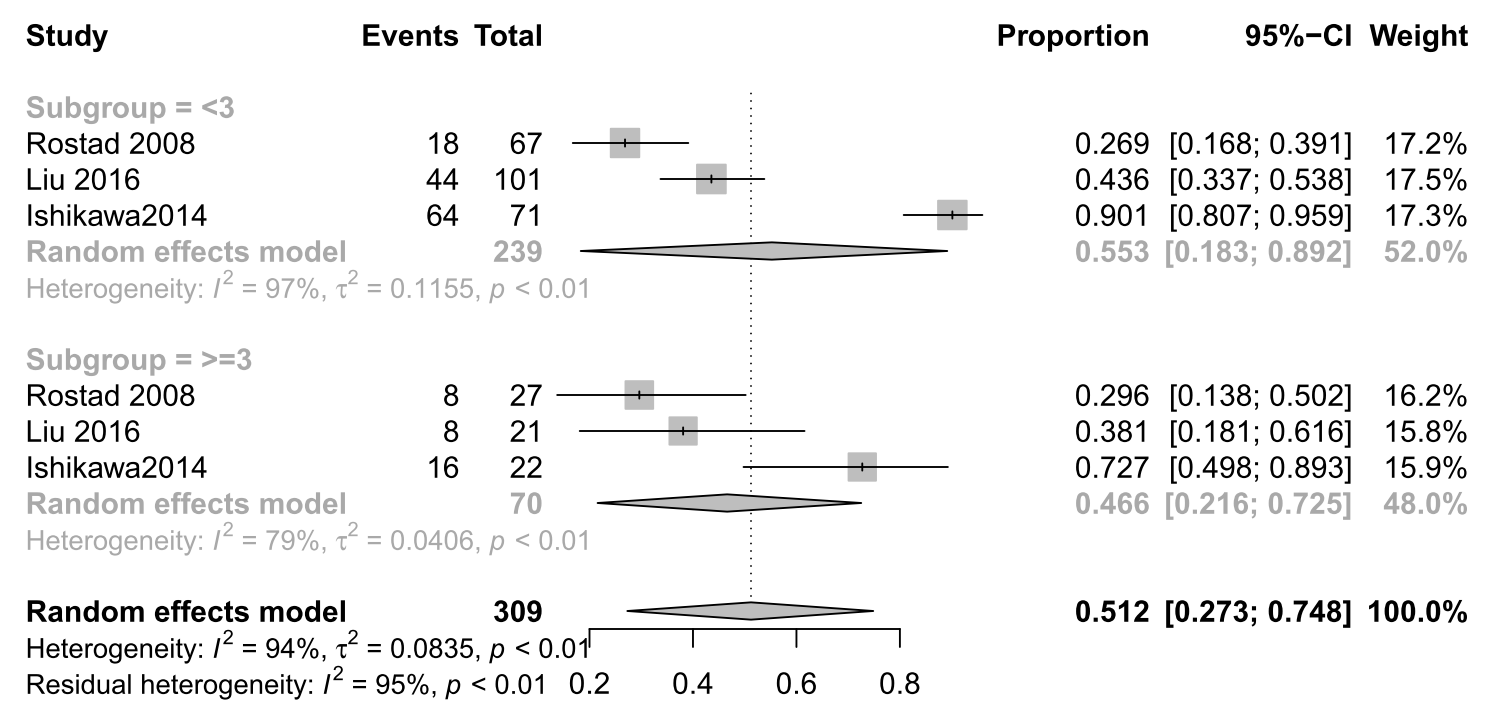
**eFigure 25** Forest chart of additional analysis for 5-year survival rate according to clinical parameters: number of tumors


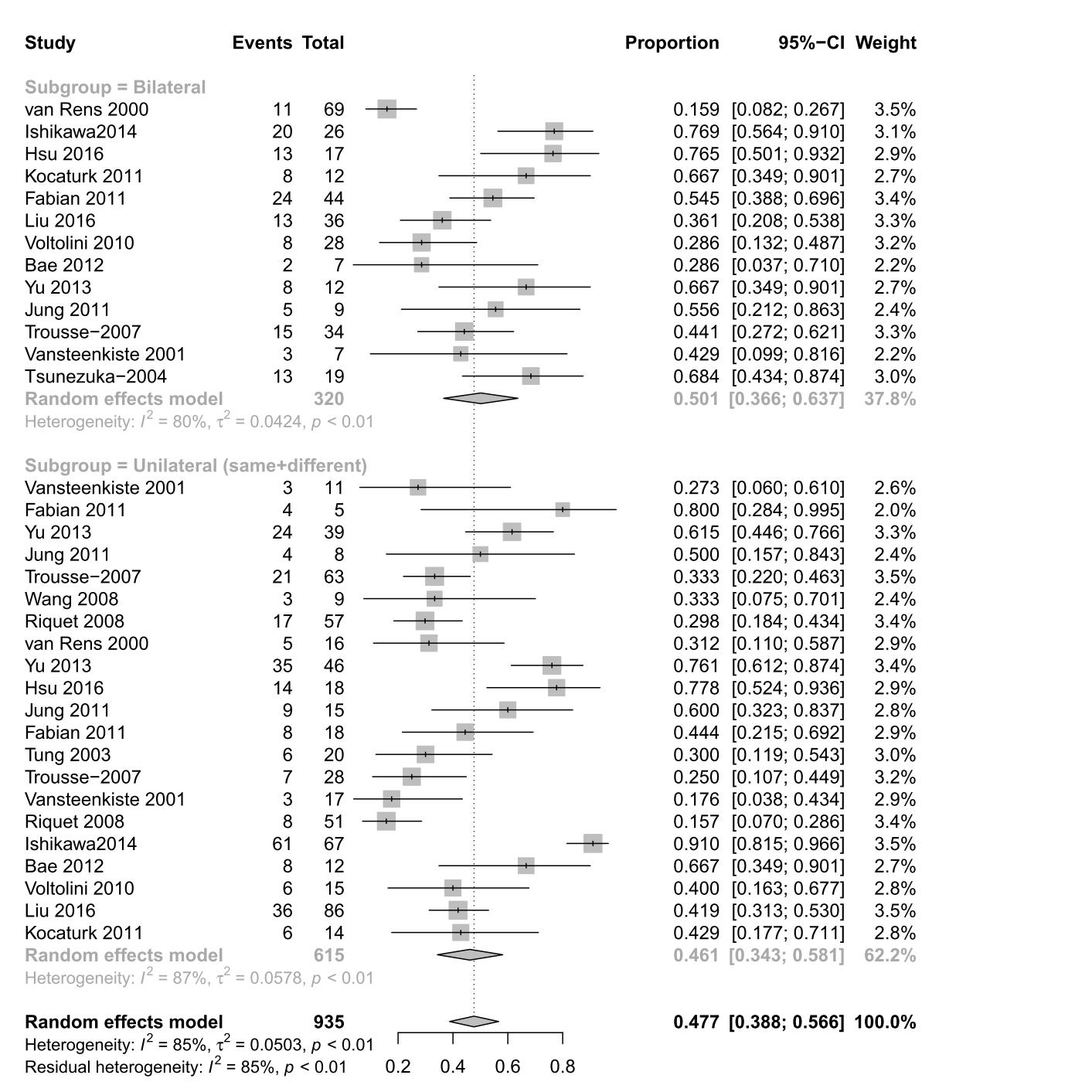
**eFigure 26** Forest chart of additional analysis for 5-year survival rate according to clinical parameters: tumor location


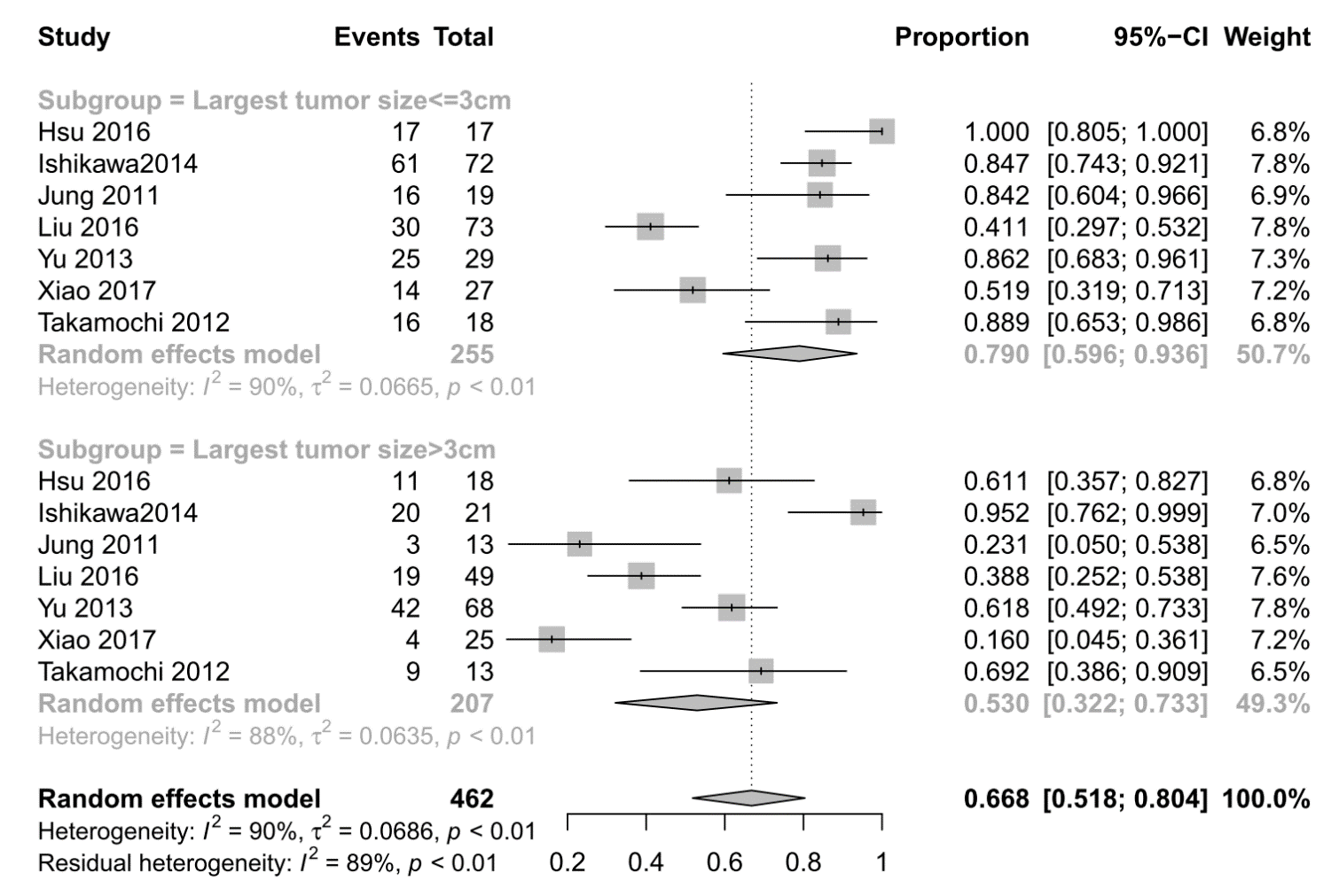
**eFigure 27** Forest chart of additional analysis for 5-year survival rate according to clinical parameters: tumor size


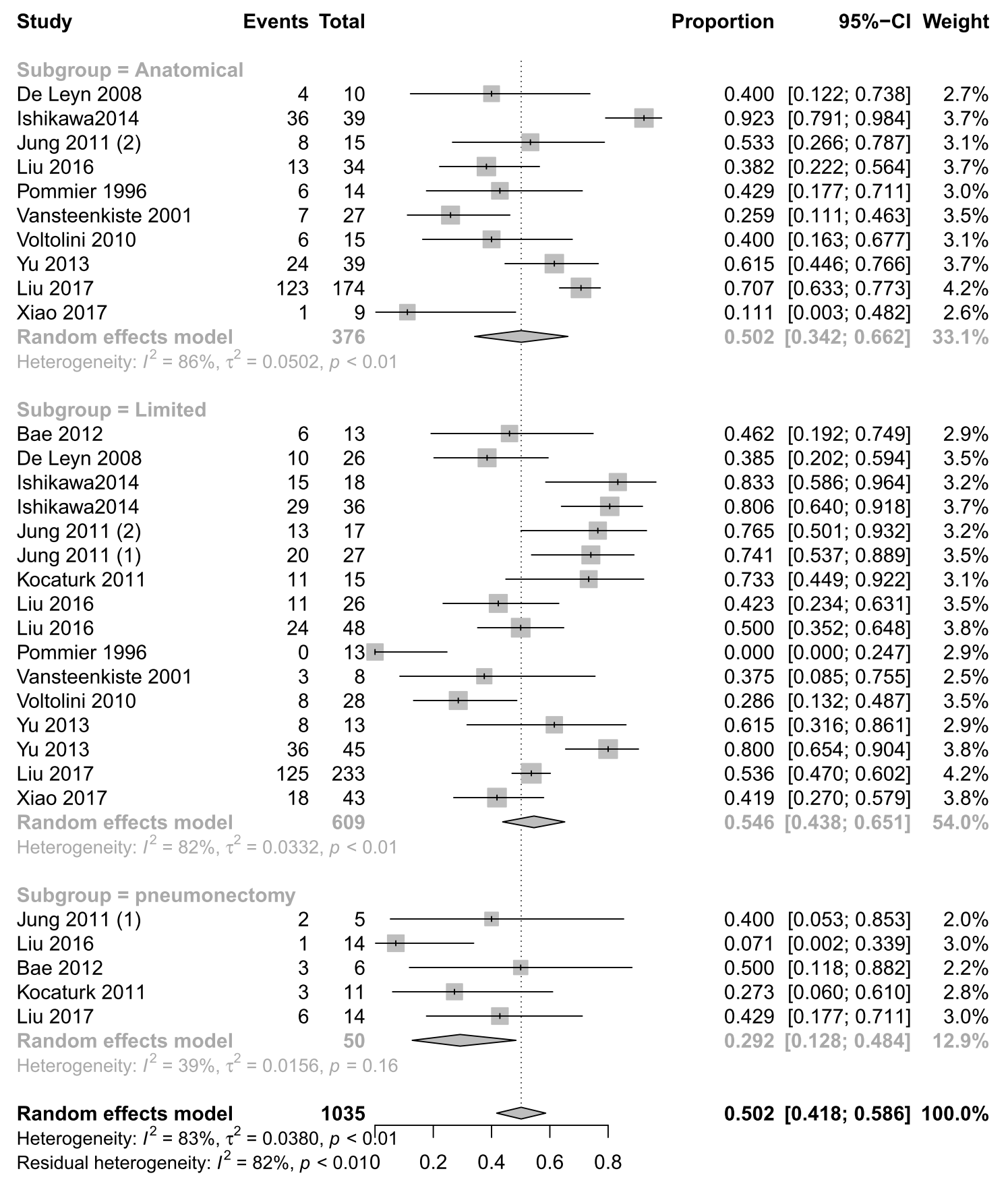
**eFigure 28** Forest chart of additional analysis for 5-year survival rate according to clinical parameters: surgery methods


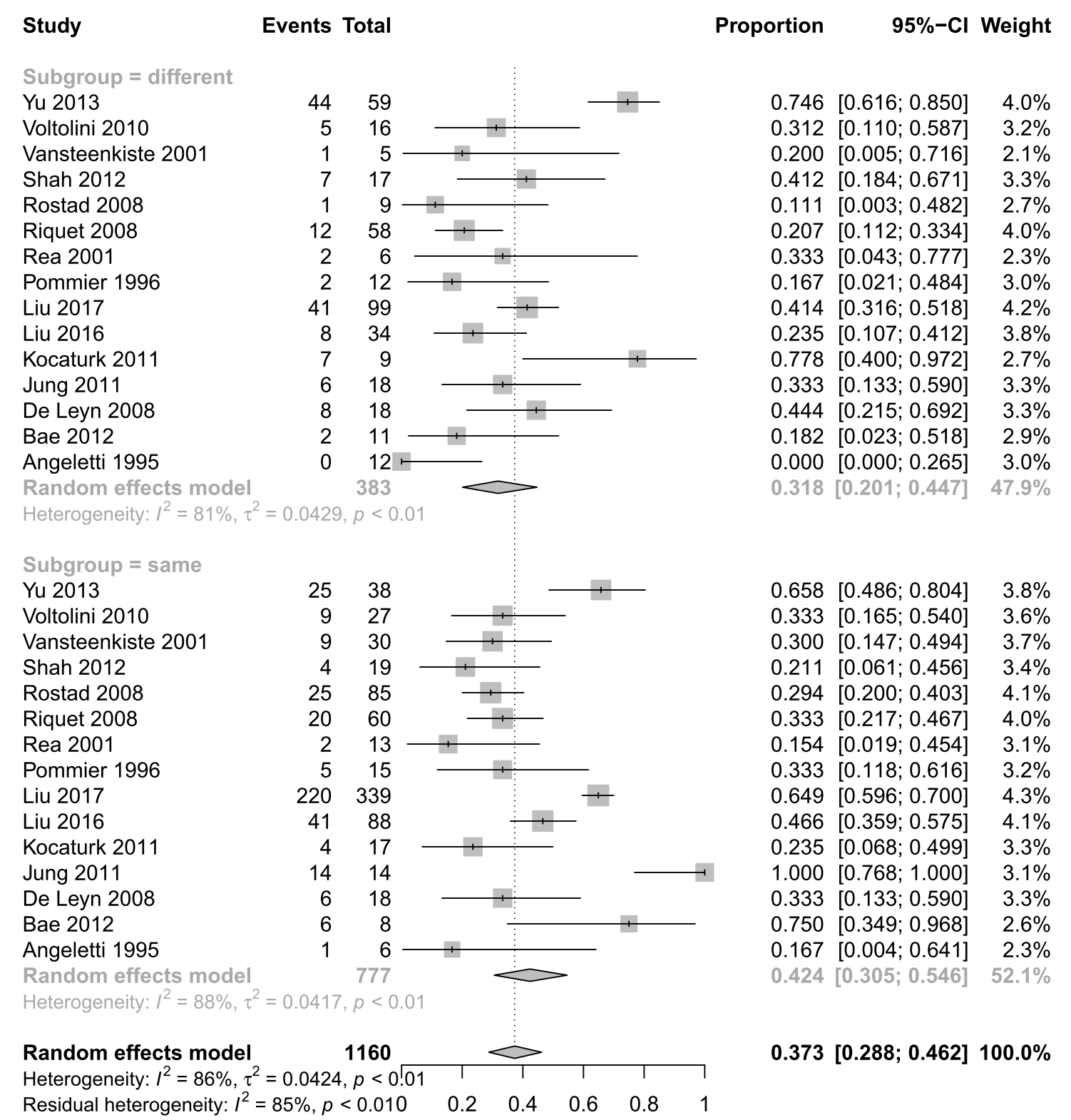
**eFigure 29** Forest chart of additional analysis for 5-year survival rate according to clinical parameters: tumor histology


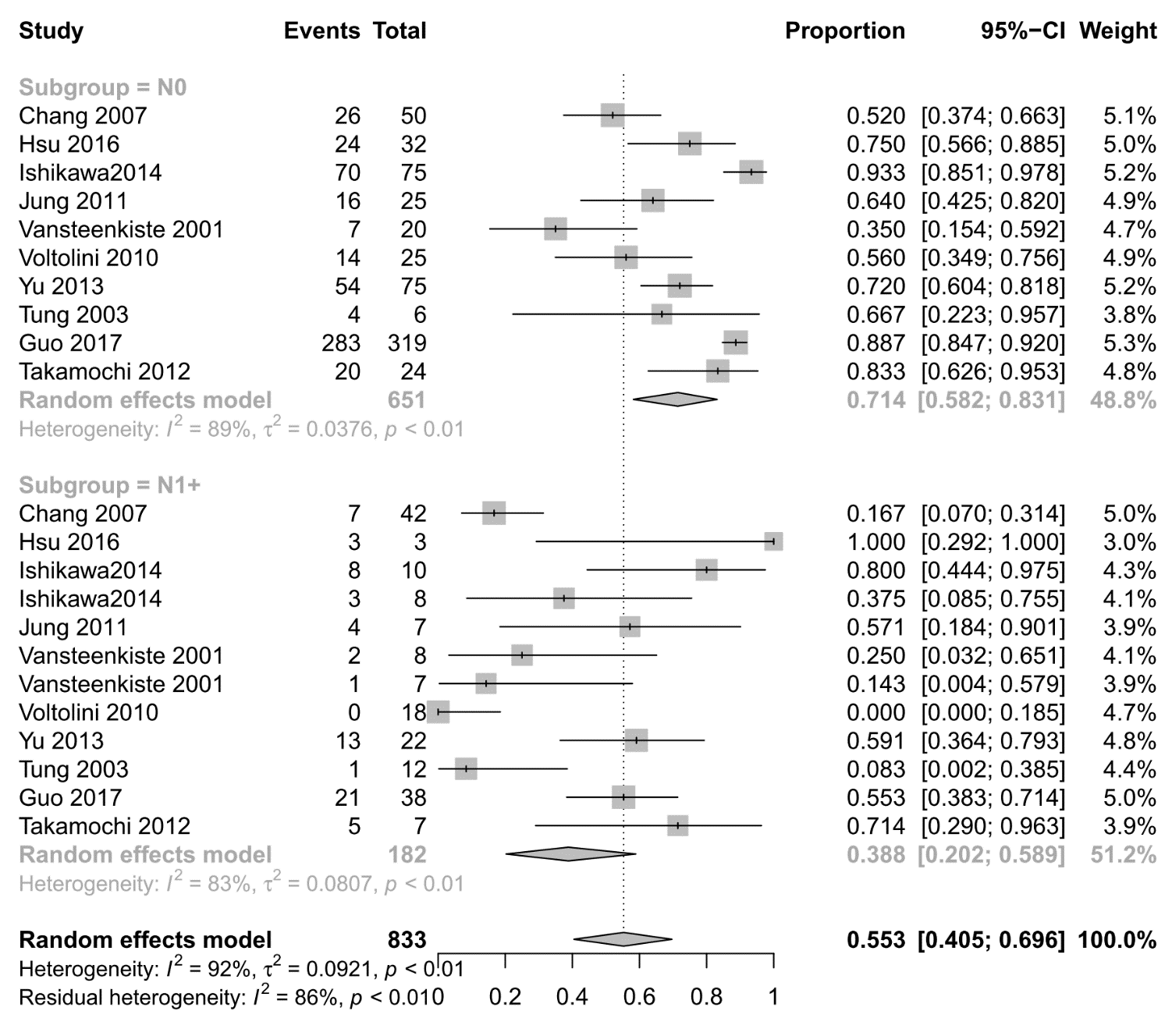
**eFigure 30** Forest chart of additional analysis for 5-year survival rate according to clinical parameters: lymph node metastasis


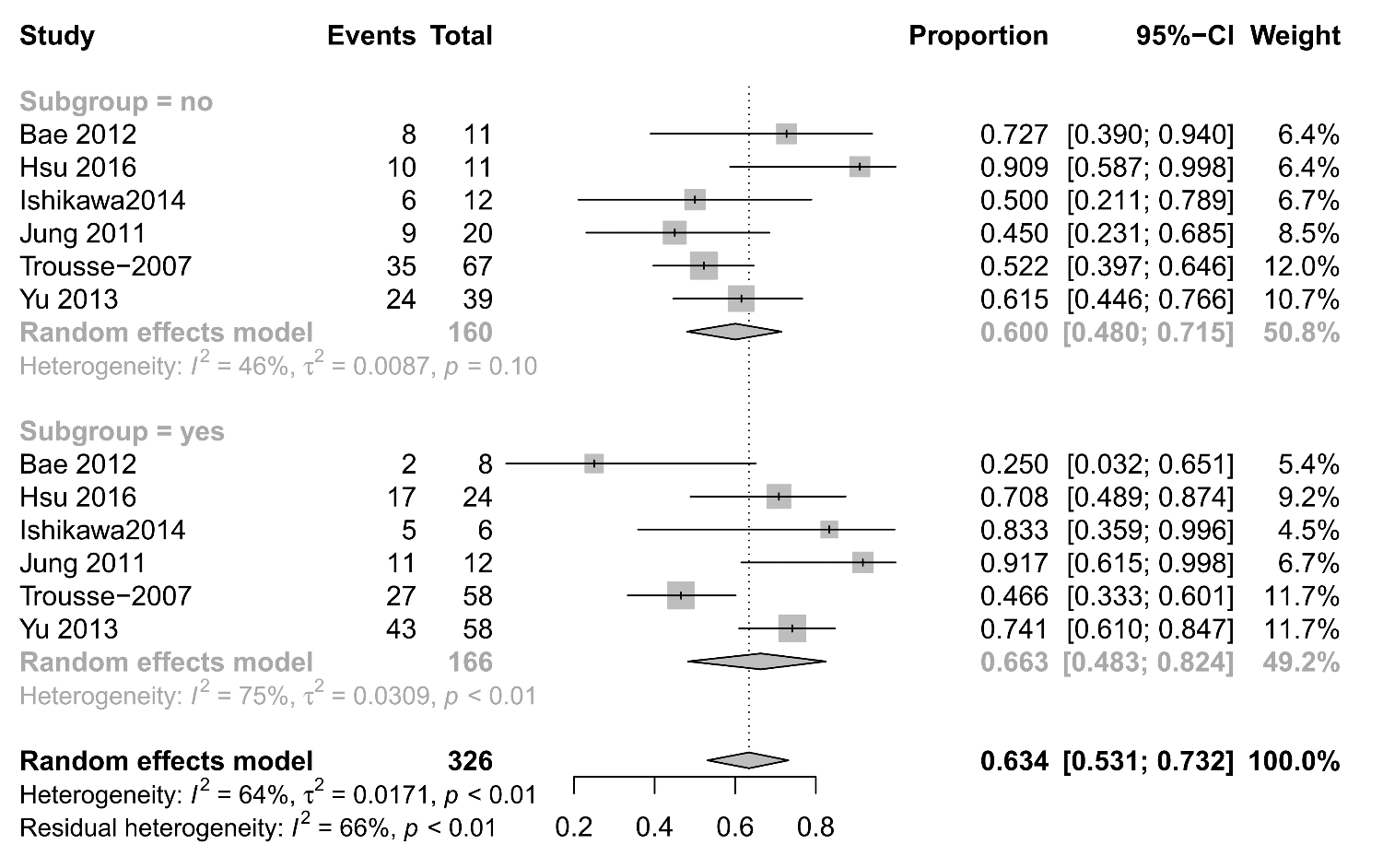
**eFigure 31** Forest chart of additional analysis for 5-year survival rate according to clinical parameters: postoperative adjunctive therapy

**Appendix 13 Effect of various clinical parameters on overall survival**


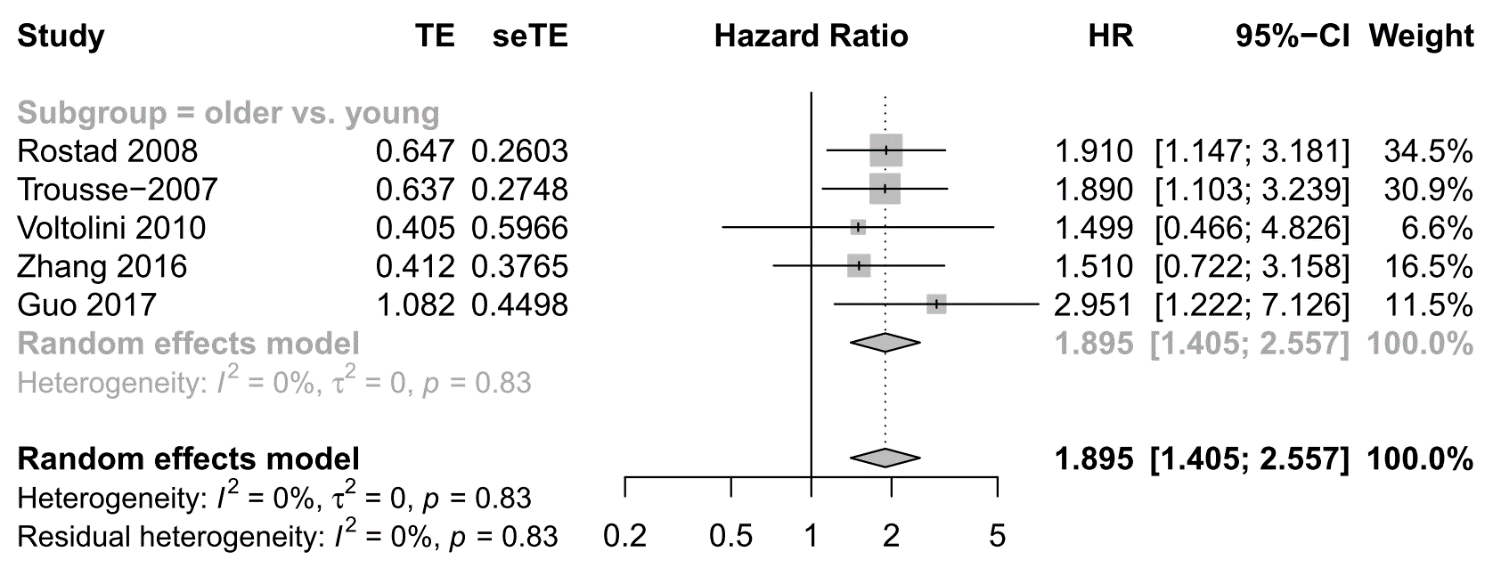
**eFigure 32** Forest chart of additional analysis for overall survival according to clinical parameters: age

**eFigure 33** Forest chart of additional analysis for overall survival according to clinical parameters: gender


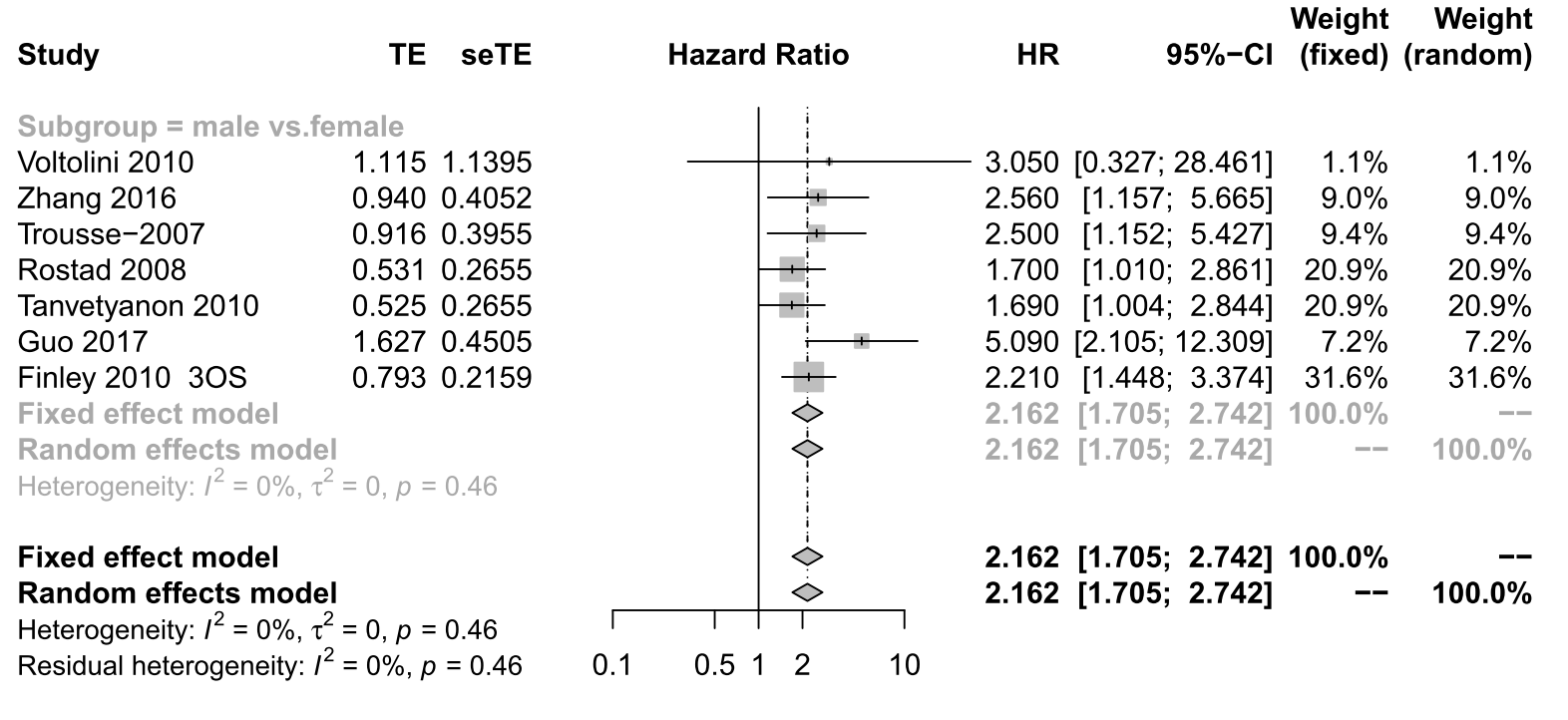
 Result from random-effects model is used.


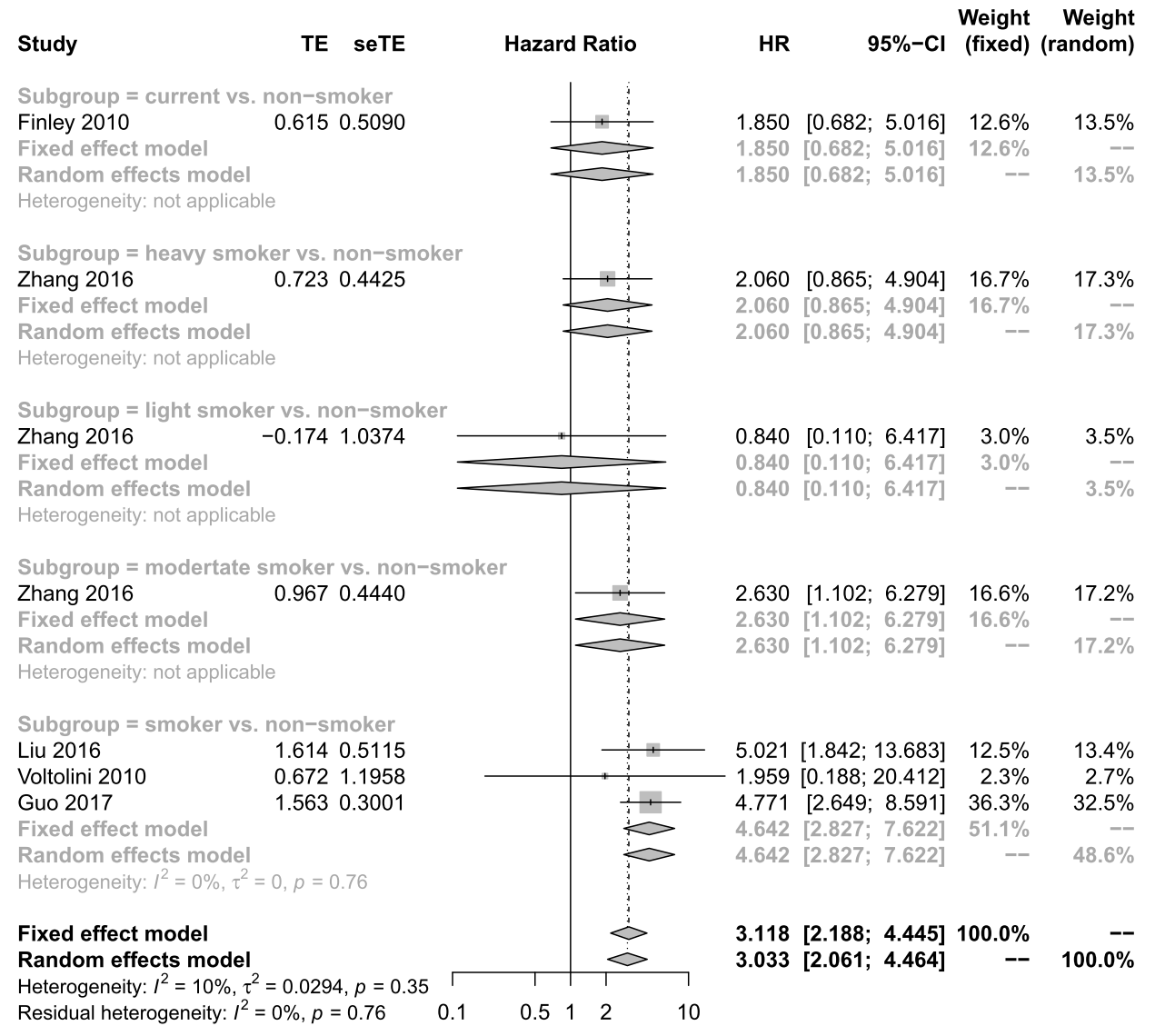
**eFigure 34** Forest chart of additional analysis for overall survival according to clinical parameters: smoking status

Result from random-effects model is used.


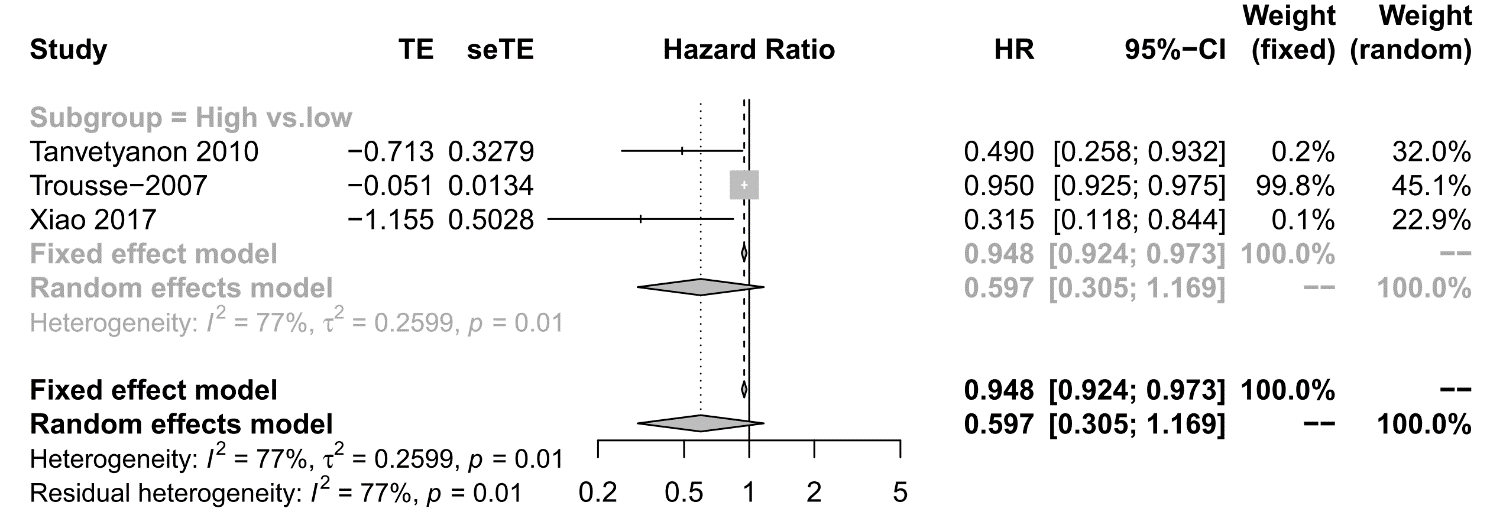
**eFigure 35** Forest chart of additional analysis for overall survival according to clinical parameters: FEV1

Result from random-effects model is used.


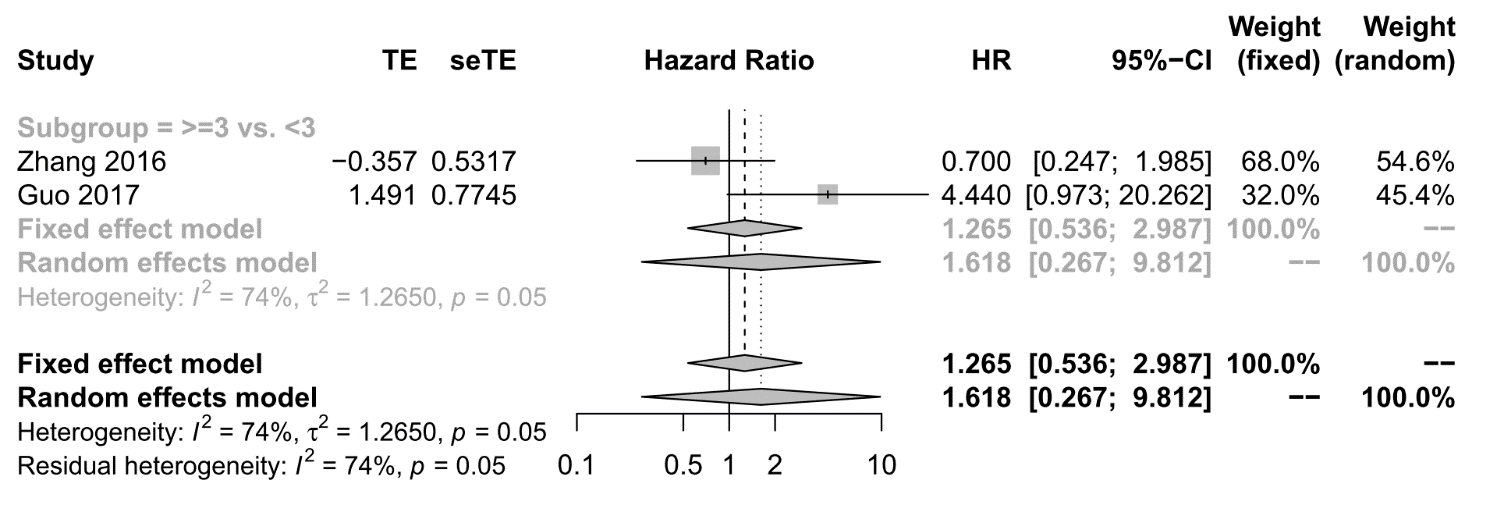
**eFigure 36** Forest chart of additional analysis for overall survival according to clinical parameters: number of tumors

Result from random-effects model is used.


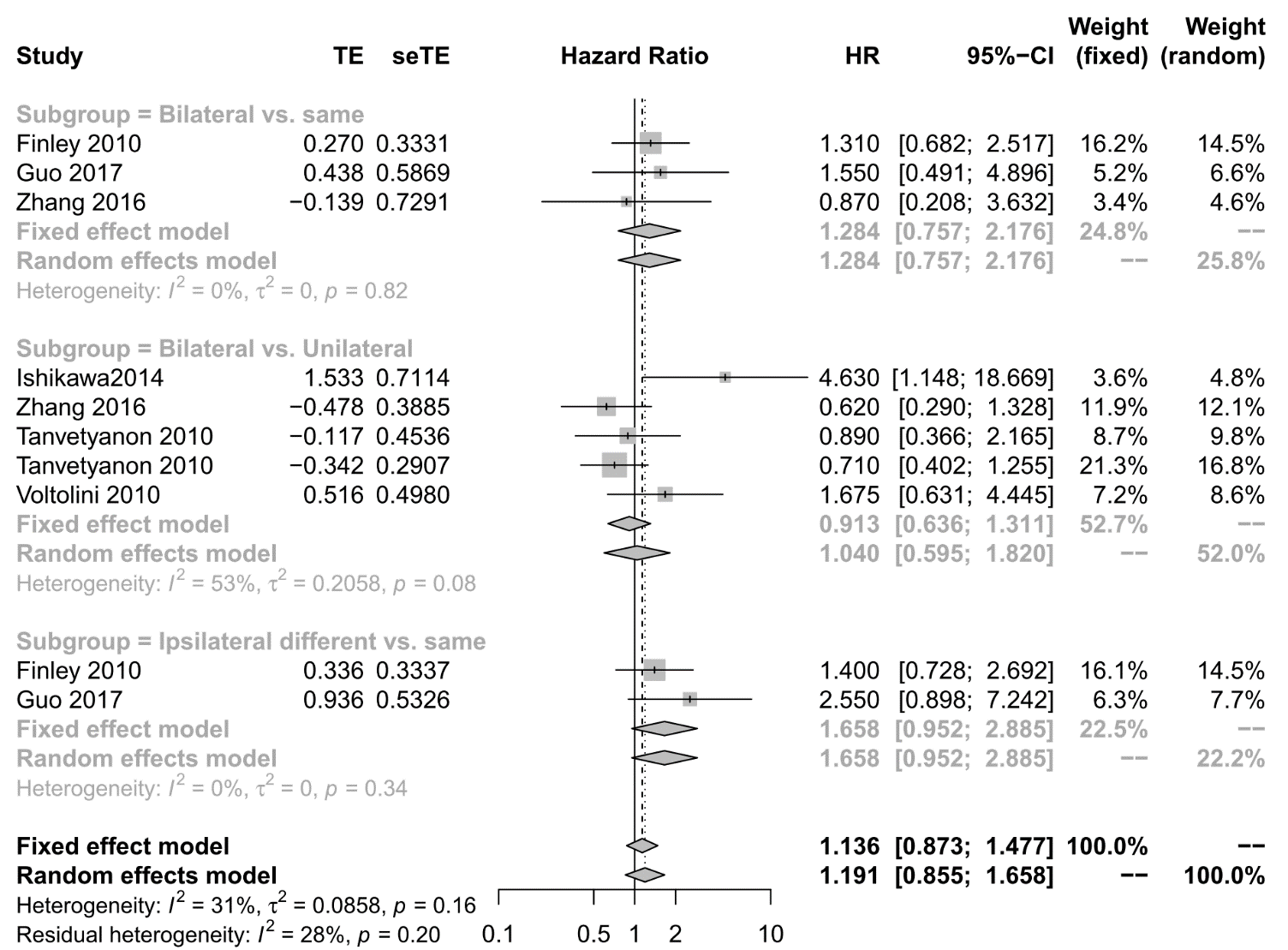
**eFigure 37** Forest chart of additional analysis for overall survival according to clinical parameters: tumor location

Result from random-effects model is used.


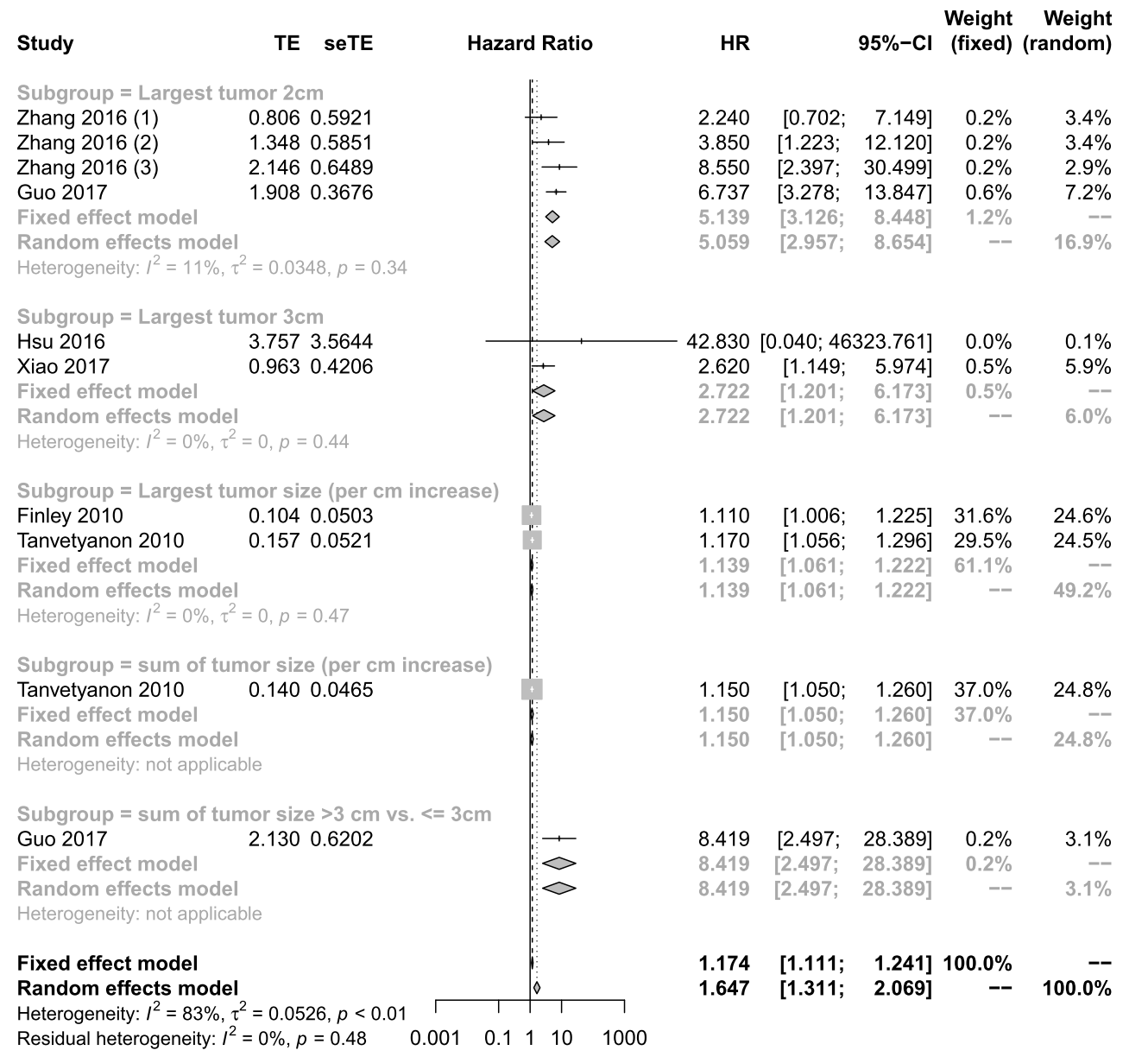
**eFigure 38** Forest chart of additional analysis for overall survival according to clinical parameters: tumor size

Result from random-effects model is used.


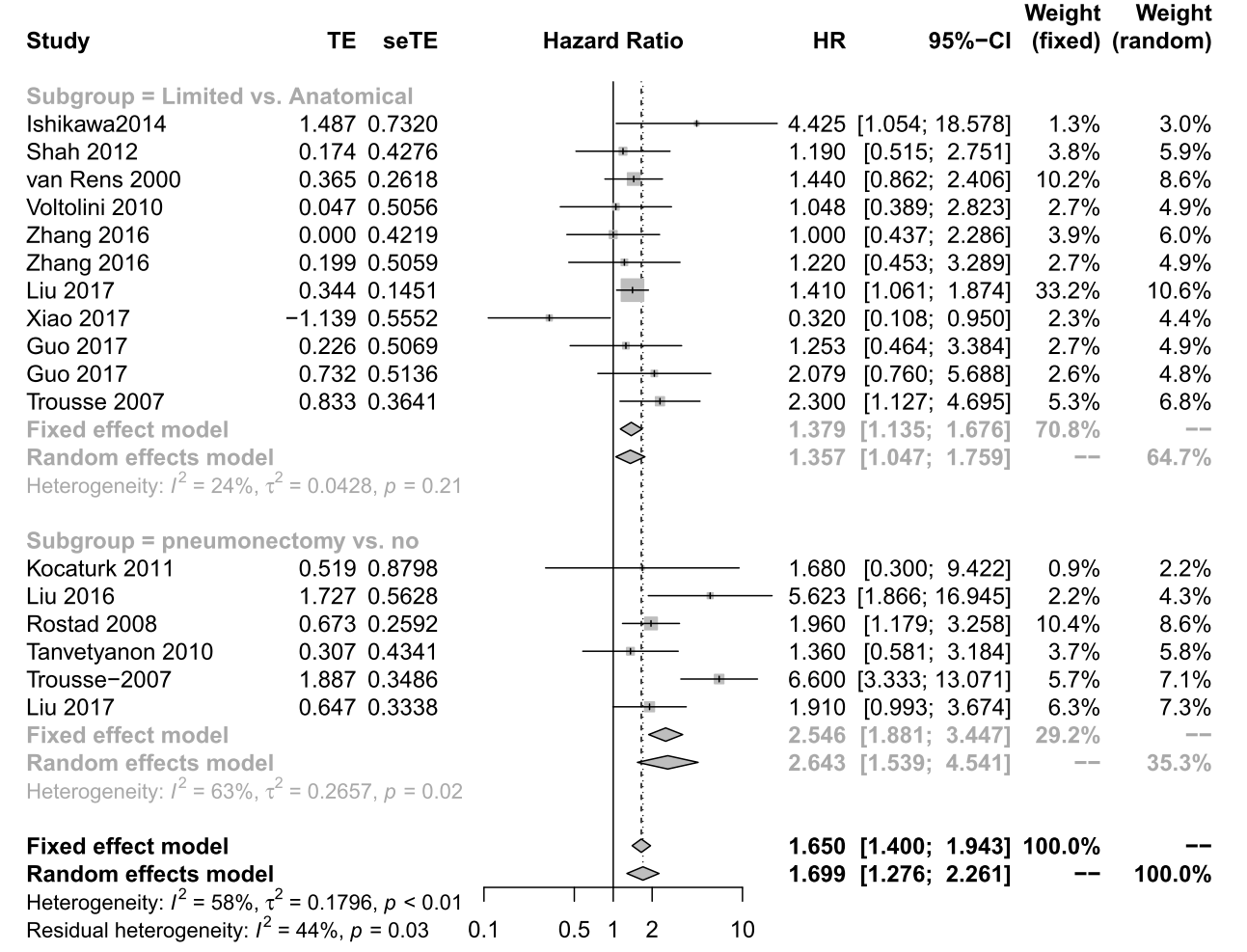
**eFigure 39** Forest chart of additional analysis for overall survival according to clinical parameters: surgery methods

Result from random-effects model is used.


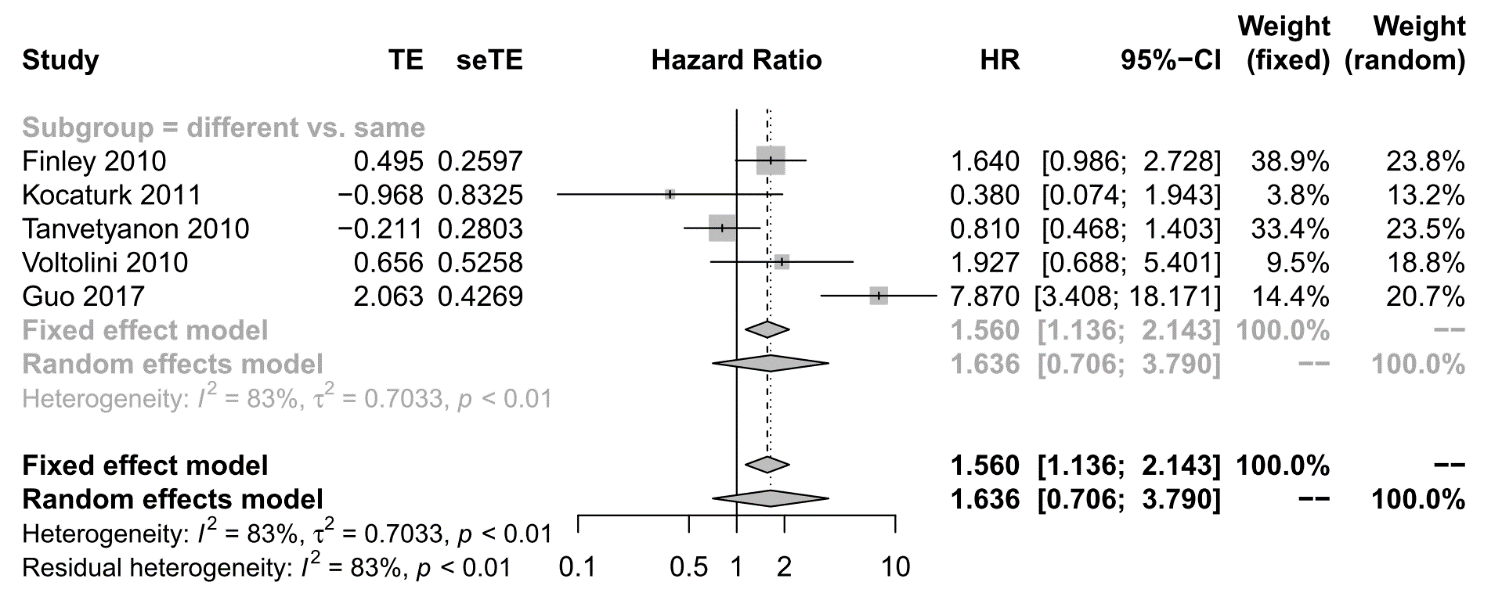
**eFigure 40** Forest chart of additional analysis for overall survival according to clinical parameters: tumor histology

Result from random-effects model is used.

**eFigure 41** Forest chart of additional analysis for overall survival according to clinical parameters: lymph node metastasis


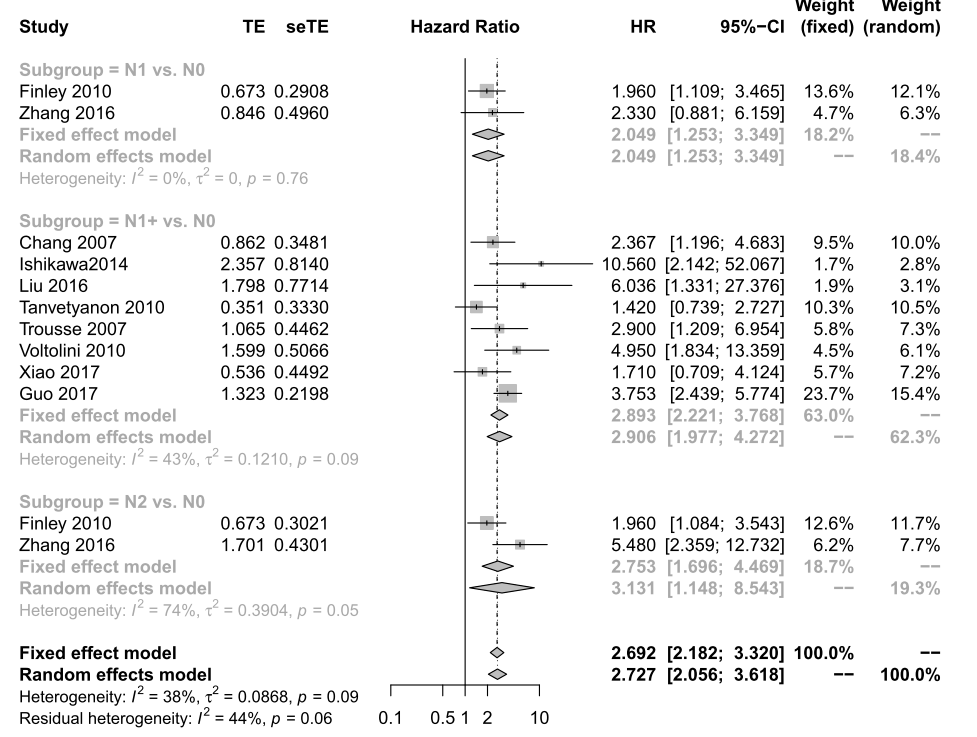
 Result from random-effects model is used.

**eFigure 42** Forest chart of additional analysis for overall survival according to clinical parameters:
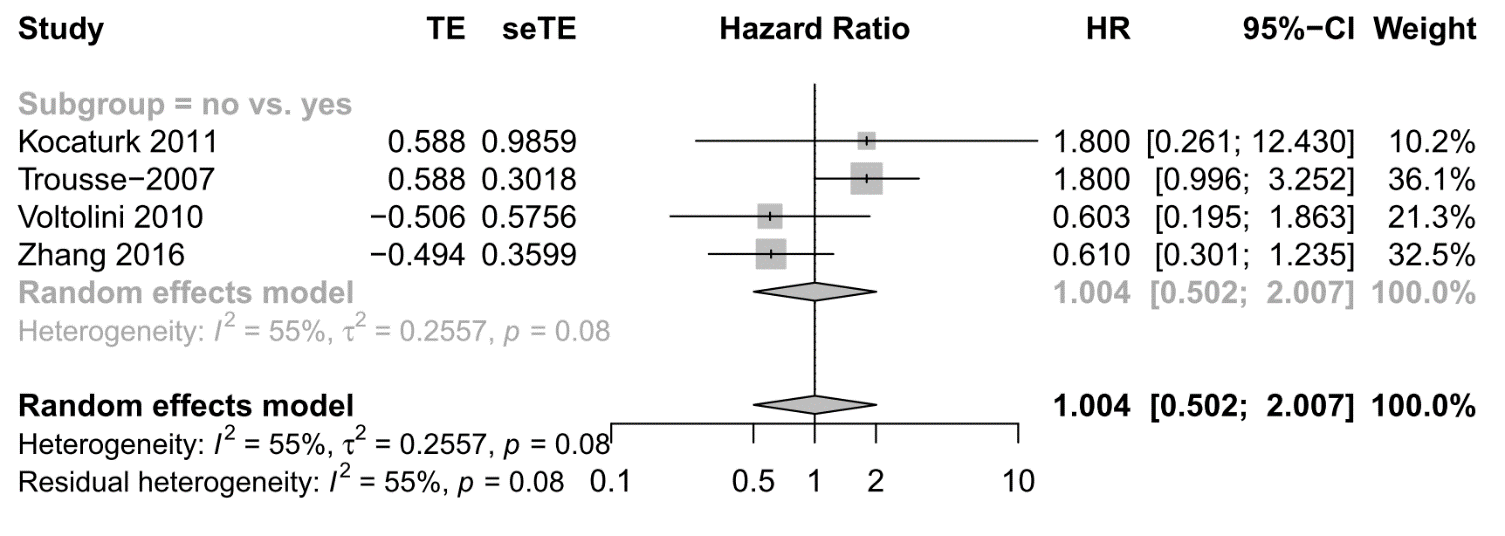
postoperative adjunctive therapy

**Appendix 14 Publication bias**


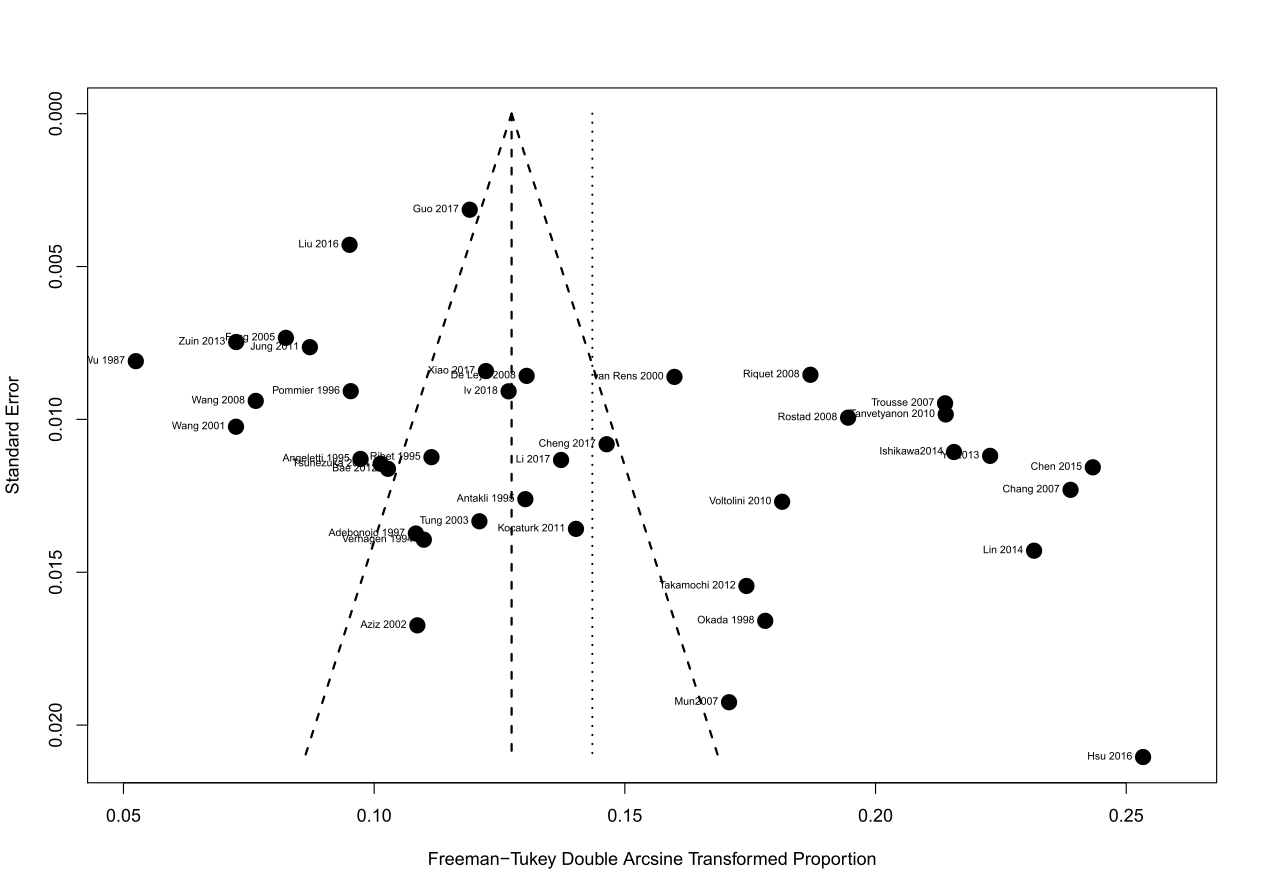
**eFigure 43** Publication bias for sMPLC (Egger’s test *P* =.011)


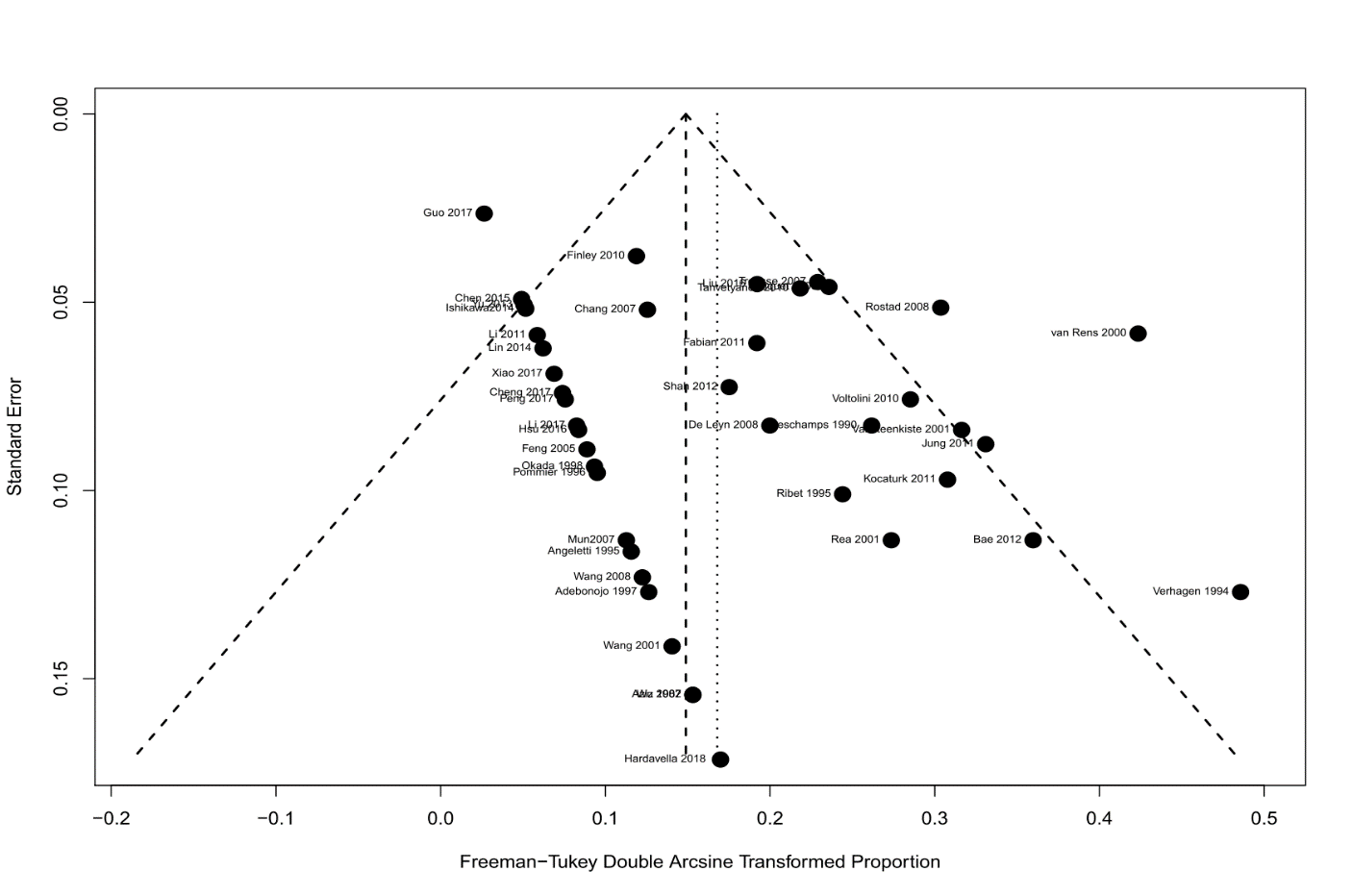
**eFigure 44** Publication bias for postoperative mortality (Egger’s test *P* =.044)

**eFigure 45** Publication bias for 5-years survival rate (Egger’s test *P* =0.001).


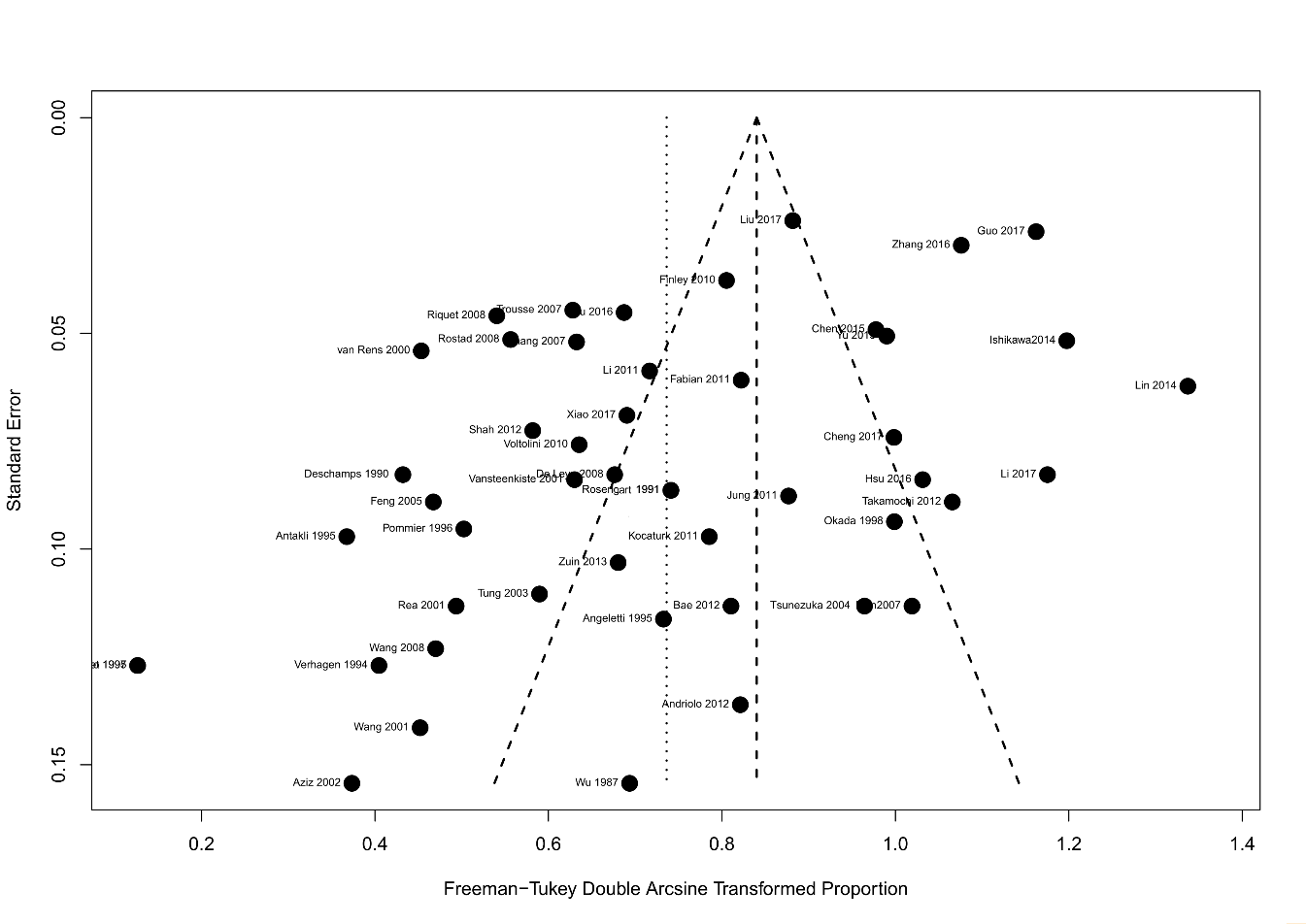

Supplement: Supplementary file 1 — Data S1 [file CAM4-10-507-s001.docx]
